# A proposed core genome scheme for analyses of the *Salmonella* genus

**DOI:** 10.1016/j.ygeno.2019.02.016

**Published:** 2020-01

**Authors:** Madison E. Pearce, Marie A. Chattaway, Kathie Grant, Martin C.J. Maiden

**Affiliations:** a Department of Zoology, University of Oxford, Peter Medawar Building for Pathogen Research, South Parks Road, Oxford OX1 3SY, United Kingdom; b National Institute for Health Research, Health Protection Research Unit, Gastrointestinal Infections, University of Oxford, United Kingdom; c Public Health England, Gastrointestinal Bacteria Reference Unit, 61 Colindale Avenue, London NW9 5EQ, United Kingdom

**Keywords:** Core genome multilocus sequence typing (cgMLST), *Salmonella*, Whole genome sequencing, Core genome scheme, Outbreak

## Abstract

The salmonellae are found in a wide range of animal hosts and many food products for human consumption. Most cases of human disease are caused by *S. enterica* subspecies I; however as opportunistic pathogens the other subspecies (II-VI) and *S. bongori* are capable of causing disease. Loci that were not consistently present in all of the species and subspecies were removed from a previously proposed core genome scheme (EBcgMLSTv2.0), the removal of these 252 loci resulted in a core genus scheme (SalmcgMLSTv1.0). SalmcgMLSTv1.0 clustered isolates from the same subspecies more rapidly and more accurately grouped isolates from different subspecies when compared with EBcgMLSTv2.0. All loci within the EBcgMLSTv2.0 scheme were present in over 98% of *S. enterica* subspecies I isolates and should, therefore, continue to be used for subspecies I analyses, while the SalmcgMLSTv1.0 scheme is more appropriate for cross genus investigations.

## Highlights

• A novel cgMLST scheme is proposed for analysis of the *Salmonella* genus.
• 252 loci are removed from the EBcgMLSTv2.0 scheme to create SalmcgMLSTv1.0.
• SalmcgMLSTv1.0 is more accurate for analyses involving isolates from multiple subspecies.

## 1 Introduction

The salmonellae are gram-negative gammaproteobacteria belonging to the family Enterobacteriaceae [1]. It is estimated that globally *Salmonella* species are responsible for 93.8 million cases of gastroenteritis, 80.3 million of which are foodborne, and 155,000 deaths [2]. Non-typhoidal *Salmonella* are responsible for an estimated yearly loss of 4.07 million disability adjusted life years (DALYs), which is the highest burden of all foodborne infections [3].

The *Salmonella* genus is comprised of two species *Salmonella enterica* (*S. enterica*) and (*S. bongori*) [4,5]. *S. enterica* is further comprised of six subspecies I (enterica), II (salamae), IIIa (arizonae), IIIb (diarizonae), IV (houtenae) and VI (indica) [4,5]. The subspecies were originally determined through differences in phenotypic traits and have since been confirmed as distinct groupings through DNA-DNA hybridisation [6], which has been regarded as the ‘gold-standard’ in genotypic species identification [7]. Microbiologically, bacterial species and subspecies have been identified through morphological and biochemical traits [8]; however, novel subspecies of *Salmonella* have previously been proposed, such as subspecies VII^9^. This was initially identified through multilocus enzyme electrophoresis (MLEE) [9] and was also confirmed as a distinct cluster using genetic approaches [10]. A further three novel subspecies (novel subspecies A, B and C) have been proposed as a result of core genome SNPs analysis [11].

The salmonellae can be found in a wide range of hosts [12], including mammals [13], birds [14], reptiles [15], fish [16] and crustaceans [16]. *S. enterica* subspecies I is primarily associated with warm-blooded animals and the non-subspecies I *Salmonella* are typically associated with cold-blooded animals [12]; however, all of the subspecies and *S. bongori* have been isolated from both warm [17] and cold-blooded animals [18]. Non-subspecies I isolates have been found in high proportions, from animal products for human consumption, including poultry [14], cattle [19], sheep [20], pork [19] and fresh produce [19]. They have also been found in wild animals, including birds of prey [21] and wild boar [17]. Additionally, subspecies I has regularly been isolated from reptilian species [15].

While non-subspecies I *Salmonella* are found in products for human consumption and the environment, they rarely cause human disease [22]. This is because non-subspecies I *Salmonella* are opportunistic pathogens of humans [12], with most cases affecting vulnerable individuals [23,24]; however, in some circumstances these infections have led to complications [25] and death [26]. In countries where people live in close proximity with reptiles the likelihood of *Salmonella* non-subspecies I infection increases. For example, in French Guiana, subspecies IV was responsible for 9.1% of cases compared with 0.3% in metropolitan France [27]. Furthermore, non-subspecies I isolates are regularly identified in reptilian pets [15], reptilian meat for consumption [28] and some ‘traditional medicines’ [29]. These organisms have also been known, on rare occasions, to cause outbreaks in otherwise healthy individuals [30,31], however most *Salmonella* infections are caused by subspecies I^22^.

Foodborne diseases, including *Salmonella*, are monitored by the global surveillance network, PulseNet International [32]. At the time of writing PulseNet International has identified core genome MultiLocus Sequence Typing (cgMLST) as the surveillance system to replace pulsed-field gel electrophoresis [33]. Core genome schemes have several advantages, they can be maintained and shared using online databases [11,34], which makes them easily portable [35] and little to no bioinformatic expertise is required by the end user [33]. Additionally, these schemes are comprised of a fixed set of loci, which are reference free^36^, stable [36,37] and forward and backward compatible [38].

A cgMLST scheme for *Salmonella* has been developed for Enterobase (EBcgMLSTv2.0) [11]. Loci were chosen for inclusion within EBcgMLSTv2.0 if they were present in over 98% of the test genomes, if the coding frames were intact in over 94% and if the number of alleles was not exceptionally different from expected [11]. This ‘relaxed’ approach to a core genome definition allows for rare variants that are missing a locus which would otherwise be considered core [39] and the imperfect nature of draft genomes [36], as well as permitting the removal of elements which are not experiencing direct selection pressures, including repetitive genes and pseudogenes [37]. EBcgMLSTv2.0 has been demonstrated to accurately characterise subspecies I outbreaks [40] and was found to be comparable with single nucleotide polymorphism (‘SNP’) based methods [40], which are currently used within Public Health England (PHE) for both routine surveillance and outbreak investigations [41]. The EBcgMLSTv2.0 scheme has not, however, been systematically assessed for its performance at the genus level.

Here, over 2000 *Salmonella* isolates, representing *S. bongori*, and *S. enterica* subspecies I, II, IIIa, IIIb, IV, VI and suggested subspecies VII, Novel A, B and C were examined using EBcgMLSTv2.0. The aim of this study was to determine if EBcgMLSTv2.0 could be used for future analyses that included isolates of *Salmonella* from multiple species or subspecies and if necessary to create a sub-scheme of the EBcgMLSTv2.0 loci capable of performing this function. EBcgMLSTv2.0 was analysed by how accurately isolates from within the same subspecies clustered and then by how accurately the relationships between the subspecies were represented. These results determined the need for a genus core genome multilocus sequence typing scheme (SalmcgMLSTv1.0), which was created and compared with EBcgMLSTv2.0.

## 2 Materials and methods

### 2.1 Dataset creation and curation

#### 2.1.1 Non-subspecies I isolates

A total of 1275 non-subspecies I isolates were identified with the Enterobase [11] database (13/11/2017) from the metadata provided on deposition. Public Heath England (PHE) provided a further 235 non-subspecies I isolates that were not available in Enterobase (13/11/2017), many of which had underrepresented or novel antigenic formulae. These isolates were assembled using the SPAdes [42] based assembly pipeline incorporated into Enterobase. A final search of Enterobase was conducted (06/03/2018) and identified a further 34 non-subspecies I isolates, creating an initial dataset of 1544. If an isolate did not cluster phylogenetically with the subspecies specified by its metadata and it was not available for reanalysis it was removed, leaving 1480 isolates in this analysis.

#### 2.1.2 Novel subspecies

The subspecies VII, novel A, B and C are regularly misidentified due to a lack of formal recognition [43]. Preliminary cgMLST analyses using GrapeTree [44] showed that the majority of isolates belonging to the same subspecies but not different subspecies, clustered when they had fewer or equal to 2750 allelic differences when using the EBcgMLSTv2.0 [11]. Enterobase was interrogated for previously unidentified isolates which belonged to these novel groups, subspecies VII, novel A, B and C. This search identified a further 14 subspecies VII isolates (Enterobase metadata provided by the depositor: 1 subspecies I, 4 subspecies IV and 9 with none), a further 42 novel B isolates (Enterobase metadata provided by depositor: 3 subspecies II and 39 with none), a further 12 novel C isolates (Enterobase metadata provided by depositor: 11 subspecies II and 1 with none) and no further novel A isolates were identified. Based on these findings the isolates with no metadata and those previously identified as subspecies I were added to analysis (50 isolates) and the metadata for all these isolates were altered for this analysis, increasing the dataset to 1530 non-subspecies I isolates (Supplementary Table S1).

#### 2.1.3 Subspecies I isolates

Due to the overrepresentation of subspecies I a representative subspecies I dataset needed to be generated. Initially, the metadata for subspecies I isolates from PubMLST [34] were downloaded from the database (14,327 on 13/05/2018). These metadata were filtered to ensure that only isolates sequenced within Enterobase [11] were included (3071) so a consistent assembly method was used. All genomes in the data were filtered by ribosomal sequence type (rST) [45] to remove any duplicates (2424) and two isolates, or one where only one was available, were chosen from each serovar. For serovar Newport four isolates were chosen, representing lineages II and III^46^, as these lineages have been shown to be physiologically distinct and are easily distinguished with phylogenetic approaches [46]; no lineage I Newport isolates were available. The chosen isolates were then cross-referenced against Enterobase [11] records and isolates were removed if they had mismatching subspecies or didn't cluster with subspecies I using GrapeTree [44] (Supplementary Table S2). This left a dataset of 556 subspecies I isolates, from 359 subspecies I serovars to be used in conjunction with the 1530 non-subspecies I isolates (Supplementary Table S1).

### 2.2 Enterobase core genome scheme (EBcgMLSTv2.0)

EBcgMLSTv2.0 was used as a starting point for the development of SalmcgMLSTv1.0 as loci had already undergone rigorous filtering and were required to meet strict criteria to be included within this scheme. EBcgMLSTv2.0 is comprised of 3002 loci and has been proposed as a typing scheme for the *Salmonella* genus [11]. EBcgMLSTv2.0 compiled coding sequences from 167 complete *Salmonella* genomes, 82 NCTC genomes which had been sequenced with the PacBio technology [47] and one representative for each of the 288 eBurst groups based on ribosomal multilocus sequence typing (rMLST), which encompassed the diversity of the *Salmonella* genus [40]. The coding sequences identified were grouped into gene clusters and paralogous genes were identified and removed [40]. Representative of each of the 3258 *Salmonella* rMLST sequence types (rSTs) (up to May 2016) were then typed using this scheme [40]. A locus from this scheme was considered core within the *Salmonella* genus if it met three criteria: (i) if it was present in over 98% of the genomes, (ii), if the coding frames were intact in over 94% of the genomes and (iii) if the number of alleles was not significantly different from other loci [11].

### 2.3 Genus core scheme creation (SalmcgMLSTv1.0)

The genome comparator tool, developed for the Bacterial Isolate Genome Sequence Database (BIGSdb) [34] and hosted by pubMLST, was used to create SalmcgMLSTv1.0. The genome comparator tool compares all selected isolates using a predefined scheme, such as an rMLST or cgMLST scheme. Users can modify the minimum percentage identity required to generate a partial match, the minimum percent alignment required to align for partial match and the BLASTN word size that is required to match exactly to initiate an extension. The default values of these parameters are 70%, 50% and 20 respectively. Genome comparator generates an output of all the loci within a defined scheme, where defined allele numbers mark known alleles and missing and incomplete alleles are reported with an ‘X’ and an ‘I’ character respectively.

For the generation of SalmcgMLSTv1.0, 55 isolates were removed from the dataset as they represented duplicate EBcgMLSTv2.0^11^ core genome sequence types (cgSTs), to ensure that no duplicate isolates were included in the analysis and that greater representation of a cgST did not lead to the removal of loci due to its absence in only that cgST.

EBcgMLSTv2.0^11^ was used as an initial starting point for the development of SalmcgMLSTv1.0 as each locus has been rigorously analysed and was subjected to strict criteria before it was included within the scheme [11,40]. The cgSTs of the isolates were split into their respective subspecies, consisting of 556 subspecies I, 266 subspecies II, 303 subspecies IIIa, 424 subspecies IIIb, 318 subspecies IV, 27 subspecies VI, 18 subspecies VII, 42 subspecies novel B, 19 novel C and 54 *S. bongori.* Then their EBcgMLSTv2.0 genome profiles were analysed using the BIGSdb genome comparator tool with default parameters. A locus was removed from EBcgMLSTv2.0 if it was missing in over 2% of the isolates and over 5 isolates per subspecies. The removal of loci missing in over 2% of isolates was chosen as it matched the 98% inclusion criteria chosen for EBcgMLSTv2.0. The locus also needed to be missing in over 5 isolates, due to the small numbers of some isolates within the subspecies groups, this allowed for the creation of an accurate but conservative genus scheme. As a locus needed to be present in over 5 isolates per subspecies, novel subspecies A was removed from this analysis. The number of genes which needed to be removed from the EBcgMLSTv2.0^11^ per subspecies were analysed, as was the average number of genes missing per isolate per subspecies and the range of missing loci per subspecies. Graphs were produced using RStudio [48].

### 2.4 Grape tree [44] analysis

GrapeTree [44] has a minimum spanning tree algorithm that is capable of reconstructing genetic relationships, even with high levels of missing data [44] and was used to compare EBcgMLSTv2.0^11^ with SalmcgMLSTv1.0, using all of the subspecies isolates (556 subspecies I, 278 II, 308 IIIa, 442 IIIb, 326 IV, 28 IV, 22 VII, 3 novel A, 48 novel B, 19 novel C and 56 *S. bongori*) (Supplementary Table S1). Minimum spanning trees were generated using both EBcgMLSTv2.0^11^ and SalmcgMLSTv1.0 and the nodes were labelled with the respective subspecies. The branches were collapsed until nodes which represented different subspecies merged, to examine which of the subspecies were most closely related. This was repeated until all subspecies merged into a single node.

### 2.5 Structure ^[49,50]^ Analysis

The Structure [49,50] algorithm has previously demonstrated the accurate inference of population structure in comparison with phylogenetic results [49]. It does this through identifying populations within the provided dataset and then assigning individuals to their most similar populations. In order to assign individuals to populations the algorithm analyses the distribution of different variants within their profiles, which are used to create genetic clusters. Similar variation patterns are grouped iteratively using Bayesian algorithms. Isolates are initially assigned to random groups and then reassigned based on their variation patterns and frequencies, using a Markov Chain Monte Carlo *(*MCMC) estimation [49] process.

Structure analyses were performed on a subset of 73 isolates, 7 representatives of each of the included subspecies, except novel subspecies A which only had 3 representatives available. All the non-subspecies I isolates were chosen at random, ensuring there were no duplicate core genome profiles. Whereas subspecies I isolates were chosen for diversity, with two isolates randomly chosen from clade B^51^ and Typhi/Paratyphi A^51,52^ and three isolates chosen from clade A [51], two of these represented the serovars responsible for the most disease globally – serovars Typhimurium and Enteritidis and one was chosen at random. The Structure algorithm was run with a burn-in of 100,000 and an MCMC of 200,000 and the maximum number of populations (K) assumed was increased from the initial number of subspecies (11), until lower order taxonomical groups or no further populations were observed. Outputs from the analyses were edited using the Distruct tool [53].

The Structure analysis was performed with the alleles frequencies independent model, as the subspecies represent genetically diverse and distinct populations [11]. Both EBcgMLSTv2.0 and SalmcgMLSTv1.0 were analysed, in order to determine if there were any differences between the two schemes. The EBcgMLSTv2.0 analysis was based on 2991/3002 core genes as 11 were removed due to the presence of paralogs in some of the isolates at these loci (Supplementary Table S3). While the SalmcgMLSTv1.0 analysis was based on 2732/2741 core genes as 9 were removed due to the presence of paralogs (Supplementary Table S3).

## 3 Results

### 3.1 *Salmonella* genus core genome scheme (SalmcgMLSTv1.0)

**Fig. 1:**
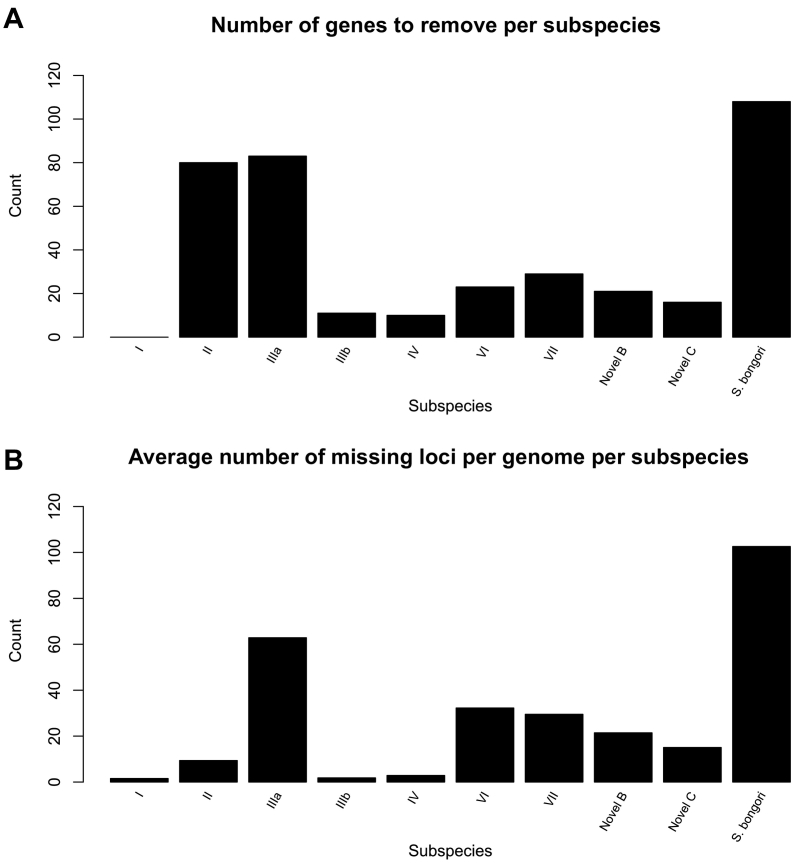
a and b Graphs showing the number of genes to be removed per subspecies (A) and the average number of missing genes per genome per subspecies (B). Graphs showing the spread of the missing genes across the subspecies over 559 subspecies I isolates, 266 subspecies II, 303 subspecies IIIa, 424 subspecies IIIb, 318 subspecies IV, 27 subspecies VI, 18 subspecies VII, 42 subspecies novel B, 19 novel C and 54 *S. bongori.* A: The number of genes to be removed per subspecies. This graph shows that the number of genes which need to be removed from EBcgMLSTv2.0 were highly variable across the genus. EBcgMLSTv2.0 was accurate for subspecies I with no genes missing in over 2% of the genomes analysed, however the rest of the subspecies had genes which need to be removed in order to create an accurate genus scheme. *S. bongori*, subspecies IIIa and II all had over 50 genes which were missing in at least 2% of the genomes analysed. B: The average number of missing genes per genome per subspecies. This graph shows that there is variability among the subspecies of the average number of missing genes per genomes. Both *S. bongori* and subspecies IIIa had a high number of missing genes per genome, while subspecies I, IIIb and IV had much lower levels of missing genes, suggesting that EBcgMLSTv2.0 was more accurate for these subspecies.

A locus was removed from EBcgMLSTv2.0^11^ for the genus scheme if it was missing in over 2% of genomes and more than 5 genomes within *S. bongori* or any of the *S. enterica* subspecies. No locus had to be removed from the subspecies I analysis, as all loci were present in over 98.2% of isolates. For non-subspecies I isolates, between 10 and 108 loci were removed per subspecies (Fig. 1). The average number of missing loci per isolate varied widely, from 1.5 loci missing on average within subspecies I to 102.6 within *S. bongori* (Fig. 1b). Due to some loci meeting the deletion criteria in multiple subspecies (Supplementary Table S4), a total of 252 loci were removed from EBcgMLSTv2.0 to create SalmcgMLSTv1.0 (Supplementary Table S5). The overall variation of isolates within a subspecies also differed, as subspecies IIIb, IV, novel B, novel C and VII isolates all had a smaller range of missing loci per isolate than those of subspecies I and all had less than 1% of the EBcgMLSTv2.0 loci missing on average. In comparison subspecies IIIa, VI and *S. bongori* isolates all had a larger range of loci missing and had more than 1% of the EBcgMLSTv2.0 loci missing on average. Subspecies II isolates showed a greater range but had less than 1% of the EBcgMLSTv2.0 loci missing on average, suggesting a large diversity and variability of isolates within subspecies II isolates (Table 1).

### 3.2 Grape tree [44] analysis

**Fig. 2:**
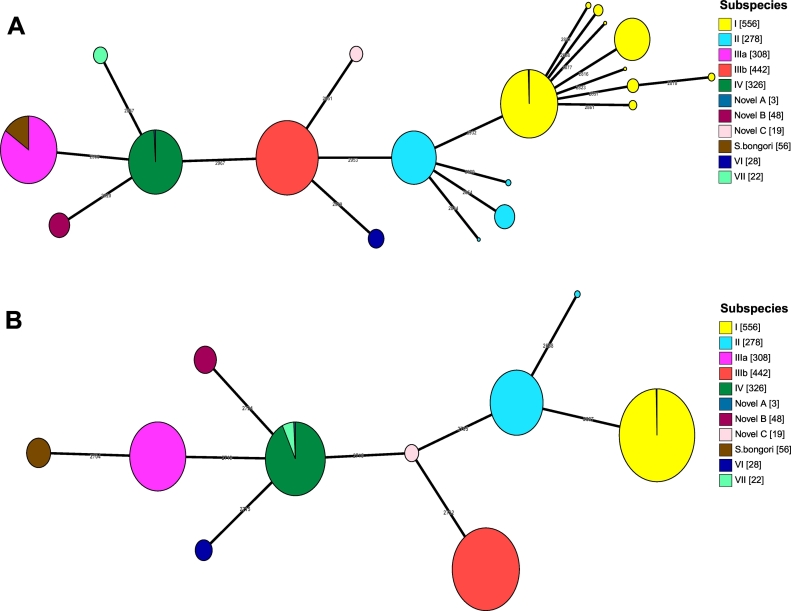
a and b EBcgMLSTv2.0 minimum spanning tree (A) and genus core genome minimum spanning tree (B). Minimum spanning Trees created with 2086 isolates, consisting of 556 subspecies I, 278 II, 308 IIIa, 442 IIIb, 326 IV, 28 VI, 22 VII, 3 novel A, 48 novel B, 19 novel C and 56 *S. bongori* were constructed via the GrapeTree [44] programme. The nodes were then collapsed until two subspecies had merged with nodes representative of another subspecies. A: EBcgMLSTv2.0 minimum spanning tree at 2804/3002 nodes collapsed. The first subspecies to merge were *S. bongori* and subspecies IIIa at 2798/3002 nodes collapsed, followed by novel subspecies A and subspecies IV. The initial merging of *S. bongori* and IIIa indicated that EBcgMLSTv2.0 wasn't completely accurate with the biology of the *Salmonella* genus. B: SalmcgMLSTv1.0 minimum spanning tree at 2640/2750 nodes collapsed. The first subspecies to merge were novel subspecies A and subspecies IV at 2585/2750 nodes collapsed, followed by subspecies VII merging with the subspecies IV, novel subspecies A complex. This merging was expected by the biology of the *Salmonella* genus, which shows that these 3 subspecies are among the closest related. This suggested that the removal of the 252 loci created a scheme which was more biologically accurate when performing analyses at the genus level.

The overall topologies of the two minimal spanning trees were congruent, despite the removal of the 252 loci to create SalmcgMLSTv1.0 (Fig. 2). The relative number of differences that needed to be collapsed for all isolates of the same subspecies to form a single node and the order in which nodes for subspecies merged together was changed. SalmcgMLSTv1.0 grouped almost all isolates of the same subspecies into single nodes relatively more quickly (2585/2750 loci collapsed) than EBcgMLSTv2.0^11^ (2857/3002 loci collapsed). All subspecies I isolates and most subspecies II isolates were members of a single node consisting of isolates from their own subspecies using SalmcgMLSTv1.0 when two other subspecies had merged, conversely many subspecies I and II isolates were still separate using EBcgMLSTv2.0. Using the EBcgMLSTv2.0 the first nodes to merge were *S. bongori* and subspecies IIIa, when branches were collapsed to 2798 differences (Fig. 2). This was unexpected because all *S. enterica* subspecies share a more recent common ancestor with each other than they share with *S. bongori*^9–11^. It is likely to have occurred because *S. bongori* and IIIa had the most and second most missing genes respectively, therefore there were fewer loci in which they could differ. The next two nodes to merge using EBcgMLSTv2.0 were subspecies novel A and IV at 2804 differences, which was consistent with expectations as these subspecies have been previously described as being closely related phylogenetically [11]. In comparison, the first nodes to merge using SalmcgMLSTv1.0 were subspecies novel A and IV at 2585 differences (Fig. 2b) which fits with previous phylogenetic observations [11]. The next nodes to merge were subspecies VII with the IV/novel A group at 2640 differences and again this fits with previous phylogenies of these organisms, showing that these three subspecies are closely related [11].

**Table 1:** showing the spread of missing loci per subspecies. This table showed that there is a general correlation between the average number of missing loci per isolate per subspecies and the number of loci which were not present in 98% of the genomes analysed. The main exception to this observation was subspecies II, which showed a relatively low average number of missing loci but had 80 loci meet the criteria for removal, suggesting that there was a significant amount of diversity within subspecies II. The results also showed that there was a considerable spread among the subspecies in the range of missing loci per isolate with some having a range of just 3, while others had over 200, suggesting that some of the subspecies were more conserved than others.

subspecies	Number of genomes in analysis	Number of loci deleted	Average number of missing loci in cg per isolate	Average percent of cg loci missing per isolate	Range of missing cg loci per isolate
I	557	0	1.54	0.05%	0–96
II	266	80	9.38	0.31%	0–213
IIIa	303	83	62.85	2.09%	51–264
IIIb	424	11	1.81	0.06%	0–67
IV	318	10	2.86	0.10%	1–52
Novel Subspecies B	42	21	21.43	0.71%	21–31
Novel Subspecies C	19	16	15.05	0.50%	11–30
*S. bongori*	*55*	108	102.58	3.42%	80–202
VI	27	23	32.26	1.07%	19–271
VII	18	29	29.5	0.98%	29–32

### 3.3 Structure ^49,50^ analysis

**Fig. 3:**
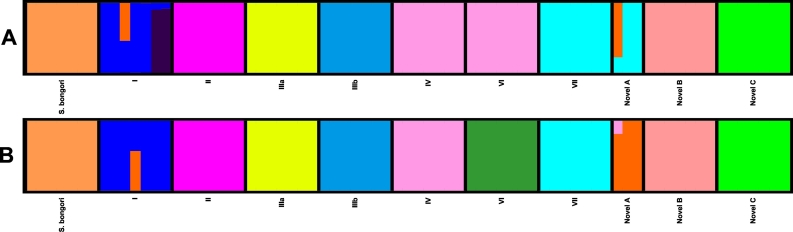
a and b Structure analysis of 11 populations using EBcgMLSTv2.0 (A) and Structure analysis of genus core genome scheme (B). Structure ^49,50^ plots of 73 isolates: 7 subspecies I, II, IIIa, IIIb, IV, VI, VII, novel B, novel C and *S. bongori* and 3 novel A isolates, edited using the Distruct tool [53]. These plots were created with a burn-in of 100,000, an MCMC of 200,000 and a K of 11 and compared EBcgMLSTv2.0 with SalmcgMLSTv1.0. A: Plot showing that at a K of 11 the Structure [49,50] algorithm is incapable of identifying all the *S. enterica* subspecies and *S. bongori* when using EBcgMLSTv2.0. The algorithm finds greater statistical differences among isolates from subspecies I than it finds between subspecies IV and VI and subspecies VII and 2 of the 3 novel A isolates. This results in subspecies IV and VI forming a single population and subspecies VII and 2 of the 3 novel A isolates forming another. The subspecies I serovars Typhi and Paratyphi A are identified as a separate population with very minor admixture from the other subspecies I serovars. Subspecies I serovar Oranienburg is identified as admixed between subspecies I and the novel A isolate, this could be due to the recombinant nature of subspecies I isolates from clade B, such as Oranienburg. B: Plot showing that at a K of 11 the Structure [49,50] algorithm identified all the *S. enterica* subspecies and *S. bongori* as distinct populations when using SalmcgMLSTv1.0. All of the isolates are highly related to other isolates from their own subspecies, with very little admixture. The largest exception was the subspecies I serovar Oranienburg isolate, which was admixed between subspecies I and novel A isolates, this is possibly due to recombination as subspecies I isolates belonging to clade B are quite variable. One isolate within subspecies novel A also showed a small degree of admixture with subspecies IV, this is most likely due to a shared ancestry between these subspecies, as they are closely related.

The ability of the Structure [49,50] algorithm to identify the *S. enterica* subspecies and *S. bongori* differed between the two core genome schemes (Fig. 3). When a Structure analysis was performed, with a K of 11, on EBcgMLSTv2.0 the algorithm was incapable of identifying all of the *S. enterica* subspecies, particularly subspecies IV and VI and subspecies VII and novel A*.* (Fig. 3). Analysing SalmcgMLSTv1.0, under the same parameters, the algorithm identified all of the subspecies and *S. bongori* as distinct populations (Fig. 3b).

Structure only identified 9 of the 11 subspecies at a K of 11 when performed on EBcgMLSTv2.0 (Fig. 3). Subspecies II, IIIa, IIIb, novel B, novel C and *S. bongori* were all identified as distinct populations. Subspecies IV and VI isolates were indistinguishable by the programme, as were subspecies VII and 2 of the novel A isolates. EBcgMLSTv2.0 also found appreciable admixture within subspecies I serovar Oranienburg and the other novel A isolate, which could be due to recombination between these isolates, although recombination between subspecies is rare it has been previously demonstrated [51]. Structure also identified the subspecies I serovars Typhi and Paratyphi A isolates as predominantly distinct populations, with a small level of admixture with the other subspecies I isolates, when EBcgMLSTv2.0 was used. The Structure algorithm resolved all of the *S. enterica* subspecies and *S. bongori* using EBcgMLSTv2.0; however, it required a larger number of populations (K = 17) and led to a higher level of admixture within subspecies I (Supplementary Fig. S1). Similar population structures and levels of admixture within the populations are also observed using the genus scheme with a higher number of populations (K = 16).

When Structure analysis was performed using SalmcgMLSTv1.0 the 73 isolates analysed clustered into their respective subspecies without admixture, except for the subspecies I serovar Oranienburg isolate and one of the novel subspecies A isolates (SAL_JA5200AA) (Fig. 3b). Similarly to EBcgMLSTv2.0, the subspecies I serovar Oranienburg isolate displayed considerable admixture between subspecies I and novel subspecies A, again this is most likely due to recombination [54]. The novel subspecies A isolate showed minor admixture with subspecies IV, this is most likely due to a shared ancestry, as these subspecies are closely related [11].

### 3.4 Availability of the SalmcgMLSTv1.0 scheme

This is scheme is available through PubMLST at pubmlst.org/salmonella and can be used for analyses on isolates within that database.

## 4 Discussion

By removing 252 loci that were missing in over 2% of isolates and more than 5 isolates in any of the subspecies or *S. bongori* from the EBcgMLSTv2.0^11^ scheme, a *Salmonella* genus core genome scheme was generated (SalmcgMLSTv1.0) (available at pubmlst.org/salmonella). This equated to the removal of 8.39% of the EBcgMLSTv2.0 scheme. The removal of these loci was undertaken because all loci within a scheme should be present within all genomes analysed by that scheme to enable direct comparisons [55]. These loci were originally included because EBcgMLSTv2.0 used a representative genome for each of the 3258 rSTs which were available in the Enterobase database at the time [40]. As subspecies I is isolated and sequenced more often than all of the other species and subspecies combined [2], most of these rSTs would have belonged to subspecies I. Consequently, the other subspecies and *S. bongori* did not constitute a large enough proportion of isolates for loci that are frequently missing within one or more of these subspecies or *S. bongori* to be removed. This is supported by the results presented here, which demonstrated that all loci were present in at least 98% of subspecies I isolates tested. The creation of the SalmcgMLSTv1.0 scheme from the EBcgMLSTv2.0 scheme allows for compatibility between the schemes, as the loci are defined and identified using the same approaches. Compatibility between the schemes enables easy comparison and communication of the loci.

The 252 loci were not uniformly missing from the other subspecies and *S. bongori*. Fewer than 30 loci met the criteria for removal within subspecies IIIb, IV, VII, novel B and novel C, which equated to them missing fewer than 1% of the EBcgMLSTv2.0 loci. The other subspecies II, IIIa and VI and *S. bongori* had 80 or more loci that met the criteria for removal. Within these subspecies over 2.5% of the EBcgMLSTv2.0 loci were missing and *S. bongori* genomes were on average missing over 3.5% of the EBcgMLSTv2.0 loci. This suggests that these loci were never present or have since been lost within these subspecies and should be removed when analysing the *Salmonella* genus as a whole.

While both schemes were capable of identifying and clustering all of the subspecies and *S. bongori*, the removal of these 252 loci led to isolates from the same subspecies and *S. bongori* forming single node groups more quickly when the SalmcgMLSTv1.0 loci were analysed with GrapeTree than the EBcgMLSTv2.0 loci. This is demonstrated as using SalmcgMLSTv1.0 all subspecies I isolates and the majority of subspecies II isolates had formed single nodes at the point when two other subspecies had merged, conversely many subspecies I and II isolates were still separate using EBcgMLSTv2.0. This is most likely because the removed loci were driving the diversity within subspecies I and II, which were the final two subspecies to form single subspecies nodes when analysed using both schemes. Using EBcgMLSTv2.0 the first nodes to merge belonged to *S. bongori* and subspecies IIIa, which did not fit with previous findings that the *S. enterica* subspecies share a common ancestor [10,56]. *S. bongori* and subspecies IIIa most likely merged first because they had the most and second most missing loci respectively and therefore their overall diversity was reduced as there were fewer loci in which they could differ. The considerable number of loci missing from subspecies IIIa when compared with the other *S. enterica* subspecies suggests that it could be a separate *Salmonella* species and not a member of the *S. enterica* species. Further work would, however, be needed in order to confirm this observation.

In comparison, the first subspecies to merge using SalmcgMLSTv1.0 were subspecies novel A, IV and VII, which fits with previous phylogenies of these organisms that showed these subspecies were closely related [11]. While these results do not alter the capability of the schemes to detect and cluster isolates of the same subspecies with each other, they are important for exploring the relationships between species and subspecies.

Missing genes can create problems within the construction of phylogenetic trees, as they reduce the power of programs to resolve relationships [57]. Increased proportions of missing genes can reduce the resolution and increase the number of stochastic errors when using maximum likelihood, Bayesian inference and maximum parsimony approaches [57,58]. It has been demonstrated that a greater level of missing data can lead to a larger long branch attraction effect, with faster evolving organisms becoming increasingly attracted to the outgroup [57]. Furthermore, including genes that were missing in some organisms within an analysis reduced the ability of these approaches to detect multiple substitutions within a single position, while complete removal of a gene had only stochastic effects on the length of the branch [57]. These errors, which are introduced because of missing data, highlight the need for a genus core genome scheme to contain as few loci that are consistently missing in one or more species or subspecies as possible.

The absent genes also created problems when a subset of the subspecies and *S. bongori* were analysed using a statistical approach – the Structure [49,50]algorithm. While both core schemes were capable of identifying all of the subspecies and *S. bongori* the SalmcgMLSTv1.0 scheme was more efficient for resolving the subspecies into individual clusters and did so with less admixture. Structure identified all of the 11 subspecies when applied to the isolates typed using SalmcgMLSTv1.0 when applied to a population size of 11. In comparison, the 11 subspecies were not identified within the same isolates typed using EBcgMLSTv2.0 until the algorithm was applied to a population size of 16. When EBcgMLSTv2.0 was analysed with a population size of 11 it initially identified subspecies IV and VI, and subspecies VII and 2 of the novel A isolates as belonging to the same populations. This is because when the isolates were analysed using EBcgMLSTv2.0 Structure identified subspecies I serovars Typhi and Paratyphi A and subspecies I serovar Oranienburg and one isolate from subspecies novel A as distinct populations, over the other subspecies. It is possible that this was observed because while the diversity of all subspecies is increased by the 252 loci that were removed to create SalmcgMLSTv1.0, it has a greater effect on subspecies I, as these loci are more frequently missing within the other subspecies and *S. bongori.* Therefore, the differences created between subspecies I and *S. bongori* and the other subspecies within these 252 loci mean the algorithm falsely identified the clades [51,52] of subspecies I [59] as distinct populations before identifying all of the subspecies.

When performing analyses using the cgMLST approach the scheme should be designed for the collection of isolates being analysed, which need to be a phylogenetically coherent group. For example, human campylobacteriosis is caused by *Campylobacter jejuni* and *C. coli*, therefore for surveillance and outbreak detection a cgMLST scheme that included only these species was developed [60]. Whereas within *Neissiera*, a genus core scheme has been created to explore phylogenetic clustering and genetic exchange among species [61], while a scheme has also been created for *Neisseria meningitidis*^36^ for species specific investigations. This hierarchical approach has been proposed to ensure that the biological question asked and the relatedness of the organisms analysed determine the scheme to be used [62]. Different cgMLST schemes can be used depending on the relatedness of the organisms, as subspecies specific schemes will have higher resolution, while species and genus schemes allow for comparisons from wider groups of organisms such as genera or families. The scheme developed here was generated to facilitate efficient and effective analyses of isolates from across the genus. It was also demonstrated that EBcgMLSTv2.0 was accurate for subspecies I, as all loci were present in at least 98% of isolates. Furthermore, previous work demonstrated that EBcgMLSTv2.0 was capable of analysing and comparing outbreak isolates within a multi-country subspecies I serovar Enteritidis outbreak [40]. As the vast majority of outbreaks are caused by subspecies I isolates EBcgMLSTv2.0 should continue to be used for outbreak analysis, as it is capable of performing subspecies I outbreak analyses and the increased number of loci provides increased resolution for analysis. The SalmcgMLSTv1.0 scheme should be used for the comparison of isolates from across the *Salmonella* genus.

These results provide a starting point for the creation of such a hierarchical system, where the core genome scheme used fits the isolates being analysed. A modular system of subspecies and species-specific schemes will enable users to create custom schemes, based on the particular investigation they are performing. EBcgMLSTv2.0^11^ is a functional subspecies I scheme, the removal of the 252 loci suggested here created a genus scheme (SalmcgMLSTv1.0) and a *S. enterica* scheme can easily be created by including the loci listed here that are missing specifically from *S. bongori*. The SalmcgMLSTv1.0 scheme has been made publicly available at pubmlst.org/salmonella. Further analyses are needed to create schemes specific to the other *S. enterica* subspecies and *S. bongori*, as it is likely that loci that were excluded from EBcgMLSTv2.0, due to their absence in subspecies I, are routinely present in other subspecies. These schemes could be created and implemented in such a way as to enable them to be used in conjunction, allowing the user to create custom schemes based on their own needs. For example, a subspecies IV, VII and novel A scheme could be created to further explore the shared ancestry and subsequent divergence of these three subspecies. Further examination should also be performed on *S. enterica* subspecies IIIa, in order to determine if this subspecies should be reclassified as a separate *Salmonella* species.

## 5 Conclusions

This work demonstrates that the removal of 252 genes from EBcgMLSTv2.0^11^ creates a more efficient and accurate scheme for analyses and characterisation of isolates across the *Salmonella* genus (SalmcgMLSTv1.0). The SalmcgMLSTv1.0 scheme has been made publicly available through pubmlst.org/salmonella. The two schemes were congruent for the clustering of isolates within their own subspecies; however, SalmcgMLSTv1.0 resolved isolates into their given subspecies grouping more quickly and identified relationships among the subspecies more precisely. The analyses undertaken here also revealed new insights into the variability of the *Salmonella* subspecies, suggesting that some had a more conserved number of loci than others. All loci within the subspecies I dataset were present in over 98% of genomes, therefore EBcgMLSTv2.0 should continue to be used for subspecies I analyses and outbreak investigations, as the inclusion of these genes will give increased resolution. Finally, it is proposed that the development of a modular system for core genome analyses will be beneficial for generating schemes that are tailored to specific requirements.

## Conflict of interest

None to declare.

The following are the supplementary data related to this article.

Supplementary Table S1 Table showing all of the isolates used within the analyses.
All of the isolates included within the analyses matched both metadata and phylogenetic clustering, with the exception of one isolate belonging to subspecies II, from PHE where further investigations still typed it as a subspecies II despite it clustering with subspecies I phylogenetically. All of these isolates were available from both Enterobase and PubMLST (at time of publication). Supplementary Table S1

Supplementary Table S2 Table showing the isolates removed from the analyses and the reasons why they were removed.
Isolates were predominantly removed due to discrepancies between their metadata and which subspecies the isolate clustered with phylogenetically. This incongruence between the two approaches meant that isolates were removed to avoid possible discrepancies in the creation of the scheme. Supplementary Table S2

Supplementary Table S3 Table showing the paralogous core genome loci.
These loci were removed from the core genome schemes in order to perform the Structure analyses, due to the presence of paralogs in one or more isolates. The schemes from which they were removed was also stated. Supplementary Table S3

Supplementary Table S4 Table showing the loci removed for each subspecies.
This table showed that many of these loci were missing in over 2% of multiple subspecies. The removal of some but not all of these loci would allow for the creation of further schemes; for example, the removal of all loci accept those specific to *S. bongori* would allow for the creation of a *S. enterica* species core scheme. Supplementary Table S4

Supplementary Table S5 Table showing the 252 loci to be removed from the EBcgMLSTv2.0.
The loci described here were absent in over 2% of at least one of the subspecies. All of the loci were present in over 98% of subspecies I, while the non-subspecies I analyses found between 10 and 108 loci needed to be removed per subspecies. 98 of these loci met the required threshold for removal (2%) in multiple subspecies. These loci were removed from EBcgMLSTv2.0 to create the genus core genome scheme proposed here. Supplementary Table S5

Supplementary Fig. S1 a and b Structure analysis using EBcgMLSTv2.0 at 17 populations (A) and Structure analysis of genus core genome scheme at 16 populations (B).
Structure [49,50] plots of 73 isolates: 7 subspecies I, II, IIIa, IIIb, IV, VI, VII, novel B, novel C and *S. bongori* and 3 novel A isolates, edited using the Distruct tool [53]. These plots were created with a burn-in of 100,000, an MCMC of 200,000 and a K of 11 and compared EBcgMLSTv2.0 with SalmcgMLSTv1.0.
A: Plot showing that at a K of 17 the Structure [49,50] algorithm is capable of identifying all the *S. enterica* subspecies and *S. bongori* when using EBcgMLSTv2.0. All of the subspecies and *S. bongori* were identified at a K of 17. At this value of K there was a significant level of admixture between Typhi and Paratyphi A isolates, which are typically highly distinct from the other subspecies I isolates, and the rest of the subspecies I isolates. The subspecies I serovar Oranienburg isolate was also very admixed with several different populations represented within the single population.
B: Plot showing that at a K of 16 the Structure [49,50] algorithm identified a very similar population structure using the SalmcgMLSTv1.0 scheme as the EBcgMLSTv2.0 scheme identified at a K of 17. Once again all of the subspecies and *S bongori* are identified and there is a high level of admixture within subspecies I. The subspecies I serovars Typhi and Paratyphi A are once again identified as highly distinct populations from the rest of the subspecies I isolates and the serovar Oranienburg isolate was again very admixed. Supplementary Fig. S1
