# Supplementary material for: A proposed core genome scheme for analyses of the Salmonella genus
Source: Genomics. 2020 Jan;112(1):371–8. doi: 10.1016/j.ygeno.2019.02.016 (PMC6978875; doi:10.1016/j.ygeno.2019.02.016)
Supplement: Supplementary Table S1 — Table showing all of the isolates used within the analyses. All of the isolates included within the analyses matched both metadata and phylogenetic clustering, with the exception of one isolate belonging to subspecies II, from PHE where further investigations still typed it as a subspecies II despite it clustering with subspecies I phylogenetically. All of these isolates were available from both Enterobase and PubMLST (at time of publication). [file mmc1.pdf]

Enterobase_Barcode	Enterobase_Name	Accession_No.	Metadata_Subspecies	Reason_removed
SAL_GA3753AA	3940-62	SRR1060612	II	Clustered_with_IIIb
SAL_BA4685AA	CFSAN006231	SRR965139	II	Clustered_with_I
SAL_BA3523AA	CFSAN024501	SRR2054210	II	Clustered_with_I
SAL_BA3526AA	CFSAN024542	SRR2054206	II	Clustered_with_I
SAL_BA3600AA	CFSAN024551	SRR2053360	II	Clustered_with_I
SAL_BA3581AA	CFSAN024557	SRR2054124	II	Clustered_with_I
SAL_BA3537AA	CFSAN024561	SRR2054195	II	Clustered_with_I
SAL_BA3576AA	CFSAN024576	SRR2054131	II	Clustered_with_I
SAL_BA5443AA	CFSAN024604	SRR2011320	II	Clustered_with_I
SAL_BA5388AA	CFSAN024614	SRR2011443	II	Clustered_with_I
SAL_DA8130AA	FAR0100	SRR1248827	II	Clustered_with_I
SAL_BA4703AA	FDA00000624	SRR2025286	II	Clustered_with_IV
SAL_GA4495AA	FDA00001404	SRR3747678	II	Clustered_with_I
SAL_FA7834AA	FDA00002751	SRR3654321	II	Clustered_with_I
SAL_BA1533AA	FDA00003428	SRR2127519	II	Clustered_with_I
SAL_CA9985AA	FDA00003681	SRR1658079	II	Clustered_with_I
SAL_EA6716AA	FDA00005031	SRR3027337	II	Clustered_with_I
SAL_DA7772AA	FMA0131	SRR1265013	II	Clustered_with_I
SAL_DA9674AA	FNW19G54	SRR1168377	II	Clustered_with_I
SAL_DA0298AA	AZ-TG74268	SRR1646584	IIIa	Clustered_with_I
SAL_DA0325AA	AZ-TG74272	SRR1646557	IIIa	Clustered_with_I
SAL_DA8127AA	FAR0092	SRR1248858	IIIa	Clustered_with_I
SAL_BA1630AA	FDA00001441	SRR2124507	IIIa	Clustered_with_IV
SAL_CA7826AA	FDA00002874	SRR1812840	IIIa	Clustered_with_I
SAL_IA0303AA	FDA00004405	SRR5182485	IIIa	Clustered_with_I
SAL_CA7208AA	FDA00008857	SRR1920226	IIIa	Clustered_with_IIIb
SAL_EA0007AA	FMA0015	SRR1139510	IIIa	Clustered_with_I
SAL_EA0031AA	FNE0029	SRR1107844	IIIa	Clustered_with_I
SAL_BA8917AA	NY_FSL_S10-1447	SRR6814029	IIIa	Clustered_with_IIIb
SAL_IA7710AA	S/20170031	traces-0qTxbwH	IIIa	Clustered_with_IIIb
SAL_JA1698AA	S/20170129V	traces-0ytsFjV	IIIa	Clustered_with_IIIb
SAL_NA8968AA	ADRDL-1249	SRR6667086	IIIb	Clustered_with_I
SAL_GA3870AA	BCW_1536	SRR1060729	IIIb	Clustered_with_I
SAL_GA3867AA	BCW_1540	SRR1060726	IIIb	Clustered_with_IV
SAL_BA5034AA	CFSAN024528	SRR2015650	IIIb	Clustered_with_I
SAL_GA7131AA	CFSAN044908	SRR4012677	IIIb	Clustered_with_I
SAL_GA7097AA	CFSAN044946	SRR4012810	IIIb	Clustered_with_I
SAL_GA7032AA	CFSAN045012	SRR4012883	IIIb	Clustered_with_I
SAL_GA7031AA	CFSAN045013	SRR4012884	IIIb	Clustered_with_I
SAL_GA7026AA	CFSAN045018	SRR4012889	IIIb	Clustered_with_I
SAL_GA7024AA	CFSAN045020	SRR4012891	IIIb	Clustered_with_I
SAL_GA7020AA	CFSAN045024	SRR4012895	IIIb	Clustered_with_I
SAL_GA7009AA	CFSAN045035	SRR4012912	IIIb	Clustered_with_I
SAL_GA7003AA	CFSAN045041	SRR4012921	IIIb	Clustered_with_I
SAL_OA2407AA	FDA00000390	SRR6805495	IIIb	Clustered_with_VI
SAL_DA9957AA	FNE0047	SRR1153373	IIIb	Clustered_with_I
SAL_JA4423AA	1256/72	traces-0VchwxH	IV	Clustered_with_I
SAL_GA3732AA	23-88	SRR1060591	IV	Clustered_with_II
SAL_GA1782AA	343-86	SRR1840598	IV	Clustered_with_IIIb
SAL_GA3863AA	BCW_1547	SRR1060722	IV	Clustered_with_I
SAL_DA0242AA	FDA00000194	SRR1648163	IV	Clustered_with_I
SAL_CA9659AA	FDA00003745	SRR1693404	IV	Clustered_with_I
SAL_CA9658AA	FDA00003746	SRR1693405	IV	Clustered_with_I
SAL_CA9683AA	FDA00003747	SRR1693289	IV	Clustered_with_I

SAL_CA9682AA	FDA00003748	SRR1693290	IV	Clustered_with_I
SAL_HA0111AA	FDA00004094	SRR4301130	IV	Clustered_with_I
SAL_MA7355AA	OH-17-7250	SRR6310076	IV	Clustered_with_I
SAL_GA3730AA	327-80	SRR1060589	S._bongori	Clustered_with_II
SAL_JA4410AA	194K	traces-0vgfZav	VI	Clustered_with_I
SAL_JA4401AA	3539/82	traces-0ZPvilA	VI	Clustered_with_I
SAL_EA0303AA	FSW0079	SRR1068316	VI	Clustered_with_I
SAL_CA2954AA	36981	SRR1967017	I	Misidentfied_subspecies_I_metadata_updated
SAL_NA5006AA	461962	traces-0WDfUBi	I	Misidentfied_subspecies_I_metadata_updated
SAL_NA5045AA	462004	traces-0aAwsuq	I	Misidentfied_subspecies_I_metadata_updated
SAL_BA2254AA	FDA00001203	SRR2086557	I	Clustered_with_II
SAL_BA2781AA	FDA00009159	SRR2075153	I	Clustered_with_II
SAL_BA5399AA	CFSAN024601	SRR2011437	I	Clustered_with_IV
SAL_CA7292AA	FDA00001035	SRR1917478	I	Enterobase_metadata_IV
SAL_DA1890AA	FDA00000118	SRR1633528	I	Clustered_with_VI
SAL_DA4281AA	MOD1_Sal_882	SRR1548438	I	Clustered_with_IV
SAL_DA5934AA	CFSAN018512	SRR1501690	I	Enterobase_metadata_II
SAL_DA7356AA	FSW0196	SRR1272845	I	Clustered_with_II
SAL_DA8117AA	FAR0083	SRR1248943	I	Enterobase_metadata_II
SAL_EA0576AA	FL_FLDACS-12104	SRR1041498	I	Enterobase_metadata_IIIb
