# Supplementary material for: A proposed core genome scheme for analyses of the Salmonella genus
Source: Genomics. 2020 Jan;112(1):371–8. doi: 10.1016/j.ygeno.2019.02.016 (PMC6978875; doi:10.1016/j.ygeno.2019.02.016)
Supplement: Supplementary Table S2 — Table showing the isolates removed from the analyses and the reasons why they were removed. Isolates were predominantly removed due to discrepancies between their metadata and which subspecies the isolate clustered with phylogenetically. This incongruence between the two approaches meant that isolates were removed to avoid possible discrepancies in the creation of the scheme. [file mmc2.pdf]

Pubmlst_Salmonella_id	Enterobase_barcode	Enterobase_Subspecies	Analysis_Subspecies	Serovar_(PubMLST)	core_genome_ST	Structure_Analysis
12318	SAL_FA4590AA	I	I	Enteritidis	1404	Yes
13447	SAL_FA5820AA	I	I	Montevideo	364	Yes
13393	SAL_CA8623AA	I	I	Oranienburg	22897	Yes
12766	SAL_BA6745AA	I	I	ParatyphiA	32363	Yes
14862	SAL_AA8860AA	I	I	Typhi	38393	Yes
13034	SAL_EA6688AA	I	I	Typhimurium	8090	Yes
13839	SAL_DA4189AA	I	I	Weslaco	18346	
12121	SAL_CA5571AA	I	I	Aba	7164	
12955	SAL_BA3628AA	I	I	Abaetetuba	34667	
13250	SAL_DA8111AA	I	I	Abaetetuba	15098	
13526	SAL_BA1356AA	I	I	Aberdeen	36477	
13441	SAL_EA2472AA	I	I	Aberdeen	11550	
14489	SAL_DA1237AA	I	I	Abony	20673	
11887	SAL_EA7944AA	I	I	Abony	6919	
14751	SAL_BA5832AA	I	I	Abortusequi	33057	
12380	SAL_BA6437AA		I	Abortusequi	32629	
12939	SAL_BA4838AA	I	I	Abortusovis	96313	
14746	SAL_BA5800AA	I	I	Abortusovis	96416	
11494	SAL_CA0889AA	I	I	Adabraka	29268	
12918	SAL_BA7386AA	I	I	Adelaide	31886	
14387	SAL_DA1414AA	I	I	Adelaide	20509	
11919	SAL_CA5962AA	I	I	Agama	25251	
11891	SAL_CA6033AA	I	I	Agama	25185	
11483	SAL_CA2960AA	I	I	Agbeni	27830	
3709	SAL_DA0837AA	I	I	Agbeni	21045	
11791	SAL_CA3654AA	I	I	Ago	7548	
3592	SAL_DA1427AA	I	I	Ago	20496	
11800	SAL_CA5995AA	I	I	Agona	25221	
13675	SAL_DA1540AA	I	I	Agona	20397	
12418	SAL_BA6385AA		I	Agoueve	32673	
12004	SAL_CA0040AA	I	I	Ajiobo	29961	
12303	SAL_FA4646AA	I	I	Ajiobo	1350	
13490	SAL_EA3211AA		I	Alachua	10942	
12255	SAL_EA8109AA	I	I	Alachua	6758	
14148	SAL_DA8590AA	I	I	Albany	14674	
13146	SAL_EA1884AA		I	Albany	12026	
3605	SAL_DA1399AA	I	I	Altona	20524	
11608	SAL_EA7434AA	I	I	Altona	7412	
13637	SAL_EA2421AA	I	I	Amager	11588	
11997	SAL_CA2720AA	I	I	Amherstiana	28027	
14864	SAL_DA6119AA		I	Amoutive	16759	
14114	SAL_DA9345AA	I	I	Amsterdam	14007	
13670	SAL_DA2493AA	I	I	Anatum	19618	
14276	SAL_FA4751AA	I	I	Anatum	1258	
3617	SAL_DA1382AA	I	I	Anecho	20541	
13547	SAL_BA1304AA	I	I	Anfo	36520	
3673	SAL_DA1149AA	I	I	Apapa	20757	
14254	SAL_EA3954AA	I	I	Apapa	10314	
14177	SAL_DA9653AA	I	I	Apeyeme	13739	
13650	SAL_DA0096AA	I	I	Astridplein	21695	
13468	SAL_BA2680AA	I	I	Augustenborg	35403	
3684	SAL_DA1067AA	I	I	Augustenborg	20836	
13501	SAL_FA0525AA	I	I	Bahrenfeld	4844	
13099	SAL_EA5427AA	I	I	Baildon	9067	
12359	SAL_FA4577AA	I	I	Bakau	1416	
13987	SAL_DA5102AA	I	I	Ball	17628	
14865	SAL_DA5818AA		I	Ball	96315	
15328	SAL_DA0255AA	I	I	Banana	21574	
14093	SAL_DA2348AA	I	I	Bangkok	96359	
13509	SAL_EA2864AA	I	I	Bangkok	96411	
11469	SAL_BA6634AA		I	Bardo	32461	
11794	SAL_CA6165AA	I	I	Bardo	25070	
15161	SAL_BA6617AA		I	Bareilly	32474	
11670	SAL_CA2357AA	I	I	Bareilly	28345	
11474	SAL_BA6424AA		I	Beaudesert	32640	

13752	SAL_BA6541AA	I	I	Benue	32532
14153	SAL_DA6205AA	I	I	Berta	16681
14925	SAL_DA9936AA	I	I	Berta	6160
11484	SAL_BA9469AA	I	I	Bispebjerg	30407
11839	SAL_CA1431AA	I	I	Bispebjerg	29006
13234	SAL_EA0465AA	I	I	Blijdorp	13021
14460	SAL_DA1137AA	I	I	Blockley	20768
14866	SAL_DA6083AA		I	Blockley	16793
11481	SAL_BA9159AA	I	I	Bochum	30668
12371	SAL_BA6450AA		I	Bonariensis	32618
11584	SAL_EA8022AA	I	I	Bonariensis	6844
12015	SAL_CA3148AA	I	I	Bonn	27667
12130	SAL_CA5741AA	I	I	Bonn	25438
11642	SAL_CA4674AA	I	I	Bournemouth	26330
13999	SAL_DA6667AA	I	I	Bovismorbificans	16291
12232	SAL_EA7676AA	I	I	Bovismorbificans	7176
12218	SAL_CA3539AA	I	I	Braenderup	27326
15326	SAL_DA2008AA	I	I	Braenderup	20019
11633	SAL_CA2420AA	I	I	Brancaster	28292
14504	SAL_DA0374AA	I	I	Brandenburg	21473
11607	SAL_EA7938AA	I	I	Brandenburg	6925
3586	SAL_DA1435AA	I	I	Brazzaville	20488
14368	SAL_DA0889AA	I	I	Bredeney	20996
13832	SAL_EA0537AA	I	I	Bredeney	12958
11473	SAL_EA7205AA	I	I	Bron	7622
13640	SAL_DA1634AA	I	I	Brunei	20326
11851	SAL_EA9164AA	I	I	Brunei	5901
13667	SAL_DA2558AA	I	I	Butantan	19572
12283	SAL_EA7969AA	I	I	Butantan	6895
12925	SAL_DA6861AA	I	I	Buzu	16135
13517	SAL_BA0946AA	I	I	Cannstatt	34447
3689	SAL_DA1037AA	I	I	Caracas	20864
14509	SAL_DA1332AA	I	I	Caracas	20585
3635	SAL_DA1361AA	I	I	Carmel	20561
3757	SAL_DA0398AA	I	I	Carno	21452
12952	SAL_BA3517AA	I	I	Carrau	34748
13627	SAL_DA2387AA	I	I	Carrau	19700
14910	SAL_EA0381AA	I	I	Cerro	13096
14223	SAL_EA6587AA	I	I	Cerro	8156
13625	SAL_CA7241AA	I	I	Champaign	24095
11721	SAL_CA1566AA	I	I	Chandans	28896
14867	SAL_DA6110AA		I	Chandans	16768
3723	SAL_DA0708AA	I	I	Charity	21166
14463	SAL_DA1071AA	I	I	Charity	20832
12969	SAL_BA5433AA	I	I	Chester	33401
11471	SAL_EA7727AA	I	I	Chester	7127
14200	SAL_CA7676AA	I	I	Chicago	23715
14039	SAL_DA6322AA	I	I	Chicago	16577
13553	SAL_EA9898AA	I	I	Chincol	5391
11882	SAL_CA6008AA	I	I	Chingola	25210
14697	SAL_BA9932AA	I	I	Choleraesuis	30048
16116	SAL_OA2109AA	I	I	Choleraesuis	1273
12214	SAL_CA4572AA	I	I	Claibornei	26418
13555	SAL_FA0518AA	I	I	Claibornei	4851
11897	SAL_CA6466AA	I	I	Coeln	24800
3587	SAL_DA1433AA	I	I	Coeln	20490
13996	SAL_DA6889AA	I	I	Coleypark	16108
12235	SAL_CA0159AA	I	I	Colindale	29864
14074	SAL_DA1623AA	I	I	Colindale	20336
13580	SAL_BA5473AA	I	I	Colobane	33375
14386	SAL_DA1330AA	I	I	Concord	20587
12262	SAL_CA3268AA	I	I	Corvallis	27561
12354	SAL_FA4332AA	I	I	Corvallis	1643
3735	SAL_DA0624AA	I	I	Cotham	21246
3664	SAL_DA1218AA	I	I	Cotham	20691
11874	SAL_CA2896AA	I	I	Cremieu	1831

13581	SAL_CA8455AA	I	I	Cubana	23035
13550	SAL_EA6461AA	I	I	Cubana	8253
3756	SAL_DA0402AA	I	I	Curacao	21449
14471	SAL_DA0431AA	I	I	Curacao	21422
13507	SAL_EA4270AA	I	I	Derby	10059
11582	SAL_EA8093AA	I	I	Derby	6774
11888	SAL_CA6176AA	I	I	Deversoir	25060
12306	SAL_FA4153AA	I	I	Djugu	1807
14052	SAL_DA6294AA	I	I	Dortmund	16603
14750	SAL_BA5884AA	I	I	Dublin	33007
13129	SAL_EA3628AA	I	I	Dublin	10618
14297	SAL_EA0235AA	I	I	Duesseldorf	13223
11617	SAL_CA4671AA	I	I	Duisburg	26331
11777	SAL_CA6772AA	I	I	Duisburg	24516
12131	SAL_CA5591AA	I	I	Durban	25568
14439	SAL_DA1371AA	I	I	Durham	20552
11604	SAL_EA7605AA	I	I	Durham	7244
3588	SAL_DA1432AA	I	I	Ealing	20491
13974	SAL_DA9340AA	I	I	Ealing	14011
14527	SAL_DA0649AA	I	I	Eastbourne	21222
12280	SAL_EA7776AA	I	I	Eastbourne	7079
11730	SAL_CA1200AA	I	I	Ede	29128
13576	SAL_BA4708AA	I	I	Edinburg	33948
3623	SAL_DA1375AA	I	I	Edinburg	20548
14484	SAL_DA1324AA	I	I	Eimsbuettel	20593
3654	SAL_DA1325AA	I	I	Eimsbuettel	20592
14209	SAL_EA1478AA	I	I	Eko	96319
12669	SAL_FA5655AA	I	I	Eko	504
11916	SAL_CA6366AA	I	I	Elisabethville	24890
12259	SAL_CA4185AA	I	I	Emek	26748
12081	SAL_CA5172AA	I	I	Emek	25915
14977	SAL_BA1109AA		I	Enteritidis	36679
11786	SAL_CA0842AA	I	I	Eschberg	29303
13277	SAL_EA0593AA	I	I	Essen	12914
14239	SAL_EA5355AA	I	I	Falkensee	9111
13092	SAL_FA5326AA	I	I	Falkensee	782
11852	SAL_CA1597AA	I	I	Fanti	28867
13689	SAL_DA5599AA	I	I	Farmsen	17192
12970	SAL_BA5428AA	I	I	Finkenwerder	33405
14302	SAL_DA8020AA	I	I	Fischerstrasse	15181
13530	SAL_CA8637AA	I	I	Florida	22883
13426	SAL_EA4947AA	I	I	Florida	9463
12201	SAL_BA9683AA	I	I	Fluntern	30238
14355	SAL_DA0947AA	I	I	Fluntern	20948
14198	SAL_DA5125AA	I	I	Freetown	17608
13442	SAL_EA2471AA	I	I	Freetown	11551
13515	SAL_EA2716AA	I	I	Freiburg	11356
12921	SAL_BA6893AA	I	I	Fresno	32226
13383	SAL_CA9185AA	I	I	Fresno	22421
13105	SAL_FA4932AA		I	Fulda	1111
11658	SAL_CA3847AA	I	I	Fulica	27047
3632	SAL_DA1364AA	I	I	Gafsa	20558
11828	SAL_CA5919AA	I	I	Gaillac	25288
12120	SAL_CA4977AA	I	I	Galiema	26069
14748	SAL_BA5777AA	I	I	Gallinarum	96340
14775	SAL_BA5850AA	I	I	Gallinarum	33040
13458	SAL_FA0813AA	I	I	Gamaba	4612
11932	SAL_CA6421AA	I	I	Gaminara	24841
14887	SAL_EA5524AA		I	Gaminara	9015
11553	SAL_BA8689AA	I	I	Gateshead	31041
11646	SAL_CA0576AA	I	I	Gatow	29526
12289	SAL_FA4187AA	I	I	Gdansk	1775
13241	SAL_DA9597AA	I	I	Give	13788
14260	SAL_EA6578AA	I	I	Give	8165
11487	SAL_BA9507AA	I	I	Glostrup	30377
11615	SAL_CA2163AA	I	I	Glostrup	28515

14358	SAL_DA0639AA	I	I	Goelzau	21232
11663	SAL_BA9642AA	I	I	Goldcoast	30265
11665	SAL_CA3826AA	I	I	Goldcoast	7224
11761	SAL_CA4212AA	I	I	Gombe	26724
13466	SAL_CA7894AA	I	I	Goulfey	23523
11861	SAL_CA3999AA	I	I	Goverdhan	26910
3638	SAL_DA1357AA	I	I	Goverdhan	20564
12186	SAL_CA3187AA	I	I	Grancanaria	27633
11707	SAL_CA1890AA	I	I	Grumpensis	28674
14094	SAL_DA2437AA	I	I	Grumpensis	19662
16179	SAL_CA1193AA	I	I	Hadar	29135
15355	SAL_DA5106AA	I	I	Hadar	17624
14181	SAL_DA9664AA	I	I	Haduna	13729
12003	SAL_CA3911AA	I	I	Haifa	26988
11820	SAL_CA6620AA	I	I	Haifa	24658
11651	SAL_CA3023AA	I	I	Harrisonburg	27772
12894	SAL_BA5155AA	I	I	Hartford	33598
14143	SAL_DA8572AA	I	I	Hartford	14691
11856	SAL_CA5991AA	I	I	Havana	25224
11884	SAL_EA7159AA	I	I	Havana	7668
15357	SAL_DA5434AA	I	I	Heidelberg	17330
14931	SAL_DA5454AA	I	I	Heidelberg	17318
14003	SAL_DA6639AA	I	I	Hermannswerder	16315
3663	SAL_DA1223AA	I	I	Herston	20687
13409	SAL_BA1920AA	I	I	Hindmarsh	36023
14000	SAL_DA6646AA	I	I	Hofit	16309
14916	SAL_BA8707AA	I	I	Holcomb	31026
14514	SAL_DA1525AA	I	I	Hull	20410
12293	SAL_FA4723AA	I	I	Hull	1285
14085	SAL_DA6577AA	I	I	Huvudsta	16369
14737	SAL_BA5802AA	I	I	Hvittingfoss	33085
14364	SAL_DA0860AA	I	I	Hvittingfoss	21023
11974	SAL_CA3671AA	I	I	Ibadan	27205
11987	SAL_CA6337AA	I	I	Ibadan	24915
11775	SAL_CA6843AA	I	I	Indiana	24450
14872	SAL_DA5828AA		I	Indiana	17005
11691	SAL_CA3032AA	I	I	Infantis	27764
12916	SAL_CA7360AA	I	I	Infantis	23987
3646	SAL_DA1337AA	I	I	Inganda	20582
13248	SAL_DA8118AA	I	I	Inganda	96366
13891	SAL_DA8473AA		I	Inverness	14780
14032	SAL_DA9130AA	I	I	Ipswich	14212
12051	SAL_CA3222AA	I	I	Irumu	27602
3656	SAL_DA1305AA	I	I	Irumu	20609
3613	SAL_DA1389AA	I	I	Isangi	20534
13984	SAL_DA7825AA	I	I	Isangi	15366
15167	SAL_BA6598AA		I	Istanbul	32491
11698	SAL_CA3473AA	I	I	Ituri	27386
12329	SAL_FA4671AA	I	I	Ituri	1328
11676	SAL_CA2955AA	I	I	Jangwani	27835
11548	SAL_CA4323AA	I	I	Java	26629
12129	SAL_CA5609AA	I	I	Java	25552
13495	SAL_DA5257AA	I	I	Javiana	17497
13879	SAL_EA2394AA		I	Javiana	96412
12189	SAL_BA9477AA	I	I	Jedburgh	30400
12342	SAL_FA4624AA	I	I	Joal	1371
13705	SAL_BA1941AA	I	I	Johannesburg	36005
13877	SAL_EA1394AA		I	Johannesburg	12400
13395	SAL_FA0513AA	I	I	Jukestown	96505
11909	SAL_CA2818AA	I	I	Kalina	27944
11872	SAL_EA7436AA	I	I	Kambole	7410
11889	SAL_CA6732AA	I	I	Kedougou	24553
13399	SAL_CA8425AA	I	I	Kedougou	23058
12088	SAL_CA4883AA	I	I	Kentucky	26144
14347	SAL_DA0934AA	I	I	Kentucky	20957
14488	SAL_DA0495AA	I	I	Kenya	21363

12104	SAL_CA5831AA	I	I	Kiambu	25360
3686	SAL_DA1050AA	I	I	Kiambu	20852
13742	SAL_CA7796AA	I	I	Kibusi	23610
14532	SAL_DA1303AA	I	I	Kibusi	20611
14078	SAL_DA7167AA	I	I	Kingabwa	15867
13451	SAL_FA5850AA	I	I	Kingabwa	336
11525	SAL_CA1079AA	I	I	Kingston	29219
3650	SAL_DA1331AA	I	I	Kingston	20586
11809	SAL_CA6591AA	I	I	Kintambo	24684
14566	SAL_DA7210AA	I	I	Kintambo	15826
11962	SAL_CA2355AA	I	I	Kirkee	28347
12610	SAL_EA4567AA		I	Kisangani	9769
3639	SAL_DA1356AA	I	I	Kisarawe	20565
14818	SAL_DA4093AA	I	I	Kisarawe	18429
11986	SAL_CA6010AA	I	I	Kottbus	25208
3710	SAL_DA0836AA	I	I	Kottbus	20739
12134	SAL_CA4914AA	I	I	Krefeld	26089
13479	SAL_BA1657AA	I	I	Kua	36239
3603	SAL_DA1404AA	I	I	Kua	20519
13411	SAL_CA7286AA	I	I	Kumasi	24054
14805	SAL_DA8797AA	I	I	Labadi	14482
12070	SAL_CA5812AA	I	I	Lagos	25376
3648	SAL_DA1334AA	I	I	Lagos	20584
11751	SAL_BA8548AA	I	I	Langensalza	31163
13237	SAL_DA9989AA	I	I	Langford	13437
14047	SAL_DA6086AA	I	I	Lanka	16790
14298	SAL_DA8055AA	I	I	Lansing	15151
12054	SAL_BA8080AA	I	I	Larochelle	31482
14183	SAL_DA9900AA	I	I	Larochelle	13510
12108	SAL_CA5281AA	I	I	Lattenkamp	96380
12044	SAL_CA5852AA	I	I	Lerum	25343
12058	SAL_CA1439AA	I	I	Lexington	29000
14115	SAL_DA9338AA	I	I	Lexington	14013
13329	SAL_CA7124AA	I	I	Lille	24205
12230	SAL_CA4826AA	I	I	Limete	26193
12265	SAL_EA7553AA	I	I	Lindern	7296
14035	SAL_DA9007AA	I	I	Lingwala	14315
14028	SAL_DA9796AA	I	I	Lingwala	13605
3743	SAL_DA0540AA	I	I	Litchfield	21322
14890	SAL_EA5515AA		I	Litchfield	9024
14056	SAL_DA3958AA	I	I	Liverpool	18544
13247	SAL_DA8129AA	I	I	Liverpool	15080
12148	SAL_CA3340AA	I	I	Livingstone	27497
14797	SAL_DA7376AA	I	I	Livingstone	15696
11708	SAL_CA4202AA	I	I	Lome	26734
12111	SAL_CA5217AA	I	I	Lomita	25880
12183	SAL_CA3517AA	I	I	London	27346
13508	SAL_EA2863AA	I	I	London	11229
12317	SAL_FA4621AA	I	I	Los-Angeles	1374
13284	SAL_EA0310AA	I	I	Louga	13159
12292	SAL_FA3563AA	I	I	Louisiana	2320
13835	SAL_DA7298AA	I	I	Luciana	15767
12383	SAL_BA6636AA		I	Luckenwalde	32459
13483	SAL_BA1635AA	I	I	Luke	36258
14874	SAL_DA4310AA		I	Luke	18240
14792	SAL_EA1314AA	I	I	Maastricht	12468
14875	SAL_DA4289AA		I	Madelia	18256
13425	SAL_EA4948AA	I	I	Madelia	9462
11910	SAL_CA3592AA	I	I	Madras	27275
13612	SAL_CA7883AA	I	I	Magwa	23518
13340	SAL_CA9712AA	I	I	Malstatt	22005
11576	SAL_EA7398AA	I	I	Malstatt	7445
11599	SAL_CA3076AA	I	I	Manchester	27723
12256	SAL_EA7881AA	I	I	Manchester	6978
12276	SAL_CA0026AA	I	I	Manhattan	29974
14455	SAL_DA1335AA	I	I	Manhattan	20583

12005	SAL_CA0858AA	I	I	Marseille	29294	
13748	SAL_BA7026AA	I	I	Martonos	32119	
13289	SAL_DA7014AA	I	I	Martonos	15998	
14108	SAL_DA9627AA	I	I	Matadi	13763	
13233	SAL_EA0486AA	I	I	Mathura	13003	
11600	SAL_CA4342AA	I	I	Matopeni	26613	
13639	SAL_DA1653AA	I	I	Matopeni	20309	
11797	SAL_BA9242AA	I	I	Mbandaka	30599	
11892	SAL_CA3573AA	I	I	Mbandaka	27294	Yes
13989	SAL_DA7234AA	I	I	Meleagridis	15804	
13988	SAL_DA7238AA	I	I	Meleagridis	15801	
13572	SAL_BA3402AA	I	I	Mgulani	34834	
14025	SAL_DA9758AA	I	I	Mgulani	13643	
13360	SAL_BA1431AA	I	I	Miami	36423	
13571	SAL_BA5522AA	I	I	Miami	33332	
14090	SAL_DA2593AA	I	I	Michigan	19545	
14086	SAL_DA6517AA	I	I	Michigan	16416	
12072	SAL_CA5666AA	I	I	Mikawasima	25503	
13694	SAL_DA3823AA	I	I	Mikawasima	18659	
13562	SAL_CA8566AA	I	I	Milwaukee	22942	
14125	SAL_DA0338AA	I	I	Minnesota	21502	
12816	SAL_DA5425AA	I	I	Minnesota	17339	
14980	SAL_CA7550AA	I	I	Mishmarhaemek	23813	
11496	SAL_CA1577AA	I	I	Mississippi	28886	
12046	SAL_CA2262AA	I	I	Mississippi	28425	
12178	SAL_CA1055AA	I	I	Molade	29228	
14145	SAL_DA8534AA	I	I	Molade	14727	
11831	SAL_CA3147AA	I	I	Monschaui	27668	
14496	SAL_DA1225AA	I	I	Monschaui	20685	
15114	SAL_DA2354AA	I	I	Montevideo	19729	
14754	SAL_EA0855AA	I	I	Moscow	12697	
12267	SAL_EA6885AA	I	I	Mount-Pleasant	7924	
14567	SAL_DA7201AA	I	I	Muenchen	96317	
12159	SAL_EA7122AA	I	I	Muenchen	7703	
14481	SAL_DA1770AA	I	I	Muenster	20214	
12772	SAL_EA8407AA		I	Muenster	6519	
13676	SAL_DA1536AA	I	I	Nagoya	20399	
14491	SAL_DA1500AA	I	I	Napoli	20434	
3564	SAL_DA1527AA	I	I	Napoli	20408	
11727	SAL_BA9919AA	I	I	Nchanga	30059	
14147	SAL_DA8598AA	I	I	Nchanga	14667	
12370	SAL_BA6409AA		I	Nessziona	32652	
13598	SAL_CA8237AA	I	I	Nessziona	23219	
12109	SAL_CA5351AA	I	I	Neukoelln	25769	
14771	SAL_BA5816AA	I	I	Newport	33071	
12504	SAL_CA0162AA	I	I	Newport	29861	
14899	SAL_CA0183AA	I	I	Newport	29819	
15371	SAL_DA1988AA	I	I	Newport	20035	
12982	SAL_CA8511AA	I	I	Nima	9652	
14838	SAL_EA3963AA	I	I	Nima	10307	
15329	SAL_DA0236AA	I	I	Norwich	21593	
13888	SAL_DA8629AA		I	Norwich	14638	
12638	SAL_BA1171AA	I	I	Nottingham	36629	
13262	SAL_DA7503AA	I	I	Nottingham	15594	
14080	SAL_DA7145AA	I	I	Oakland	15887	
11878	SAL_CA6443AA	I	I	Obogu	24820	
11740	SAL_BA9383AA	I	I	Offa	30478	
14953	SAL_BA4813AA	I	I	Ohio	33879	
15318	SAL_DA9367AA	I	I	Ohio	13986	
11690	SAL_BA8413AA	I	I	Onireke	31268	
16074	SAL_JA7775AA	I	I	Oranienburg	96745	
12086	SAL_CA4891AA	I	I	Orientalis	26139	
14159	SAL_DA6259AA	I	I	Orientalis	16632	
13480	SAL_BA1680AA	I	I	Orion	36217	
13337	SAL_EA2841AA	I	I	Orion	11248	
11556	SAL_CA3178AA	I	I	Oslo	27641	

14168	SAL_EA0037AA	I	I	Oslo	13404
12802	SAL_EA0651AA	I	I	Othmarschen	12864
13346	SAL_EA4852AA	I	I	Othmarschen	9548
13516	SAL_BA0813AA	I	I	Ouakam	36860
12373	SAL_BA6405AA		I	Overschie	32656
11666	SAL_BA9212AA	I	I	Panama	30623
12297	SAL_FA4726AA	I	I	Panama	1282
12113	SAL_CA4933AA	I	I	ParatyphiA	26104
15215	SAL_AA9577AA	I	I	ParatyphiB	37820
11503	SAL_EA7707AA	I	I	ParatyphiB	7147
13207	SAL_DA4538AA	I	I	ParatyphiC	18065
15195	SAL_EA9779AA	I	I	ParatyphiC	5463
12124	SAL_CA5541AA	I	I	Plymouth	25610
13804	SAL_FA2243AA		I	Plymouth	3413
14876	SAL_DA4321AA		I	Poano	18229
3685	SAL_DA1053AA	I	I	Pomona	20849
13684	SAL_EA3242AA	I	I	Pomona	9292
11557	SAL_CA2649AA	I	I	Poona	28091
14175	SAL_EA0432AA	I	I	Poona	13049
13514	SAL_BA2581AA	I	I	Portland	35486
12045	SAL_CA5844AA	I	I	Potsdam	141
14101	SAL_DA9808AA	I	I	Potsdam	13593
12940	SAL_BA4832AA	I	I	Pullorum	96339
15171	SAL_BA5634AA	I	I	Pullorum	33235
14426	SAL_DA1407AA	I	I	Ramatgan	20516
14458	SAL_DA1410AA	I	I	Ramatgan	20513
15378	SAL_BA5586AA	I	I	Reading	33276
14238	SAL_EA6596AA	I	I	Reading	8147
12254	SAL_EA7785AA	I	I	Remo	7071
14129	SAL_CA9769AA	I	I	Richmond	21962
13330	SAL_EA6520AA	I	I	Richmond	8204
3619	SAL_DA1379AA	I	I	Ridge	20544
12164	SAL_EA7292AA	I	I	Ridge	7541
13025	SAL_BA3548AA	I	I	Rissen	34689
13573	SAL_CA7886AA	I	I	Rissen	23363
13274	SAL_DA6360AA	I	I	Rubislaw	16542
13889	SAL_DA8619AA		I	Rubislaw	14647
14057	SAL_DA3957AA	I	I	Ruiru	18545
12824	SAL_DA2372AA	I	I	Saintpaul	19714
13160	SAL_FA3029AA		I	Saintpaul	2697
13389	SAL_CA8418AA	I	I	Salford	23064
13511	SAL_EA2871AA	I	I	Salford	11224
13454	SAL_BA2154AA	I	I	Sandiego	35823
13351	SAL_DA2155AA	I	I	Sandiego	19897
12014	SAL_CA5707AA	I	I	Sangalkam	25466
13831	SAL_EA0243AA	I	I	Sanjuan	13215
14918	SAL_BA3037AA	I	I	Saphra	35121
11749	SAL_CA4150AA	I	I	Saphra	26781
13836	SAL_DA4431AA	I	I	Saugus	18149
14295	SAL_EA0318AA	I	I	Schleissheim	13151
12083	SAL_CA5763AA	I	I	Schwarzengrund	25420
13170	SAL_EA2959AA	I	I	Schwarzengrund	11155
11581	SAL_EA8060AA	I	I	Sekondi	6807
14176	SAL_EA0416AA	I	I	Sendai	13064
14766	SAL_EA1141AA	I	I	Sendai	12563
15346	SAL_BA9257AA	I	I	Senegal	30588
14109	SAL_DA9423AA	I	I	Senegal	13936
14952	SAL_BA6295AA	I	I	Senftenberg	32746
12294	SAL_FA4448AA	I	I	Senftenberg	1537
12984	SAL_CA7823AA	I	I	Shamba	23585
11522	SAL_CA0483AA	I	I	Shubra	96341
12313	SAL_FA4260AA	I	I	Shubra	1706
13428	SAL_BA5909AA	I	I	Simi	32992
12140	SAL_CA5076AA	I	I	Simi	25988
12075	SAL_CA5031AA	I	I	Singapore	26026
3575	SAL_DA1501AA	I	I	Singapore	20433

14215	SAL_EA1443AA	I	I	Soerenga	12366
12160	SAL_EA7097AA	I	I	Soerenga	7726
13806	SAL_FA2241AA		I	Solt	3415
12016	SAL_CA0532AA	I	I	Soumbedioune	29564
11505	SAL_CA1980AA	I	I	Stanley	28635
12123	SAL_CA5438AA	I	I	Stanley	25695
14490	SAL_DA0614AA	I	I	Stanleyville	21255
14352	SAL_DA1530AA	I	I	Stanleyville	20405
13805	SAL_FA2242AA		I	Stockholm	3414
13437	SAL_BA6141AA	I	I	Stormont	32870
11979	SAL_BA8568AA	I	I	Stourbridge	31149
14582	SAL_DA5972AA	I	I	Suelldorf	16888
13756	SAL_BA5449AA	I	I	Sundsvall	33385
12374	SAL_BA6448AA		I	Takoradi	32620
12257	SAL_EA7756AA	I	I	Takoradi	7098
13394	SAL_CA8565AA	I	I	Tallahassee	22943
14293	SAL_EA0305AA	I	I	Tallahassee	13164
11865	SAL_CA3213AA	I	I	Tamale	27610
12219	SAL_CA1809AA	I	I	Tamberma	28709
13702	SAL_DA5464AA	I	I	Tamberma	17308
13475	SAL_BA2663AA	I	I	Tananarive	35419
13687	SAL_DA3864AA	I	I	Tarshyne	18625
13810	SAL_FA5191AA		I	Teddington	894
12095	SAL_CA2530AA	I	I	Tees	28197
11620	SAL_CA3314AA	I	I	Tees	27520
14149	SAL_DA8531AA	I	I	Teko	14684
13467	SAL_EA3221AA		I	Teko	10932
12911	SAL_CA7801AA	I	I	Telaviv	23605
12912	SAL_CA7807AA	I	I	Telaviv	23600
13322	SAL_DA1481AA	I	I	Telelkebir	20452
14839	SAL_EA5183AA	I	I	Telelkebir	9256
13537	SAL_BA1369AA	I	I	Telhashomer	36465
14188	SAL_DA5275AA	I	I	Telhashomer	17480
12176	SAL_CA1241AA	I	I	Tennessee	29093
12896	SAL_DA9493AA	I	I	Tennessee	13876
13368	SAL_BA1214AA	I	I	Texas	36589
16199	SAL_CA6461AA	I	I	Thompson	24805
13310	SAL_FA7274AA	I	I	Thompson	40214
11925	SAL_CA3065AA	I	I	Toronto	27733
13402	SAL_BA1952AA	I	I	Trachau	35996
14794	SAL_DA7257AA	I	I	Treforest	15785
14881	SAL_DA4280AA		I	Troy	18264
11900	SAL_CA1189AA	I	I	Tshiongwe	29138
13566	SAL_BA1923AA	I	I	Tucson	36022
14803	SAL_DA8805AA	I	I	Tucson	14488
11735	SAL_CA0625AA	I	I	Tudu	29490
13058	SAL_FA6947AA	I	I	Typhi	40645
11457	SAL_BA6456AA	I	I	Typhimurium	32612
12642	SAL_DA4517AA	I	I	Typhisuis	18095
14773	SAL_EA1137AA	I	I	Typhisuis	12567
13649	SAL_DA0077AA	I	I	Uganda	21711
14796	SAL_DA7418AA	I	I	Uganda	15661
3608	SAL_DA1396AA	I	I	Umbilo	20527
12381	SAL_BA6446AA		I	Urbana	32622
15364	SAL_DA3281AA	I	I	Urbana	19067
15165	SAL_BA6605AA		I	Uzaramo	32486
13097	SAL_EA9865AA		I	Uzaramo	5417
3644	SAL_DA1342AA	I	I	Vejle	20578
3636	SAL_DA1360AA	I	I	Vejle	20555
13858	SAL_EA0580AA	I	I	Veneziana	12926
14882	SAL_DA4298AA		I	Vinohrady	18249
16196	SAL_CA5562AA	I	I	Virchow	25226
16258	SAL_IA1864AA	I	I	Virchow	69865
11758	SAL_CA3961AA	I	I	Virginia	26946
3585	SAL_DA1436AA	I	I	Vitkin	20487
11593	SAL_EA7502AA	I	I	Vitkin	7346

11543	SAL_CA0584AA	I	I	Volkmarsdorf	29523	
14883	SAL_DA4303AA		I	Wandsworth	18245	
14178	SAL_DA9656AA	I	I	Wandsworth	13736	
14404	SAL_DA0410AA	I	I	Wangata	21442	
12324	SAL_FA4457AA	I	I	Wangata	1528	
13320	SAL_DA1482AA	I	I	Warragul	20451	
12368	SAL_BA6417AA		I	Waycross	32646	
14048	SAL_DA6288AA	I	I	Waycross	16607	
14040	SAL_DA6331AA	I	I	Welikade	16568	
14013	SAL_EA0221AA	I	I	Welikade	13235	
12031	SAL_CA0628AA	I	I	Weltevreden	29487	
14024	SAL_DA9792AA	I	I	Weltevreden	13609	
13577	SAL_BA4853AA	I	I	Wentworth	33850	
13890	SAL_DA8617AA		I	Weslaco	14648	
11862	SAL_BA9193AA	I	I	Westhampton	30640	
12057	SAL_CA1276AA	I	I	Westhampton	29071	
14098	SAL_EA0003AA	I	I	Westminster	13426	
15127	SAL_EA5413AA		I	Weybridge	9078	
12278	SAL_CA4535AA	I	I	Wien	26445	
11590	SAL_EA7361AA	I	I	Wilhelmsburg	7478	
14884	SAL_DA4274AA		I	Woodinville	18269	
12133	SAL_CA5520AA	I	I	Worb	25627	
12417	SAL_BA6387AA		I	Worthington	32671	
13142	SAL_EA1958AA		I	Worthington	11962	
13635	SAL_BA1534AA	I	I	Yenne	36343	
12425	SAL_BA6639AA	I	I	Yoruba	32456	
13502	SAL_CA7117AA	I	I	Yoruba	24212	
11939	SAL_CA2386AA	I	I	Zaiman	25776	
13452	SAL_BA2157AA	I	I	Zanzibar	35820	
3708	SAL_DA0849AA	I	I	Zanzibar	21034	
16453	SAL_CA2196AA	II	II	-	28486	Yes
16504	SAL_DA4562AA	II	II	-	18046	Yes
12625	SAL_GA1789AA	II	II	-	43907	Yes
16341	SAL_MA5896AA	II	II	-	97089	
16000	SAL_MA5912AA	II	II	-	97100	Yes
17101	SAL_MA5944AA	II	II	-	97158	Yes
16350	SAL_MA5981AA	II	II	-	97186	Yes
14710	SAL_BA0023AA	II	II	-	37425	
16591	SAL_BA0792AA	II	II	-	36868	
12471	SAL_BA1522AA	II	II	-	36352	
13407	SAL_BA1949AA	II	II	-	35999	Yes
13559	SAL_BA1991AA	II	II	-	35912	
13558	SAL_BA1995AA	II	II	-	35912	
13560	SAL_BA2051AA	II	II	-	35912	
13455	SAL_BA2147AA	II	II	-	35828	
13764	SAL_BA2476AA	II	II	-	35563	
13766	SAL_BA2715AA	II	II	-	35372	
16426	SAL_BA2848AA	II	II	-	35265	
13384	SAL_BA6579AA	II	II	-	32508	
16168	SAL_BA7502AA	II	II	-	31799	
16169	SAL_BA7507AA	II	II	-	31795	
13853	SAL_BA8274AA	II	II	-	31365	
13852	SAL_BA8276AA	II	II	-	31363	
16444	SAL_BA9415AA	II	II	-	30455	
16423	SAL_BA9837AA	II	II	-	30123	
16424	SAL_BA9883AA	II	II	-	30087	
11943	SAL_CA0156AA	II	II	-	29867	
12391	SAL_CA1576AA	II	II	-	28887	
11942	SAL_CA1974AA	II	II	-	28638	
11755	SAL_CA2476AA	II	II	-	28241	
16457	SAL_CA2496AA	II	II	-	28227	
16412	SAL_CA2832AA	II	II	-	27932	
11684	SAL_CA3352AA	II	II	-	27486	
12195	SAL_CA3582AA	II	II	-	27285	
16469	SAL_CA3703AA	II	II	-	27174	
11499	SAL_CA3869AA	II	II	-	27027	

11552	SAL_CA3870AA	II	II	-	27026
12047	SAL_CA4049AA	II	II	-	26865
11637	SAL_CA4083AA	II	II	-	26839
16414	SAL_CA4136AA	II	II	-	26793
16476	SAL_CA4521AA	II	II	-	26459
11798	SAL_CA4655AA	II	II	-	26344
13586	SAL_CA4878AA	II	II	-	26147
12119	SAL_CA5044AA	II	II	-	26014
16486	SAL_CA5746AA	II	II	-	25433
16489	SAL_CA5916AA	II	II	-	25289
16490	SAL_CA5990AA	II	II	-	25225
13419	SAL_CA7284AA	II	II	-	96382
13413	SAL_CA7301AA	II	II	-	24041
14473	SAL_DA0524AA	II	II	-	21337
14371	SAL_DA0772AA	II	II	-	21106
14411	SAL_DA1039AA	II	II	-	20862
14379	SAL_DA1380AA	II	II	-	20543
14351	SAL_DA1408AA	II	II	-	20515
14333	SAL_DA1420AA	II	II	-	20503
13308	SAL_DA2030AA	II	II	-	20001
14062	SAL_DA3488AA	II	II	-	18908
14825	SAL_DA3552AA	II	II	-	18863
16497	SAL_DA4132AA	II	II	-	18398
14194	SAL_DA4372AA	II	II	-	18195
12480	SAL_DA4561AA	II	II	-	18047
16204	SAL_DA4986AA	II	II	-	17717
16205	SAL_DA4987AA	II	II	-	17716
16507	SAL_DA4989AA	II	II	-	17714
16510	SAL_DA4992AA	II	II	-	17712
16509	SAL_DA4994AA	II	II	-	17710
13512	SAL_DA5253AA	II	II	-	17501
14584	SAL_DA5774AA	II	II	-	17051
16514	SAL_DA5934AA	II	II	-	16920
14006	SAL_DA6350AA	II	II	-	96399
14002	SAL_DA6664AA	II	II	-	16294
14004	SAL_DA6679AA	II	II	-	16279
13256	SAL_DA8095AA	II	II	-	15114
13249	SAL_DA8117AA	II	II	-	15092
14806	SAL_DA9095AA	II	II	-	14240
14103	SAL_DA9680AA	II	II	-	13713
14022	SAL_DA9788AA	II	II	-	13613
14291	SAL_EA0603AA	II	II	-	12905
16147	SAL_EA2466AA	II	II	-	11556
13519	SAL_EA2690AA	II	II	-	11378
16104	SAL_EA2817AA	II	II	-	11268
13335	SAL_EA2882AA	II	II	-	96321
16531	SAL_EA6970AA	II	II	-	7846
12270	SAL_EA7038AA	II	II	-	7782
11579	SAL_EA7838AA	II	II	-	7019
16542	SAL_EA7862AA	II	II	-	6996
16545	SAL_EA8014AA	II	II	-	6852
11801	SAL_EA8042AA	II	II	-	6824
14542	SAL_EA9347AA	II	II	-	5780
13800	SAL_FA0450AA	II	II	-	4912
16553	SAL_FA1233AA	II	II	-	4229
15340	SAL_FA1282AA	II	II	-	4183
13801	SAL_FA1334AA	II	II	-	4139
13672	SAL_FA6411AA	II	II	-	16294
13819	SAL_FA7289AA	II	II	-	40237
16564	SAL_FA8192AA	II	II	-	96476
16593	SAL_FA9273AA	II	II	-	46149
16219	SAL_FA9729AA	II	II	-	45360
12492	SAL_GA1788AA	II	II	-	43908
12497	SAL_GA1790AA	II	II	-	43906
12834	SAL_GA1815AA	II	II	-	43882
16581	SAL_GA2441AA	II	II	-	42340

12622	SAL_GA3282AA	II	II	-	42735
12632	SAL_GA3283AA	II	II	-	42734
12643	SAL_GA3284AA	II	II	-	42733
12631	SAL_GA3286AA	II	II	-	42731
12698	SAL_GA3287AA	II	II	-	42730
12704	SAL_GA3288AA	II	II	-	42729
12627	SAL_GA3289AA	II	II	-	42728
12657	SAL_GA3748AA	II	II	-	42354
16228	SAL_GA3749AA	II	II	-	42353
12640	SAL_GA3750AA	II	II	-	42352
16229	SAL_GA3751AA	II	II	-	44246
16230	SAL_GA3752AA	II	II	-	44245
12649	SAL_GA3755AA	II	II	-	42349
12481	SAL_GA3756AA	II	II	-	42348
12836	SAL_GA3874AA	II	II	-	42259
12835	SAL_GA3875AA	II	II	-	42258
12833	SAL_GA3876AA	II	II	-	42257
16568	SAL_GA3877AA	II	II	-	44231
16567	SAL_GA3878AA	II	II	-	44230
13318	SAL_GA4505AA	II	II	-	43727
16590	SAL_GA4919AA	II	II	-	45609
16599	SAL_GA6025AA	II	II	-	47533
16612	SAL_GA9071AA	II	II	-	49915
16611	SAL_GA9101AA	II	II	-	49933
16238	SAL_GA9630AA	II	II	-	50430
16239	SAL_GA9631AA	II	II	-	50436
16240	SAL_GA9632AA	II	II	-	50439
16241	SAL_GA9634AA	II	II	-	42258
16242	SAL_GA9635AA	II	II	-	43882
16618	SAL_GA9636AA	II	II	-	50440
16243	SAL_GA9637AA	II	II	-	51843
16651	SAL_HA2673AA	II	II	-	55150
16251	SAL_HA5523AA	II	II	-	54577
16640	SAL_HA5527AA	II	II	-	54458
16636	SAL_HA5528AA	II	II	-	54441
16133	SAL_HA5529AA	II	II	-	54403
16634	SAL_HA5532AA	II	II	-	54420
16252	SAL_HA5533AA	II	II	-	54422
16647	SAL_HA5563AA	II	II	-	96442
16654	SAL_HA6076AA	II	II	-	55849
16394	SAL_HA8861AA	II	II	-	58067
16405	SAL_HA8877AA	II	II	-	58051
16408	SAL_HA8881AA	II	II	-	58047
16407	SAL_HA8887AA	II	II	-	58041
16678	SAL_IA0403AA	II	II	-	59663
16683	SAL_IA4644AA	II	II	-	62618
16684	SAL_IA5157AA	II	II	-	62997
16698	SAL_IA7796AA	II	II	-	65126
16264	SAL_IA8901AA	II	II	-	65695
16096	SAL_JA4399AA	II	II	-	70494
16093	SAL_JA4402AA	II	II	-	70516
16091	SAL_JA4411AA	II	II	-	70539
16084	SAL_JA4415AA	II	II	-	70554
16087	SAL_JA4417AA	II	II	-	70547
16742	SAL_JA4488AA	II	II	-	70586
16743	SAL_JA4490AA	II	II	-	70590
16746	SAL_JA4493AA	II	II	-	70585
16770	SAL_JA5189AA	II	II	-	71268
16769	SAL_JA5190AA	II	II	-	71276
16764	SAL_JA5198AA	II	II	-	71308
16757	SAL_JA5212AA	II	II	-	71081
16787	SAL_JA6539AA	II	II	-	96669
16774	SAL_JA6580AA	II	II	-	71215
16810	SAL_JA6613AA	II	II	-	72576
16813	SAL_JA6977AA	II	II	-	72584
16824	SAL_JA8498AA	II	II	-	74098

16843	SAL_KA3175AA	II	II	-	78223
16846	SAL_KA5375AA	II	II	-	79717
16857	SAL_KA8174AA	II	II	-	81576
16870	SAL_LA2345AA	II	II	-	85581
16876	SAL_LA4893AA	II	II	-	87158
16886	SAL_LA7052AA	II	II	-	97877
17022	SAL_MA3023AA	II	II	-	94297
17040	SAL_MA3846AA	II	II	-	95179
16028	SAL_MA5798AA	II	II	-	96846
17053	SAL_MA5806AA	II	II	-	96846
16330	SAL_MA5807AA	II	II	-	96859
16027	SAL_MA5808AA	II	II	-	96856
16026	SAL_MA5810AA	II	II	-	96857
16331	SAL_MA5813AA	II	II	-	96874
17057	SAL_MA5816AA	II	II	-	96876
16023	SAL_MA5823AA	II	II	-	96882
16334	SAL_MA5824AA	II	II	-	96883
16335	SAL_MA5825AA	II	II	-	96884
16336	SAL_MA5832AA	II	II	-	96890
16020	SAL_MA5834AA	II	II	-	96893
16019	SAL_MA5837AA	II	II	-	96894
16017	SAL_MA5838AA	II	II	-	96896
17064	SAL_MA5840AA	II	II	-	96897
17172	SAL_MA5841AA	II	II	-	90089
17065	SAL_MA5842AA	II	II	-	96899
16337	SAL_MA5844AA	II	II	-	96900
17067	SAL_MA5846AA	II	II	-	96903
16015	SAL_MA5847AA	II	II	-	96902
16338	SAL_MA5849AA	II	II	-	96905
16013	SAL_MA5850AA	II	II	-	96907
17069	SAL_MA5852AA	II	II	-	96908
16012	SAL_MA5853AA	II	II	-	96908
16072	SAL_MA5857AA	II	II	-	96912
17071	SAL_MA5858AA	II	II	-	96911
16011	SAL_MA5860AA	II	II	-	96913
17073	SAL_MA5861AA	II	II	-	96916
16009	SAL_MA5870AA	II	II	-	97085
16006	SAL_MA5873AA	II	II	-	97078
16007	SAL_MA5876AA	II	II	-	97084
17079	SAL_MA5881AA	II	II	-	97079
16339	SAL_MA5882AA	II	II	-	97080
17080	SAL_MA5886AA	II	II	-	97082
16004	SAL_MA5887AA	II	II	-	97082
16340	SAL_MA5889AA	II	II	-	97083
17083	SAL_MA5893AA	II	II	-	97088
16005	SAL_MA5901AA	II	II	-	97091
17084	SAL_MA5902AA	II	II	-	97092
16001	SAL_MA5905AA	II	II	-	97097
17085	SAL_MA5906AA	II	II	-	97095
16148	SAL_MA5907AA	II	II	-	97098
17087	SAL_MA5909AA	II	II	-	97099
16003	SAL_MA5910AA	II	II	-	97096
16343	SAL_MA5916AA	II	II	-	97105
17088	SAL_MA5917AA	II	II	-	97102
16344	SAL_MA5918AA	II	II	-	97103
17089	SAL_MA5919AA	II	II	-	97104
17090	SAL_MA5920AA	II	II	-	97106
17091	SAL_MA5921AA	II	II	-	97107
15997	SAL_MA5923AA	II	II	-	97109
17092	SAL_MA5924AA	II	II	-	97110
17093	SAL_MA5925AA	II	II	-	97111
15995	SAL_MA5930AA	II	II	-	97116
17096	SAL_MA5934AA	II	II	-	97125
17097	SAL_MA5936AA	II	II	-	97130
17098	SAL_MA5937AA	II	II	-	97124
17099	SAL_MA5938AA	II	II	-	97136

15994	SAL_MA5939AA	II	II	-	97131	
17100	SAL_MA5941AA	II	II	-	97126	
15993	SAL_MA5942AA	II	II	-	97174	
15992	SAL_MA5943AA	II	II	-	97159	
17102	SAL_MA5945AA	II	II	-	97156	
16345	SAL_MA5946AA	II	II	-	97157	
17103	SAL_MA5949AA	II	II	-	97164	
15998	SAL_MA5950AA	II	II	-	97156	
15990	SAL_MA5952AA	II	II	-	97169	
17104	SAL_MA5953AA	II	II	-	97167	
15991	SAL_MA5954AA	II	II	-	97168	
17105	SAL_MA5956AA	II	II	-	97171	
17106	SAL_MA5957AA	II	II	-	97172	
16346	SAL_MA5958AA	II	II	-	97170	
16347	SAL_MA5967AA	II	II	-	97178	
16348	SAL_MA5968AA	II	II	-	97179	
17109	SAL_MA5969AA	II	II	-	97180	
15988	SAL_MA5970AA	II	II	-	97177	
16349	SAL_MA5971AA	II	II	-	97181	
17110	SAL_MA5973AA	II	II	-	97182	
15987	SAL_MA5974AA	II	II	-	97183	
15986	SAL_MA5978AA	II	II	-	97184	
15985	SAL_MA5979AA	II	II	-	97185	
15984	SAL_MA5983AA	II	II	-	97188	
16351	SAL_MA5984AA	II	II	-	97187	
15983	SAL_MA5986AA	II	II	-	97189	
15982	SAL_MA5988AA	II	II	-	97191	
16352	SAL_MA5989AA	II	II	-	97190	
15981	SAL_MA5992AA	II	II	-	97192	
15980	SAL_MA5994AA	II	II	-	97194	
17143	SAL_MA5995AA	II	II	-	97193	
17123	SAL_NA5011AA	II	II	-	104996	
17124	SAL_NA5012AA	II	II	-	104996	
17125	SAL_NA5013AA	II	II	-	105001	
17131	SAL_NA5024AA	II	II	-	105011	
15969	SAL_NA5032AA	II	II	-	105017	
16359	SAL_NA5035AA	II	II	-	105023	
16134	SAL_NA5036AA	II	II	-	105021	
17141	SAL_NA5046AA	II	II	-	105032	
17147	SAL_NA5065AA	II	II	-	105047	
15963	SAL_NA5078AA	II	II	-	105059	
16364	SAL_NA5081AA	II	II	-	105067	
17174	SAL_OA1859AA	II	II	-	110613	
17176	SAL_OA1861AA	II	II	-	110614	
16455	SAL_CA2337AA	IIIa	IIIa	-	28362	Yes
16136	SAL_CA2712AA	IIIa	IIIa	-	28035	Yes
13863	SAL_EA0784AA	IIIa	IIIa	-	96323	Yes
16768	SAL_JA5193AA	IIIa	IIIa	-	96673	Yes
16977	SAL_MA1774AA	IIIa	IIIa	-	93628	Yes
17113	SAL_MA6840AA	IIIa	IIIa	-	98554	Yes
17159	SAL_NA5086AA	IIIa	IIIa	-	105063	Yes
13162	SAL_BA1283AA	IIIa	IIIa	-	36537	
16165	SAL_BA1520AA	IIIa	IIIa	-	96328	
16166	SAL_BA1521AA	IIIa	IIIa	-	36353	
16525	SAL_BA7532AA	IIIa	IIIa	-	31782	
16435	SAL_BA7995AA	IIIa	IIIa	-	31523	
16422	SAL_BA8319AA	IIIa	IIIa	-	31325	
16519	SAL_BA8590AA	IIIa	IIIa	-	31128	
14920	SAL_BA8919AA	IIIa	IIIa	-	30854	
15347	SAL_BA9255AA	IIIa	IIIa	-	30590	
16446	SAL_CA0053AA	IIIa	IIIa	-	29950	
16451	SAL_CA1629AA	IIIa	IIIa	-	28840	
16410	SAL_CA1684AA	IIIa	IIIa	-	28787	
16452	SAL_CA2057AA	IIIa	IIIa	-	28603	
16454	SAL_CA2235AA	IIIa	IIIa	-	28452	
11635	SAL_CA2453AA	IIIa	IIIa	-	28262	

16458	SAL_CA2502AA	IIIa	IIIa	-	28221
11601	SAL_CA2540AA	IIIa	IIIa	-	28189
16460	SAL_CA2768AA	IIIa	IIIa	-	27989
16463	SAL_CA3204AA	IIIa	IIIa	-	27618
16467	SAL_CA3429AA	IIIa	IIIa	-	27420
16470	SAL_CA3889AA	IIIa	IIIa	-	27009
16478	SAL_CA4649AA	IIIa	IIIa	-	26349
16418	SAL_CA4700AA	IIIa	IIIa	-	26306
16480	SAL_CA5080AA	IIIa	IIIa	-	25984
16481	SAL_CA5081AA	IIIa	IIIa	-	25983
12084	SAL_CA5190AA	IIIa	IIIa	-	96381
16488	SAL_CA5904AA	IIIa	IIIa	-	25301
16492	SAL_CA6159AA	IIIa	IIIa	-	25075
11853	SAL_CA6656AA	IIIa	IIIa	-	96383
13607	SAL_CA7849AA	IIIa	IIIa	-	96348
15367	SAL_CA7988AA	IIIa	IIIa	-	23445
15376	SAL_CA8005AA	IIIa	IIIa	-	23428
15377	SAL_CA8023AA	IIIa	IIIa	-	23410
13460	SAL_CA8066AA	IIIa	IIIa	-	96385
16411	SAL_CA8208AA	IIIa	IIIa	-	96326
13919	SAL_CA8229AA	IIIa	IIIa	-	23227
13665	SAL_CA8254AA	IIIa	IIIa	-	96350
13918	SAL_CA8286AA	IIIa	IIIa	-	23174
13915	SAL_CA8685AA	IIIa	IIIa	-	22840
13913	SAL_CA8694AA	IIIa	IIIa	-	22831
13916	SAL_CA8707AA	IIIa	IIIa	-	22819
13914	SAL_CA8727AA	IIIa	IIIa	-	22801
13911	SAL_CA8767AA	IIIa	IIIa	-	22766
15374	SAL_CA8795AA	IIIa	IIIa	-	22740
13907	SAL_CA8806AA	IIIa	IIIa	-	22729
13908	SAL_CA8826AA	IIIa	IIIa	-	11403
13909	SAL_CA8834AA	IIIa	IIIa	-	22704
13912	SAL_CA8843AA	IIIa	IIIa	-	22697
13630	SAL_CA8919AA	IIIa	IIIa	-	22633
14448	SAL_DA1033AA	IIIa	IIIa	-	20868
14380	SAL_DA1238AA	IIIa	IIIa	-	20672
14369	SAL_DA1412AA	IIIa	IIIa	-	20511
14474	SAL_DA1531AA	IIIa	IIIa	-	20404
15118	SAL_DA1745AA	IIIa	IIIa	-	96356
15368	SAL_DA2205AA	IIIa	IIIa	-	11403
12535	SAL_DA2250AA	IIIa	IIIa	-	19818
15366	SAL_DA2461AA	IIIa	IIIa	-	19641
14614	SAL_DA3038AA	IIIa	IIIa	-	96386
12828	SAL_DA3229AA	IIIa	IIIa	-	11403
15362	SAL_DA4055AA	IIIa	IIIa	-	96388
15353	SAL_DA4362AA	IIIa	IIIa	-	15958
13229	SAL_DA4978AA	IIIa	IIIa	-	96394
12886	SAL_DA4979AA	IIIa	IIIa	-	96393
16506	SAL_DA4985AA	IIIa	IIIa	-	96395
16409	SAL_DA4995AA	IIIa	IIIa	-	96322
16512	SAL_DA5007AA	IIIa	IIIa	-	96316
12623	SAL_DA5010AA	IIIa	IIIa	-	96327
16513	SAL_DA5015AA	IIIa	IIIa	-	96397
15360	SAL_DA5370AA	IIIa	IIIa	-	17388
12817	SAL_DA5380AA	IIIa	IIIa	-	17378
15359	SAL_DA5433AA	IIIa	IIIa	-	17331
13961	SAL_DA5543AA	IIIa	IIIa	-	96398
15396	SAL_DA6883AA	IIIa	IIIa	-	16114
13268	SAL_DA6945AA	IIIa	IIIa	-	96365
13904	SAL_DA7060AA	IIIa	IIIa	-	15958
13903	SAL_DA7125AA	IIIa	IIIa	-	15906
15395	SAL_DA8069AA	IIIa	IIIa	-	15138
14122	SAL_DA8445AA	IIIa	IIIa	-	96358
13972	SAL_DA8812AA	IIIa	IIIa	-	14483
15392	SAL_DA9359AA	IIIa	IIIa	-	13994
16106	SAL_DA9590AA	IIIa	IIIa	-	96324

13246	SAL_DA9619AA	IIIa	IIIa	-	13769
13965	SAL_DA9990AA	IIIa	IIIa	-	96403
14926	SAL_EA0155AA	IIIa	IIIa	-	96337
13232	SAL_EA0728AA	IIIa	IIIa	-	96405
13136	SAL_EA2100AA	IIIa	IIIa	-	96410
15384	SAL_EA2654AA	IIIa	IIIa	-	11403
15386	SAL_EA2656AA	IIIa	IIIa	-	11403
15385	SAL_EA2658AA	IIIa	IIIa	-	11402
15387	SAL_EA2659AA	IIIa	IIIa	-	11401
15388	SAL_EA2663AA	IIIa	IIIa	-	11398
13171	SAL_EA2962AA	IIIa	IIIa	-	11152
16517	SAL_EA4647AA	IIIa	IIIa	-	96413
16518	SAL_EA4659AA	IIIa	IIIa	-	9682
15018	SAL_EA5304AA	IIIa	IIIa	-	96507
16529	SAL_EA5966AA	IIIa	IIIa	-	8639
15334	SAL_EA6754AA	IIIa	IIIa	-	8029
16530	SAL_EA6921AA	IIIa	IIIa	-	7891
16533	SAL_EA7037AA	IIIa	IIIa	-	7783
16535	SAL_EA7132AA	IIIa	IIIa	-	7693
16536	SAL_EA7137AA	IIIa	IIIa	-	7690
16540	SAL_EA7603AA	IIIa	IIIa	-	7246
16547	SAL_EA9016AA	IIIa	IIIa	-	6038
15146	SAL_EA9749AA	IIIa	IIIa	-	96429
15151	SAL_EA9778AA	IIIa	IIIa	-	96406
15039	SAL_FA1101AA	IIIa	IIIa	-	96433
15051	SAL_FA1474AA	IIIa	IIIa	-	96435
16560	SAL_FA4129AA	IIIa	IIIa	-	1830
12325	SAL_FA4533AA	IIIa	IIIa	-	96499
16557	SAL_FA4616AA	IIIa	IIIa	-	1379
13446	SAL_FA7831AA	IIIa	IIIa	-	96497
13595	SAL_FA7833AA	IIIa	IIIa	-	96449
16217	SAL_FA8656AA	IIIa	IIIa	-	47226
16598	SAL_FA8797AA	IIIa	IIIa	-	47108
16594	SAL_FA9160AA	IIIa	IIIa	-	46319
16588	SAL_FA9600AA	IIIa	IIIa	-	45471
12855	SAL_GA1699AA	IIIa	IIIa	-	96471
14906	SAL_GA1756AA	IIIa	IIIa	-	43935
14905	SAL_GA1771AA	IIIa	IIIa	-	43922
12852	SAL_GA1777AA	IIIa	IIIa	-	43917
12850	SAL_GA1778AA	IIIa	IIIa	-	43923
12838	SAL_GA1814AA	IIIa	IIIa	-	43885
16582	SAL_GA2186AA	IIIa	IIIa	-	43536
16580	SAL_GA2456AA	IIIa	IIIa	-	96475
16574	SAL_GA2479AA	IIIa	IIIa	-	96473
12853	SAL_GA3192AA	IIIa	IIIa	-	42819
14903	SAL_GA3241AA	IIIa	IIIa	-	42776
14902	SAL_GA3256AA	IIIa	IIIa	-	42760
16573	SAL_GA3366AA	IIIa	IIIa	-	96480
12851	SAL_GA3710AA	IIIa	IIIa	-	42385
12755	SAL_GA3757AA	IIIa	IIIa	-	42347
12648	SAL_GA3758AA	IIIa	IIIa	-	42346
12630	SAL_GA3759AA	IIIa	IIIa	-	42345
12839	SAL_GA3872AA	IIIa	IIIa	-	42261
12837	SAL_GA3873AA	IIIa	IIIa	-	42260
16592	SAL_GA5146AA	IIIa	IIIa	-	96496
16595	SAL_GA5718AA	IIIa	IIIa	-	46505
16237	SAL_GA7376AA	IIIa	IIIa	-	96494
16609	SAL_GA8878AA	IIIa	IIIa	-	96465
16610	SAL_GA9051AA	IIIa	IIIa	-	49838
16616	SAL_GA9565AA	IIIa	IIIa	-	96418
16615	SAL_GA9569AA	IIIa	IIIa	-	96419
16101	SAL_GA9629AA	IIIa	IIIa	-	96420
16619	SAL_HA0057AA	IIIa	IIIa	-	96423
16621	SAL_HA0252AA	IIIa	IIIa	-	96425
16622	SAL_HA0847AA	IIIa	IIIa	-	96439
16247	SAL_HA4663AA	IIIa	IIIa	-	53618

16249	SAL_HA4665AA	IIIa	IIIa	-	53619
16098	SAL_HA4666AA	IIIa	IIIa	-	53721
16632	SAL_HA4667AA	IIIa	IIIa	-	53723
16630	SAL_HA4668AA	IIIa	IIIa	-	53584
16629	SAL_HA4670AA	IIIa	IIIa	-	54586
16644	SAL_HA5570AA	IIIa	IIIa	-	96441
16655	SAL_HA6077AA	IIIa	IIIa	-	96457
16657	SAL_HA6146AA	IIIa	IIIa	-	56787
16395	SAL_HA8845AA	IIIa	IIIa	-	58083
16396	SAL_HA8848AA	IIIa	IIIa	-	96461
16397	SAL_HA8849AA	IIIa	IIIa	-	96460
16398	SAL_HA8851AA	IIIa	IIIa	-	96462
16399	SAL_HA8852AA	IIIa	IIIa	-	96463
16400	SAL_HA8853AA	IIIa	IIIa	-	58075
16401	SAL_HA8854AA	IIIa	IIIa	-	96464
16403	SAL_HA8857AA	IIIa	IIIa	-	96466
16406	SAL_HA8865AA	IIIa	IIIa	-	58063
16672	SAL_HA9527AA	IIIa	IIIa	-	96451
16676	SAL_IA0417AA	IIIa	IIIa	-	96509
16675	SAL_IA0434AA	IIIa	IIIa	-	96510
16681	SAL_IA3204AA	IIIa	IIIa	-	61440
16690	SAL_IA6418AA	IIIa	IIIa	-	96520
16691	SAL_IA6495AA	IIIa	IIIa	-	96521
16693	SAL_IA6995AA	IIIa	IIIa	-	64248
16695	SAL_IA7497AA	IIIa	IIIa	-	64863
16700	SAL_IA8150AA	IIIa	IIIa	-	65329
16702	SAL_IA8854AA	IIIa	IIIa	-	96536
16708	SAL_IA9199AA	IIIa	IIIa	-	66410
16711	SAL_IA9799AA	IIIa	IIIa	-	66649
16714	SAL_JA0186AA	IIIa	IIIa	-	96562
16733	SAL_JA2682AA	IIIa	IIIa	-	68920
16097	SAL_JA4396AA	IIIa	IIIa	-	70493
16088	SAL_JA4420AA	IIIa	IIIa	-	70546
16740	SAL_JA4481AA	IIIa	IIIa	-	74147
16744	SAL_JA4491AA	IIIa	IIIa	-	70595
16750	SAL_JA5069AA	IIIa	IIIa	-	71014
16763	SAL_JA5199AA	IIIa	IIIa	-	96671
16760	SAL_JA5207AA	IIIa	IIIa	-	71086
16752	SAL_JA5225AA	IIIa	IIIa	-	71054
16751	SAL_JA5229AA	IIIa	IIIa	-	71062
16807	SAL_JA5599AA	IIIa	IIIa	-	71886
16806	SAL_JA5654AA	IIIa	IIIa	-	71945
16803	SAL_JA5831AA	IIIa	IIIa	-	71797
16798	SAL_JA6342AA	IIIa	IIIa	-	71351
16079	SAL_JA6424AA	IIIa	IIIa	-	71267
16078	SAL_JA6426AA	IIIa	IIIa	-	96645
16273	SAL_JA6534AA	IIIa	IIIa	-	71164
16780	SAL_JA6565AA	IIIa	IIIa	-	71169
16779	SAL_JA6566AA	IIIa	IIIa	-	71157
16777	SAL_JA6570AA	IIIa	IIIa	-	71146
16776	SAL_JA6571AA	IIIa	IIIa	-	71147
16773	SAL_JA6581AA	IIIa	IIIa	-	71128
16809	SAL_JA6848AA	IIIa	IIIa	-	72347
16815	SAL_JA7066AA	IIIa	IIIa	-	72780
16821	SAL_JA7885AA	IIIa	IIIa	-	73491
16820	SAL_JA7930AA	IIIa	IIIa	-	73470
16817	SAL_JA8166AA	IIIa	IIIa	-	73301
16825	SAL_JA9607AA	IIIa	IIIa	-	74219
16281	SAL_JA9655AA	IIIa	IIIa	-	74232
16830	SAL_KA0412AA	IIIa	IIIa	-	96799
16841	SAL_KA2567AA	IIIa	IIIa	-	97711
16840	SAL_KA2573AA	IIIa	IIIa	-	77542
16839	SAL_KA2904AA	IIIa	IIIa	-	96850
16847	SAL_KA5647AA	IIIa	IIIa	-	97753
16850	SAL_KA6337AA	IIIa	IIIa	-	80629
16856	SAL_KA7829AA	IIIa	IIIa	-	82100

16869	SAL_LA0557AA	IIIa	IIIa	-	83810
16871	SAL_LA3930AA	IIIa	IIIa	-	86484
16880	SAL_LA6318AA	IIIa	IIIa	-	97875
16878	SAL_LA6392AA	IIIa	IIIa	-	88761
16887	SAL_LA9712AA	IIIa	IIIa	-	97884
16889	SAL_MA1470AA	IIIa	IIIa	-	92962
16987	SAL_MA1727AA	IIIa	IIIa	-	93414
16988	SAL_MA1728AA	IIIa	IIIa	-	93450
16985	SAL_MA1739AA	IIIa	IIIa	-	93417
16984	SAL_MA1743AA	IIIa	IIIa	-	93291
16979	SAL_MA1772AA	IIIa	IIIa	-	93490
16978	SAL_MA1773AA	IIIa	IIIa	-	93454
16135	SAL_MA1784AA	IIIa	IIIa	-	93301
16975	SAL_MA1789AA	IIIa	IIIa	-	93302
16974	SAL_MA1791AA	IIIa	IIIa	-	93303
16291	SAL_MA1792AA	IIIa	IIIa	-	96910
16972	SAL_MA1799AA	IIIa	IIIa	-	93312
16971	SAL_MA1805AA	IIIa	IIIa	-	93491
16966	SAL_MA1823AA	IIIa	IIIa	-	93373
16965	SAL_MA1826AA	IIIa	IIIa	-	93376
16963	SAL_MA1833AA	IIIa	IIIa	-	93380
16960	SAL_MA1846AA	IIIa	IIIa	-	93228
16959	SAL_MA1852AA	IIIa	IIIa	-	93402
16958	SAL_MA1853AA	IIIa	IIIa	-	93232
16957	SAL_MA1861AA	IIIa	IIIa	-	93632
16956	SAL_MA1862AA	IIIa	IIIa	-	93633
16955	SAL_MA1863AA	IIIa	IIIa	-	93634
16954	SAL_MA1870AA	IIIa	IIIa	-	93636
16953	SAL_MA1878AA	IIIa	IIIa	-	93246
16945	SAL_MA1923AA	IIIa	IIIa	-	93660
16942	SAL_MA1939AA	IIIa	IIIa	-	93669
16941	SAL_MA1940AA	IIIa	IIIa	-	93670
16940	SAL_MA1945AA	IIIa	IIIa	-	93678
16299	SAL_MA1949AA	IIIa	IIIa	-	93675
16936	SAL_MA1974AA	IIIa	IIIa	-	93669
16935	SAL_MA1976AA	IIIa	IIIa	-	93595
16929	SAL_MA2027AA	IIIa	IIIa	-	93273
16926	SAL_MA2037AA	IIIa	IIIa	-	93317
16924	SAL_MA2051AA	IIIa	IIIa	-	93730
16922	SAL_MA2053AA	IIIa	IIIa	-	93330
16921	SAL_MA2054AA	IIIa	IIIa	-	93731
16917	SAL_MA2076AA	IIIa	IIIa	-	93356
16915	SAL_MA2082AA	IIIa	IIIa	-	93367
16913	SAL_MA2103AA	IIIa	IIIa	-	93181
16907	SAL_MA2131AA	IIIa	IIIa	-	93192
16905	SAL_MA2138AA	IIIa	IIIa	-	93162
16904	SAL_MA2144AA	IIIa	IIIa	-	93199
16898	SAL_MA2195AA	IIIa	IIIa	-	93214
16894	SAL_MA2207AA	IIIa	IIIa	-	93218
16316	SAL_MA2219AA	IIIa	IIIa	-	93220
17016	SAL_MA2354AA	IIIa	IIIa	-	93301
17015	SAL_MA2355AA	IIIa	IIIa	-	93876
16039	SAL_MA2363AA	IIIa	IIIa	-	93859
17013	SAL_MA2368AA	IIIa	IIIa	-	93878
17010	SAL_MA2374AA	IIIa	IIIa	-	93868
17006	SAL_MA2382AA	IIIa	IIIa	-	93866
17004	SAL_MA2392AA	IIIa	IIIa	-	93883
17000	SAL_MA2404AA	IIIa	IIIa	-	93767
16999	SAL_MA2407AA	IIIa	IIIa	-	93777
16995	SAL_MA2425AA	IIIa	IIIa	-	93871
16994	SAL_MA2436AA	IIIa	IIIa	-	93785
16990	SAL_MA2456AA	IIIa	IIIa	-	93815
17025	SAL_MA3252AA	IIIa	IIIa	-	94563
17038	SAL_MA3957AA	IIIa	IIIa	-	93312
17036	SAL_MA3964AA	IIIa	IIIa	-	95037
17035	SAL_MA3969AA	IIIa	IIIa	-	95053

16326	SAL_MA3997AA	IIIa	IIIa	-	93301	
17029	SAL_MA4009AA	IIIa	IIIa	-	95081	
17026	SAL_MA4022AA	IIIa	IIIa	-	95026	
16031	SAL_MA4425AA	IIIa	IIIa	-	95199	
17046	SAL_MA4434AA	IIIa	IIIa	-	95227	
17044	SAL_MA4439AA	IIIa	IIIa	-	95232	
17042	SAL_MA4445AA	IIIa	IIIa	-	95202	
17049	SAL_MA5797AA	IIIa	IIIa	-	96836	
17063	SAL_MA5836AA	IIIa	IIIa	-	96892	
15999	SAL_MA5911AA	IIIa	IIIa	-	97101	
17094	SAL_MA5928AA	IIIa	IIIa	-	97112	
17122	SAL_NA5008AA	IIIa	IIIa	-	104993	
15975	SAL_NA5009AA	IIIa	IIIa	-	104998	
17130	SAL_NA5023AA	IIIa	IIIa	-	105009	
17139	SAL_NA5040AA	IIIa	IIIa	-	105024	
16361	SAL_NA5043AA	IIIa	IIIa	-	105027	
17148	SAL_NA5066AA	IIIa	IIIa	-	105045	
17167	SAL_OA0441AA	IIIa	IIIa	-	109501	
17169	SAL_OA0445AA	IIIa	IIIa	-	109503	
16368	SAL_OA0446AA	IIIa	IIIa	-	109505	
17171	SAL_OA0449AA	IIIa	IIIa	-	109520	
17177	SAL_OA1862AA	IIIa	IIIa	-	Nan	
11643	SAL_CA1324AA	IIIb	IIIb	-	29047	Yes
12465	SAL_GA3763AA	IIIb	IIIb	-	42341	Yes
16789	SAL_JA6522AA	IIIb	IIIb	-	71179	Yes
16986	SAL_MA1737AA	IIIb	IIIb	-	93626	Yes
16024	SAL_MA5817AA	IIIb	IIIb	-	96877	Yes
17156	SAL_NA5075AA	IIIb	IIIb	-	105057	Yes
17160	SAL_NA7238AA	IIIb	IIIb	-	106868	Yes
13476	SAL_BA1624AA	IIIb	IIIb	-	96335	
13065	SAL_BA1671AA	IIIb	IIIb	-	36226	
13482	SAL_BA1713AA	IIIb	IIIb	-	36187	
13401	SAL_BA1942AA	IIIb	IIIb	-	36004	
13545	SAL_BA2227AA	IIIb	IIIb	-	35764	
13544	SAL_BA2230AA	IIIb	IIIb	-	35761	
13543	SAL_BA2248AA	IIIb	IIIb	-	35746	
14921	SAL_BA3015AA	IIIb	IIIb	-	35139	
14919	SAL_BA3045AA	IIIb	IIIb	-	35113	
12963	SAL_BA3532AA	IIIb	IIIb	-	34733	
13585	SAL_BA7272AA	IIIb	IIIb	-	31965	
12250	SAL_BA7455AA	IIIb	IIIb	-	31828	
16420	SAL_BA7500AA	IIIb	IIIb	-	31800	
12207	SAL_BA7533AA	IIIb	IIIb	-	96334	
16428	SAL_BA7586AA	IIIb	IIIb	-	31760	
16429	SAL_BA7596AA	IIIb	IIIb	-	31757	
11933	SAL_BA7628AA	IIIb	IIIb	-	31740	
16431	SAL_BA7841AA	IIIb	IIIb	-	31613	
16432	SAL_BA7856AA	IIIb	IIIb	-	31599	
16433	SAL_BA7943AA	IIIb	IIIb	-	31552	
12028	SAL_BA8062AA	IIIb	IIIb	-	31491	
11733	SAL_BA8426AA	IIIb	IIIb	-	31260	
15348	SAL_BA9245AA	IIIb	IIIb	-	30596	
16440	SAL_BA9267AA	IIIb	IIIb	-	30579	
16441	SAL_BA9278AA	IIIb	IIIb	-	30568	
16442	SAL_BA9312AA	IIIb	IIIb	-	28123	
11973	SAL_BA9335AA	IIIb	IIIb	-	30521	
16443	SAL_BA9402AA	IIIb	IIIb	-	26404	
16445	SAL_BA9696AA	IIIb	IIIb	-	30225	
11704	SAL_BA9947AA	IIIb	IIIb	-	30036	
16447	SAL_CA0036AA	IIIb	IIIb	-	29965	
11517	SAL_CA0789AA	IIIb	IIIb	-	29345	
16450	SAL_CA0904AA	IIIb	IIIb	-	29254	
12006	SAL_CA1203AA	IIIb	IIIb	-	96375	
11783	SAL_CA1249AA	IIIb	IIIb	-	29086	
11873	SAL_CA1368AA	IIIb	IIIb	-	29034	
11631	SAL_CA1476AA	IIIb	IIIb	-	28979	

16417	SAL_CA1507AA	IIIb	IIIb	-	28952
12397	SAL_CA1587AA	IIIb	IIIb	-	28876
12222	SAL_CA2401AA	IIIb	IIIb	-	28310
11745	SAL_CA2452AA	IIIb	IIIb	-	28263
16456	SAL_CA2481AA	IIIb	IIIb	-	28238
16416	SAL_CA2614AA	IIIb	IIIb	-	28123
16459	SAL_CA2746AA	IIIb	IIIb	-	28007
12027	SAL_CA2899AA	IIIb	IIIb	-	27879
16425	SAL_CA2984AA	IIIb	IIIb	-	27807
16464	SAL_CA3218AA	IIIb	IIIb	-	27606
16466	SAL_CA3389AA	IIIb	IIIb	-	27456
11912	SAL_CA4009AA	IIIb	IIIb	-	26900
11711	SAL_CA4041AA	IIIb	IIIb	-	26871
16473	SAL_CA4256AA	IIIb	IIIb	-	26687
16475	SAL_CA4490AA	IIIb	IIIb	-	26486
16477	SAL_CA4589AA	IIIb	IIIb	-	26404
16482	SAL_CA5092AA	IIIb	IIIb	-	25975
16526	SAL_CA5110AA	IIIb	IIIb	-	25961
16421	SAL_CA5242AA	IIIb	IIIb	-	25860
16483	SAL_CA5255AA	IIIb	IIIb	-	25847
11765	SAL_CA5288AA	IIIb	IIIb	-	25819
16484	SAL_CA5543AA	IIIb	IIIb	-	25608
12069	SAL_CA5673AA	IIIb	IIIb	-	25496
13422	SAL_CA7172AA	IIIb	IIIb	-	24159
15375	SAL_CA8383AA	IIIb	IIIb	-	23094
14522	SAL_DA1038AA	IIIb	IIIb	-	20863
14530	SAL_DA1304AA	IIIb	IIIb	-	20610
14483	SAL_DA1341AA	IIIb	IIIb	-	20579
14508	SAL_DA1347AA	IIIb	IIIb	-	20573
14529	SAL_DA1353AA	IIIb	IIIb	-	20568
14511	SAL_DA1359AA	IIIb	IIIb	-	20562
14521	SAL_DA1437AA	IIIb	IIIb	-	20486
14526	SAL_DA1440AA	IIIb	IIIb	-	20483
14520	SAL_DA1513AA	IIIb	IIIb	-	20422
14518	SAL_DA1517AA	IIIb	IIIb	-	20418
14524	SAL_DA1521AA	IIIb	IIIb	-	20414
14510	SAL_DA1528AA	IIIb	IIIb	-	20407
15117	SAL_DA1696AA	IIIb	IIIb	-	20272
14830	SAL_DA2665AA	IIIb	IIIb	-	19494
16494	SAL_DA2995AA	IIIb	IIIb	-	19278
16495	SAL_DA3050AA	IIIb	IIIb	-	19241
16496	SAL_DA3078AA	IIIb	IIIb	-	19226
14824	SAL_DA3432AA	IIIb	IIIb	-	18944
12935	SAL_DA3629AA	IIIb	IIIb	-	18768
12933	SAL_DA3630AA	IIIb	IIIb	-	18768
12934	SAL_DA3705AA	IIIb	IIIb	-	18768
12932	SAL_DA3721AA	IIIb	IIIb	-	18754
13925	SAL_DA4222AA	IIIb	IIIb	-	18315
16505	SAL_DA4980AA	IIIb	IIIb	-	17723
16508	SAL_DA4988AA	IIIb	IIIb	-	96364
16511	SAL_DA5006AA	IIIb	IIIb	-	17699
16516	SAL_DA5796AA	IIIb	IIIb	-	96320
14005	SAL_DA6338AA	IIIb	IIIb	-	16563
13272	SAL_DA6342AA	IIIb	IIIb	-	16559
13273	SAL_DA6346AA	IIIb	IIIb	-	96400
13995	SAL_DA6956AA	IIIb	IIIb	-	16049
13991	SAL_DA7233AA	IIIb	IIIb	-	15805
13260	SAL_DA7498AA	IIIb	IIIb	-	15599
13252	SAL_DA8098AA	IIIb	IIIb	-	15111
14123	SAL_DA8439AA	IIIb	IIIb	-	14806
14137	SAL_DA8576AA	IIIb	IIIb	-	14687
14121	SAL_DA8672AA	IIIb	IIIb	-	14598
15292	SAL_DA8766AA	IIIb	IIIb	-	14518
15288	SAL_DA9128AA	IIIb	IIIb	-	14213
14179	SAL_DA9655AA	IIIb	IIIb	-	13737
14180	SAL_DA9667AA	IIIb	IIIb	-	13726

14782	SAL_DA9786AA	IIIb	IIIb	-	13615
13236	SAL_DA9997AA	IIIb	IIIb	-	13432
14787	SAL_EA0246AA	IIIb	IIIb	-	13212
13286	SAL_EA0280AA	IIIb	IIIb	-	13187
16515	SAL_EA0362AA	IIIb	IIIb	-	13114
13235	SAL_EA0493AA	IIIb	IIIb	-	12997
13856	SAL_EA0576AA	IIIb	IIIb	-	12929
13521	SAL_EA2705AA	IIIb	IIIb	-	11366
14660	SAL_EA4686AA	IIIb	IIIb	-	9658
16528	SAL_EA5548AA	IIIb	IIIb	-	8992
16532	SAL_EA6977AA	IIIb	IIIb	-	7839
11766	SAL_EA6993AA	IIIb	IIIb	-	7823
16534	SAL_EA7069AA	IIIb	IIIb	-	7752
16537	SAL_EA7206AA	IIIb	IIIb	-	7621
11694	SAL_EA7714AA	IIIb	IIIb	-	7140
16541	SAL_EA7719AA	IIIb	IIIb	-	7135
16137	SAL_EA7795AA	IIIb	IIIb	-	7061
16107	SAL_EA7923AA	IIIb	IIIb	-	6940
16543	SAL_EA7928AA	IIIb	IIIb	-	6935
16544	SAL_EA7971AA	IIIb	IIIb	-	96424
12048	SAL_EA7994AA	IIIb	IIIb	-	6870
13443	SAL_EA8681AA	IIIb	IIIb	-	6300
13102	SAL_EA8955AA	IIIb	IIIb	-	1
16550	SAL_FA0588AA	IIIb	IIIb	-	4787
16551	SAL_FA0775AA	IIIb	IIIb	-	4640
15341	SAL_FA3297AA	IIIb	IIIb	-	96501
16555	SAL_FA3956AA	IIIb	IIIb	-	96500
16561	SAL_FA4093AA	IIIb	IIIb	-	1860
16559	SAL_FA4366AA	IIIb	IIIb	-	1614
16558	SAL_FA4589AA	IIIb	IIIb	-	1405
16556	SAL_FA4650AA	IIIb	IIIb	-	1346
16563	SAL_FA5521AA	IIIb	IIIb	-	610
16562	SAL_FA5555AA	IIIb	IIIb	-	582
15459	SAL_FA5568AA	IIIb	IIIb	-	96444
13427	SAL_FA5696AA	IIIb	IIIb	-	474
13593	SAL_FA5824AA	IIIb	IIIb	-	360
16214	SAL_FA7309AA	IIIb	IIIb	-	40245
12455	SAL_GA1618AA	IIIb	IIIb	-	58344
12454	SAL_GA1619AA	IIIb	IIIb	-	58344
15400	SAL_GA1620AA	IIIb	IIIb	-	58343
12842	SAL_GA1813AA	IIIb	IIIb	-	43886
16586	SAL_GA1985AA	IIIb	IIIb	-	40881
16584	SAL_GA2081AA	IIIb	IIIb	-	43623
16583	SAL_GA2085AA	IIIb	IIIb	-	43620
16566	SAL_GA2760AA	IIIb	IIIb	-	41934
16565	SAL_GA2804AA	IIIb	IIIb	-	41890
12885	SAL_GA3475AA	IIIb	IIIb	-	42570
12854	SAL_GA3555AA	IIIb	IIIb	-	42510
12715	SAL_GA3760AA	IIIb	IIIb	-	42344
12485	SAL_GA3761AA	IIIb	IIIb	-	42343
12486	SAL_GA3762AA	IIIb	IIIb	-	42342
16232	SAL_GA3868AA	IIIb	IIIb	-	44232
12843	SAL_GA3869AA	IIIb	IIIb	-	42264
12840	SAL_GA3871AA	IIIb	IIIb	-	42262
16589	SAL_GA4836AA	IIIb	IIIb	-	45570
16597	SAL_GA5427AA	IIIb	IIIb	-	46726
16596	SAL_GA5497AA	IIIb	IIIb	-	96495
16233	SAL_GA6035AA	IIIb	IIIb	-	47523
16600	SAL_GA6577AA	IIIb	IIIb	-	48052
16605	SAL_GA7006AA	IIIb	IIIb	-	48442
16234	SAL_GA7008AA	IIIb	IIIb	-	48440
16604	SAL_GA7014AA	IIIb	IIIb	-	48430
16603	SAL_GA7029AA	IIIb	IIIb	-	48423
16602	SAL_GA7070AA	IIIb	IIIb	-	48348
16601	SAL_GA7092AA	IIIb	IIIb	-	48384
16235	SAL_GA7120AA	IIIb	IIIb	-	48340

16236	SAL_GA7129AA	IIIb	IIIb	-	48354
16625	SAL_GA9692AA	IIIb	IIIb	-	51851
16617	SAL_GA9774AA	IIIb	IIIb	-	96447
16620	SAL_HA0334AA	IIIb	IIIb	-	50736
16244	SAL_HA3932AA	IIIb	IIIb	-	55218
16652	SAL_HA4100AA	IIIb	IIIb	-	55313
16633	SAL_HA4518AA	IIIb	IIIb	-	53729
16245	SAL_HA4609AA	IIIb	IIIb	-	53616
16631	SAL_HA4610AA	IIIb	IIIb	-	53720
16246	SAL_HA4616AA	IIIb	IIIb	-	53610
16248	SAL_HA4664AA	IIIb	IIIb	-	53695
16250	SAL_HA4669AA	IIIb	IIIb	-	53585
16628	SAL_HA4671AA	IIIb	IIIb	-	53586
16627	SAL_HA4672AA	IIIb	IIIb	-	53587
16650	SAL_HA5556AA	IIIb	IIIb	-	54816
16648	SAL_HA5560AA	IIIb	IIIb	-	54847
16643	SAL_HA5581AA	IIIb	IIIb	-	54835
16661	SAL_HA6267AA	IIIb	IIIb	-	57110
16253	SAL_HA6416AA	IIIb	IIIb	-	55074
16653	SAL_HA6709AA	IIIb	IIIb	-	55526
16665	SAL_HA8754AA	IIIb	IIIb	-	57980
16390	SAL_HA8835AA	IIIb	IIIb	-	58093
16373	SAL_HA8882AA	IIIb	IIIb	-	58046
16389	SAL_HA8897AA	IIIb	IIIb	-	58030
16404	SAL_HA8898AA	IIIb	IIIb	-	58031
16680	SAL_IA1830AA	IIIb	IIIb	-	59861
16694	SAL_IA7509AA	IIIb	IIIb	-	64874
16701	SAL_IA8335AA	IIIb	IIIb	-	65368
16705	SAL_IA8727AA	IIIb	IIIb	-	65811
16704	SAL_IA8779AA	IIIb	IIIb	-	65783
16712	SAL_IA9856AA	IIIb	IIIb	-	66702
16717	SAL_JA0117AA	IIIb	IIIb	-	67058
16729	SAL_JA0479AA	IIIb	IIIb	-	68054
16728	SAL_JA0480AA	IIIb	IIIb	-	68055
16727	SAL_JA0518AA	IIIb	IIIb	-	68181
16726	SAL_JA1106AA	IIIb	IIIb	-	67763
16725	SAL_JA1172AA	IIIb	IIIb	-	67714
16724	SAL_JA1431AA	IIIb	IIIb	-	67485
16723	SAL_JA1434AA	IIIb	IIIb	-	67484
16722	SAL_JA1436AA	IIIb	IIIb	-	67500
16719	SAL_JA1453AA	IIIb	IIIb	-	67384
16732	SAL_JA1777AA	IIIb	IIIb	-	96583
16731	SAL_JA1784AA	IIIb	IIIb	-	68290
16735	SAL_JA3626AA	IIIb	IIIb	-	42343
16082	SAL_JA4419AA	IIIb	IIIb	-	70555
16739	SAL_JA4422AA	IIIb	IIIb	-	70552
16086	SAL_JA4426AA	IIIb	IIIb	-	70550
16741	SAL_JA4486AA	IIIb	IIIb	-	70594
16745	SAL_JA4492AA	IIIb	IIIb	-	70589
16758	SAL_JA5210AA	IIIb	IIIb	-	71115
16755	SAL_JA5215AA	IIIb	IIIb	-	71075
16754	SAL_JA5216AA	IIIb	IIIb	-	96664
16753	SAL_JA5222AA	IIIb	IIIb	-	72068
16805	SAL_JA5766AA	IIIb	IIIb	-	71828
16804	SAL_JA5813AA	IIIb	IIIb	-	71806
16802	SAL_JA5967AA	IIIb	IIIb	-	71660
16801	SAL_JA5988AA	IIIb	IIIb	-	71651
16799	SAL_JA6336AA	IIIb	IIIb	-	71349
16797	SAL_JA6350AA	IIIb	IIIb	-	71321
16794	SAL_JA6450AA	IIIb	IIIb	-	71270
16793	SAL_JA6496AA	IIIb	IIIb	-	71210
16792	SAL_JA6514AA	IIIb	IIIb	-	71201
16790	SAL_JA6521AA	IIIb	IIIb	-	71193
16786	SAL_JA6548AA	IIIb	IIIb	-	71170
16784	SAL_JA6553AA	IIIb	IIIb	-	71188
16783	SAL_JA6555AA	IIIb	IIIb	-	71159

16782	SAL_JA6556AA	IIIb	IIIb	-	96667
16778	SAL_JA6569AA	IIIb	IIIb	-	71143
16811	SAL_JA6985AA	IIIb	IIIb	-	72589
16823	SAL_JA7827AA	IIIb	IIIb	-	73836
16822	SAL_JA7831AA	IIIb	IIIb	-	73557
16075	SAL_JA7847AA	IIIb	IIIb	-	73517
16276	SAL_JA7854AA	IIIb	IIIb	-	73552
16819	SAL_JA8009AA	IIIb	IIIb	-	73642
16818	SAL_JA8030AA	IIIb	IIIb	-	73373
16279	SAL_JA8104AA	IIIb	IIIb	-	73320
16076	SAL_JA8421AA	IIIb	IIIb	-	73051
16826	SAL_JA9467AA	IIIb	IIIb	-	74342
16828	SAL_JA9983AA	IIIb	IIIb	-	75163
16831	SAL_KA0324AA	IIIb	IIIb	-	96807
16832	SAL_KA1188AA	IIIb	IIIb	-	76335
16835	SAL_KA1968AA	IIIb	IIIb	-	76918
16834	SAL_KA1973AA	IIIb	IIIb	-	76881
16838	SAL_KA2092AA	IIIb	IIIb	-	77124
16837	SAL_KA2096AA	IIIb	IIIb	-	77109
16071	SAL_KA3135AA	IIIb	IIIb	-	77750
16844	SAL_KA3267AA	IIIb	IIIb	-	78147
16845	SAL_KA4689AA	IIIb	IIIb	-	73672
16848	SAL_KA5642AA	IIIb	IIIb	-	80075
16849	SAL_KA5808AA	IIIb	IIIb	-	80361
16853	SAL_KA6433AA	IIIb	IIIb	-	82343
16851	SAL_KA6990AA	IIIb	IIIb	-	80843
16854	SAL_KA7134AA	IIIb	IIIb	-	81439
16862	SAL_KA8207AA	IIIb	IIIb	-	92745
16861	SAL_KA8229AA	IIIb	IIIb	-	81601
16860	SAL_KA8261AA	IIIb	IIIb	-	82351
16866	SAL_KA9588AA	IIIb	IIIb	-	82952
16868	SAL_LA0409AA	IIIb	IIIb	-	83758
16874	SAL_LA4234AA	IIIb	IIIb	-	86624
16069	SAL_LA5600AA	IIIb	IIIb	-	88093
16884	SAL_LA6048AA	IIIb	IIIb	-	89764
16286	SAL_LA6074AA	IIIb	IIIb	-	42344
16882	SAL_LA6256AA	IIIb	IIIb	-	89218
16877	SAL_LA6703AA	IIIb	IIIb	-	97879
16289	SAL_MA1729AA	IIIb	IIIb	-	93415
16983	SAL_MA1745AA	IIIb	IIIb	-	93293
16068	SAL_MA1758AA	IIIb	IIIb	-	93596
16976	SAL_MA1778AA	IIIb	IIIb	-	93283
16051	SAL_MA1783AA	IIIb	IIIb	-	93311
16050	SAL_MA1802AA	IIIb	IIIb	-	93335
16968	SAL_MA1815AA	IIIb	IIIb	-	93423
16967	SAL_MA1818AA	IIIb	IIIb	-	93339
16292	SAL_MA1829AA	IIIb	IIIb	-	93594
16961	SAL_MA1842AA	IIIb	IIIb	-	93425
16293	SAL_MA1850AA	IIIb	IIIb	-	93231
16294	SAL_MA1859AA	IIIb	IIIb	-	93238
16063	SAL_MA1872AA	IIIb	IIIb	-	93638
16952	SAL_MA1885AA	IIIb	IIIb	-	93248
16296	SAL_MA1891AA	IIIb	IIIb	-	93260
16951	SAL_MA1893AA	IIIb	IIIb	-	93650
16297	SAL_MA1904AA	IIIb	IIIb	-	93494
16054	SAL_MA1909AA	IIIb	IIIb	-	93259
16947	SAL_MA1910AA	IIIb	IIIb	-	93255
16944	SAL_MA1924AA	IIIb	IIIb	-	93661
16943	SAL_MA1926AA	IIIb	IIIb	-	93191
16298	SAL_MA1933AA	IIIb	IIIb	-	93265
16053	SAL_MA1948AA	IIIb	IIIb	-	93266
16300	SAL_MA1952AA	IIIb	IIIb	-	93677
16301	SAL_MA1953AA	IIIb	IIIb	-	93191
16939	SAL_MA1959AA	IIIb	IIIb	-	93682
16938	SAL_MA1969AA	IIIb	IIIb	-	93339
16934	SAL_MA1990AA	IIIb	IIIb	-	93703

16933	SAL_MA1997AA	IIIb	IIIb	-	93707
16045	SAL_MA2001AA	IIIb	IIIb	-	93713
16931	SAL_MA2008AA	IIIb	IIIb	-	93719
16044	SAL_MA2015AA	IIIb	IIIb	-	93724
16930	SAL_MA2018AA	IIIb	IIIb	-	93726
16927	SAL_MA2034AA	IIIb	IIIb	-	93316
16302	SAL_MA2036AA	IIIb	IIIb	-	93325
16303	SAL_MA2038AA	IIIb	IIIb	-	93223
16043	SAL_MA2045AA	IIIb	IIIb	-	93728
16925	SAL_MA2048AA	IIIb	IIIb	-	93729
16061	SAL_MA2050AA	IIIb	IIIb	-	93323
16060	SAL_MA2062AA	IIIb	IIIb	-	93355
16304	SAL_MA2067AA	IIIb	IIIb	-	93349
16059	SAL_MA2071AA	IIIb	IIIb	-	93351
16916	SAL_MA2080AA	IIIb	IIIb	-	93358
16305	SAL_MA2083AA	IIIb	IIIb	-	93359
16306	SAL_MA2094AA	IIIb	IIIb	-	93177
16307	SAL_MA2102AA	IIIb	IIIb	-	93183
16912	SAL_MA2108AA	IIIb	IIIb	-	93381
16911	SAL_MA2110AA	IIIb	IIIb	-	93189
16910	SAL_MA2123AA	IIIb	IIIb	-	93746
16908	SAL_MA2130AA	IIIb	IIIb	-	93191
16906	SAL_MA2137AA	IIIb	IIIb	-	93152
16902	SAL_MA2156AA	IIIb	IIIb	-	93748
16057	SAL_MA2158AA	IIIb	IIIb	-	93386
16311	SAL_MA2165AA	IIIb	IIIb	-	93395
16901	SAL_MA2166AA	IIIb	IIIb	-	93396
16314	SAL_MA2190AA	IIIb	IIIb	-	93167
16899	SAL_MA2193AA	IIIb	IIIb	-	93213
16896	SAL_MA2199AA	IIIb	IIIb	-	93148
16073	SAL_MA2201AA	IIIb	IIIb	-	93169
16315	SAL_MA2208AA	IIIb	IIIb	-	93219
16893	SAL_MA2210AA	IIIb	IIIb	-	93152
16891	SAL_MA2213AA	IIIb	IIIb	-	93174
16890	SAL_MA2215AA	IIIb	IIIb	-	93173
16317	SAL_MA2223AA	IIIb	IIIb	-	93221
17019	SAL_MA2327AA	IIIb	IIIb	-	93834
16318	SAL_MA2333AA	IIIb	IIIb	-	93839
17018	SAL_MA2336AA	IIIb	IIIb	-	93843
16037	SAL_MA2343AA	IIIb	IIIb	-	93848
16036	SAL_MA2347AA	IIIb	IIIb	-	93850
17017	SAL_MA2348AA	IIIb	IIIb	-	93851
16035	SAL_MA2357AA	IIIb	IIIb	-	93872
17011	SAL_MA2372AA	IIIb	IIIb	-	93879
17009	SAL_MA2375AA	IIIb	IIIb	-	93881
17007	SAL_MA2380AA	IIIb	IIIb	-	93864
16320	SAL_MA2389AA	IIIb	IIIb	-	93798
17003	SAL_MA2394AA	IIIb	IIIb	-	93794
16321	SAL_MA2398AA	IIIb	IIIb	-	93771
16998	SAL_MA2408AA	IIIb	IIIb	-	93805
16042	SAL_MA2409AA	IIIb	IIIb	-	98191
17021	SAL_MA2622AA	IIIb	IIIb	-	94200
17020	SAL_MA2795AA	IIIb	IIIb	-	94129
17023	SAL_MA3376AA	IIIb	IIIb	-	94494
17039	SAL_MA3954AA	IIIb	IIIb	-	95083
16324	SAL_MA3955AA	IIIb	IIIb	-	95091
16325	SAL_MA3960AA	IIIb	IIIb	-	95055
17037	SAL_MA3962AA	IIIb	IIIb	-	93395
17034	SAL_MA3981AA	IIIb	IIIb	-	93682
17033	SAL_MA3982AA	IIIb	IIIb	-	95065
17030	SAL_MA4001AA	IIIb	IIIb	-	95071
16327	SAL_MA4013AA	IIIb	IIIb	-	95019
17028	SAL_MA4014AA	IIIb	IIIb	-	95020
17027	SAL_MA4018AA	IIIb	IIIb	-	95024
16030	SAL_MA4420AA	IIIb	IIIb	-	95222
17047	SAL_MA4426AA	IIIb	IIIb	-	95237

17043	SAL_MA4441AA	IIIb	IIIb	-	95238
16328	SAL_MA4452AA	IIIb	IIIb	-	95656
16029	SAL_MA5801AA	IIIb	IIIb	-	96845
16329	SAL_MA5802AA	IIIb	IIIb	-	96424
17050	SAL_MA5803AA	IIIb	IIIb	-	96847
17051	SAL_MA5804AA	IIIb	IIIb	-	96849
17052	SAL_MA5805AA	IIIb	IIIb	-	96867
17054	SAL_MA5809AA	IIIb	IIIb	-	96858
17055	SAL_MA5811AA	IIIb	IIIb	-	96860
16025	SAL_MA5814AA	IIIb	IIIb	-	96873
17056	SAL_MA5815AA	IIIb	IIIb	-	96875
16332	SAL_MA5819AA	IIIb	IIIb	-	96878
17058	SAL_MA5820AA	IIIb	IIIb	-	96881
17059	SAL_MA5821AA	IIIb	IIIb	-	96880
16333	SAL_MA5822AA	IIIb	IIIb	-	96879
17060	SAL_MA5826AA	IIIb	IIIb	-	96885
16022	SAL_MA5827AA	IIIb	IIIb	-	96886
17061	SAL_MA5828AA	IIIb	IIIb	-	96888
17062	SAL_MA5831AA	IIIb	IIIb	-	96889
16081	SAL_MA5835AA	IIIb	IIIb	-	96891
16018	SAL_MA5839AA	IIIb	IIIb	-	96895
17066	SAL_MA5843AA	IIIb	IIIb	-	96898
16016	SAL_MA5845AA	IIIb	IIIb	-	96901
17068	SAL_MA5848AA	IIIb	IIIb	-	96904
16014	SAL_MA5851AA	IIIb	IIIb	-	96909
17070	SAL_MA5855AA	IIIb	IIIb	-	96906
17072	SAL_MA5859AA	IIIb	IIIb	-	97074
17074	SAL_MA5863AA	IIIb	IIIb	-	96922
17075	SAL_MA5864AA	IIIb	IIIb	-	96923
17076	SAL_MA5865AA	IIIb	IIIb	-	97059
17077	SAL_MA5866AA	IIIb	IIIb	-	97060
16008	SAL_MA5867AA	IIIb	IIIb	-	97077
17078	SAL_MA5868AA	IIIb	IIIb	-	97062
17173	SAL_MA5890AA	IIIb	IIIb	-	93248
17081	SAL_MA5891AA	IIIb	IIIb	-	97090
16342	SAL_MA5904AA	IIIb	IIIb	-	97093
16077	SAL_MA5922AA	IIIb	IIIb	-	97108
16058	SAL_MA5933AA	IIIb	IIIb	-	93359
17107	SAL_MA5959AA	IIIb	IIIb	-	97175
17111	SAL_MA6039AA	IIIb	IIIb	-	96989
16010	SAL_MA6043AA	IIIb	IIIb	-	96980
15973	SAL_NA5026AA	IIIb	IIIb	-	105008
17132	SAL_NA5030AA	IIIb	IIIb	-	105016
17134	SAL_NA5033AA	IIIb	IIIb	-	105018
17140	SAL_NA5044AA	IIIb	IIIb	-	105031
16046	SAL_NA5053AA	IIIb	IIIb	-	93707
17144	SAL_NA5058AA	IIIb	IIIb	-	105038
17146	SAL_NA5061AA	IIIb	IIIb	-	105042
15965	SAL_NA5062AA	IIIb	IIIb	-	105043
16363	SAL_NA5063AA	IIIb	IIIb	-	105039
15964	SAL_NA5064AA	IIIb	IIIb	-	105044
16138	SAL_NA5067AA	IIIb	IIIb	-	105050
17149	SAL_NA5068AA	IIIb	IIIb	-	105048
17151	SAL_NA5070AA	IIIb	IIIb	-	105046
17152	SAL_NA5071AA	IIIb	IIIb	-	105051
17154	SAL_NA5073AA	IIIb	IIIb	-	105046
17155	SAL_NA5074AA	IIIb	IIIb	-	105055
17157	SAL_NA5076AA	IIIb	IIIb	-	105054
17158	SAL_NA5080AA	IIIb	IIIb	-	105058
15961	SAL_NA5082AA	IIIb	IIIb	-	105061
15962	SAL_NA5084AA	IIIb	IIIb	-	105060
15960	SAL_NA5087AA	IIIb	IIIb	-	105064
16366	SAL_OA0437AA	IIIb	IIIb	-	109498
17165	SAL_OA0438AA	IIIb	IIIb	-	109499
16367	SAL_OA0439AA	IIIb	IIIb	-	109500
17166	SAL_OA0440AA	IIIb	IIIb	-	109504

17170	SAL_OA0447AA	IIIb	IIIb	-	109523	
17175	SAL_OA1860AA	IIIb	IIIb	-	110612	
16523	SAL_CA1297AA	IV	IV	-	29061	Yes
11990	SAL_CA5635AA	IV	IV	-	25526	Yes
16585	SAL_GA2049AA	IV	IV	-	43644	Yes
12667	SAL_GA3743AA	IV	IV	-	42360	Yes
16923	SAL_MA2052AA	IV	IV	-	93324	Yes
17048	SAL_MA4412AA	IV	IV	-	95195	Yes
16356	SAL_NA5018AA	IV	IV	-	105005	Yes
13358	SAL_BA1448AA	IV	IV	-	36412	
13464	SAL_BA1504AA	IV	IV	-	36368	
13462	SAL_BA1507AA	IV	IV	-	36365	
13461	SAL_BA1877AA	IV	IV	-	36059	
13472	SAL_BA2638AA	IV	IV	-	35440	
12954	SAL_BA3554AA	IV	IV	-	34716	
12971	SAL_BA5427AA	IV	IV	-	33406	
13596	SAL_BA6798AA	IV	IV	-	32314	
16427	SAL_BA7497AA	IV	IV	-	31803	
16430	SAL_BA7679AA	IV	IV	-	31710	
16434	SAL_BA7966AA	IV	IV	-	31536	
16436	SAL_BA8460AA	IV	IV	-	31232	
16437	SAL_BA8464AA	IV	IV	-	31229	
16438	SAL_BA8663AA	IV	IV	-	31062	
16520	SAL_BA8671AA	IV	IV	-	31056	
14923	SAL_BA8712AA	IV	IV	-	31022	
11976	SAL_BA8771AA	IV	IV	-	30971	
16439	SAL_BA8856AA	IV	IV	-	30904	
16173	SAL_BA8890AA	IV	IV	-	30876	
16522	SAL_CA0853AA	IV	IV	-	29298	
16413	SAL_CA1248AA	IV	IV	-	29087	
16461	SAL_CA2888AA	IV	IV	-	27884	
16462	SAL_CA3044AA	IV	IV	-	27754	
16524	SAL_CA3149AA	IV	IV	-	27666	
16465	SAL_CA3219AA	IV	IV	-	27605	
16468	SAL_CA3481AA	IV	IV	-	27378	
16471	SAL_CA4035AA	IV	IV	-	26876	
16472	SAL_CA4250AA	IV	IV	-	26693	
16474	SAL_CA4276AA	IV	IV	-	26671	
16479	SAL_CA4665AA	IV	IV	-	26336	
11796	SAL_CA4762AA	IV	IV	-	26252	
11989	SAL_CA4893AA	IV	IV	-	26137	
16485	SAL_CA5715AA	IV	IV	-	25458	
16487	SAL_CA5796AA	IV	IV	-	25392	
16491	SAL_CA6144AA	IV	IV	-	25089	
12395	SAL_CA7023AA	IV	IV	-	24297	
13418	SAL_CA7281AA	IV	IV	-	24059	
13410	SAL_CA7292AA	IV	IV	-	24049	
13412	SAL_CA7310AA	IV	IV	-	24032	
13608	SAL_CA7928AA	IV	IV	-	23494	
13377	SAL_CA8022AA	IV	IV	-	23411	
13403	SAL_CA8026AA	IV	IV	-	23407	
13397	SAL_CA8573AA	IV	IV	-	22935	
13356	SAL_CA9005AA	IV	IV	-	22573	
16419	SAL_CA9673AA	IV	IV	-	22043	
14044	SAL_DA0065AA	IV	IV	-	21720	
14070	SAL_DA0088AA	IV	IV	-	21702	
14367	SAL_DA0492AA	IV	IV	-	21366	
14361	SAL_DA0535AA	IV	IV	-	21327	
14359	SAL_DA0645AA	IV	IV	-	21226	
14372	SAL_DA0809AA	IV	IV	-	21071	
14378	SAL_DA0820AA	IV	IV	-	21060	
14451	SAL_DA0869AA	IV	IV	-	21016	
14417	SAL_DA0942AA	IV	IV	-	20951	
14428	SAL_DA0967AA	IV	IV	-	20930	
14444	SAL_DA1091AA	IV	IV	-	20812	
14419	SAL_DA1115AA	IV	IV	-	96354	

14373	SAL_DA1191AA	IV	IV	-	20716
14354	SAL_DA1207AA	IV	IV	-	20703
14497	SAL_DA1277AA	IV	IV	-	20635
14476	SAL_DA1491AA	IV	IV	-	20443
13369	SAL_DA1631AA	IV	IV	-	96355
13371	SAL_DA1646AA	IV	IV	-	20318
14075	SAL_DA1666AA	IV	IV	-	20302
13669	SAL_DA2584AA	IV	IV	-	15701
13668	SAL_DA2589AA	IV	IV	-	19548
12930	SAL_DA3695AA	IV	IV	-	18775
12927	SAL_DA3737AA	IV	IV	-	18723
13295	SAL_DA3941AA	IV	IV	-	18557
16498	SAL_DA4130AA	IV	IV	-	96314
14195	SAL_DA4371AA	IV	IV	-	18196
12468	SAL_DA4560AA	IV	IV	-	18048
12482	SAL_DA4981AA	IV	IV	-	17722
12484	SAL_DA5011AA	IV	IV	-	17695
14017	SAL_DA5037AA	IV	IV	-	17681
14592	SAL_DA5139AA	IV	IV	-	17595
13696	SAL_DA5467AA	IV	IV	-	17305
13304	SAL_DA5996AA	IV	IV	-	16864
14809	SAL_DA6185AA	IV	IV	-	16700
14157	SAL_DA6260AA	IV	IV	-	16631
14311	SAL_DA7348AA	IV	IV	-	15724
14317	SAL_DA7371AA	IV	IV	-	15701
13887	SAL_DA8459AA	IV	IV	-	14791
14131	SAL_DA8523AA	IV	IV	-	14735
14134	SAL_DA8565AA	IV	IV	-	14696
14112	SAL_DA9350AA	IV	IV	-	14002
14026	SAL_DA9801AA	IV	IV	-	13600
14167	SAL_EA0068AA	IV	IV	-	13378
14927	SAL_EA0151AA	IV	IV	-	13301
14174	SAL_EA0393AA	IV	IV	-	13086
14097	SAL_EA0632AA	IV	IV	-	12882
16527	SAL_EA1361AA	IV	IV	-	12427
13522	SAL_EA2463AA	IV	IV	-	11557
13523	SAL_EA2465AA	IV	IV	-	11557
13382	SAL_EA6458AA	IV	IV	-	8256
16538	SAL_EA7233AA	IV	IV	-	7597
16539	SAL_EA7287AA	IV	IV	-	7546
16546	SAL_EA8688AA	IV	IV	-	6294
16554	SAL_FA1631AA	IV	IV	-	3913
13449	SAL_FA5858AA	IV	IV	-	328
13471	SAL_FA5952AA	IV	IV	-	246
16216	SAL_FA8439AA	IV	IV	-	47415
16140	SAL_FA8440AA	IV	IV	-	47414
16218	SAL_FA9459AA	IV	IV	-	45801
12661	SAL_GA1783AA	IV	IV	-	43913
12474	SAL_GA1784AA	IV	IV	-	43912
12606	SAL_GA1785AA	IV	IV	-	43911
16576	SAL_GA2470AA	IV	IV	-	43207
16575	SAL_GA2472AA	IV	IV	-	43205
12724	SAL_GA2488AA	IV	IV	-	43192
12404	SAL_GA2494AA	IV	IV	-	43187
12749	SAL_GA2503AA	IV	IV	-	43178
12746	SAL_GA2504AA	IV	IV	-	43177
12525	SAL_GA2509AA	IV	IV	-	43172
12510	SAL_GA2513AA	IV	IV	-	43168
12434	SAL_GA2518AA	IV	IV	-	43163
12423	SAL_GA2521AA	IV	IV	-	43160
12736	SAL_GA2528AA	IV	IV	-	43152
12728	SAL_GA2530AA	IV	IV	-	43150
12701	SAL_GA2532AA	IV	IV	-	43149
12408	SAL_GA2540AA	IV	IV	-	43141
12745	SAL_GA2550AA	IV	IV	-	43131
12742	SAL_GA2553AA	IV	IV	-	43128

12529	SAL_GA2554AA	IV	IV	-	43127
12521	SAL_GA2556AA	IV	IV	-	43125
12505	SAL_GA2560AA	IV	IV	-	43121
12422	SAL_GA2567AA	IV	IV	-	42120
12740	SAL_GA2631AA	IV	IV	-	42055
12651	SAL_GA3279AA	IV	IV	-	42742
12654	SAL_GA3280AA	IV	IV	-	42736
12611	SAL_GA3281AA	IV	IV	-	42741
12494	SAL_GA3731AA	IV	IV	-	42369
16226	SAL_GA3733AA	IV	IV	-	44248
12659	SAL_GA3734AA	IV	IV	-	42367
12647	SAL_GA3735AA	IV	IV	-	42366
16227	SAL_GA3736AA	IV	IV	-	44247
12473	SAL_GA3737AA	IV	IV	-	42365
12618	SAL_GA3738AA	IV	IV	-	42364
12602	SAL_GA3739AA	IV	IV	-	42363
12662	SAL_GA3740AA	IV	IV	-	42362
16571	SAL_GA3741AA	IV	IV	-	42362
12696	SAL_GA3742AA	IV	IV	-	42361
12668	SAL_GA3745AA	IV	IV	-	42358
12462	SAL_GA3746AA	IV	IV	-	42356
12495	SAL_GA3747AA	IV	IV	-	42355
16231	SAL_GA3864AA	IV	IV	-	44233
12846	SAL_GA3865AA	IV	IV	-	42267
12845	SAL_GA3866AA	IV	IV	-	42266
712	SAL_GA4312AA	IV	IV	-	58194
16606	SAL_GA7857AA	IV	IV	-	48979
16607	SAL_GA8573AA	IV	IV	-	49511
16608	SAL_GA8656AA	IV	IV	-	96485
16099	SAL_GA8754AA	IV	IV	-	49633
16613	SAL_GA9263AA	IV	IV	-	50051
16624	SAL_HA1544AA	IV	IV	-	51940
16623	SAL_HA1619AA	IV	IV	-	51683
16626	SAL_HA2811AA	IV	IV	-	96456
16642	SAL_HA5522AA	IV	IV	-	54457
16641	SAL_HA5524AA	IV	IV	-	54459
16637	SAL_HA5525AA	IV	IV	-	54440
16638	SAL_HA5526AA	IV	IV	-	54452
16635	SAL_HA5530AA	IV	IV	-	54421
16639	SAL_HA5531AA	IV	IV	-	54456
16649	SAL_HA5559AA	IV	IV	-	54818
16646	SAL_HA5565AA	IV	IV	-	54821
16645	SAL_HA5567AA	IV	IV	-	54823
16656	SAL_HA7227AA	IV	IV	-	96446
16662	SAL_HA8189AA	IV	IV	-	57117
16391	SAL_HA8832AA	IV	IV	-	58017
16402	SAL_HA8856AA	IV	IV	-	58072
16673	SAL_IA0573AA	IV	IV	-	59578
16682	SAL_IA2615AA	IV	IV	-	61829
16685	SAL_IA5017AA	IV	IV	-	63031
16687	SAL_IA5784AA	IV	IV	-	59744
16689	SAL_IA6232AA	IV	IV	-	63959
16692	SAL_IA7025AA	IV	IV	-	64258
16261	SAL_IA7368AA	IV	IV	-	64794
16262	SAL_IA7370AA	IV	IV	-	64803
16699	SAL_IA8160AA	IV	IV	-	65315
16707	SAL_IA8670AA	IV	IV	-	65844
16706	SAL_IA8701AA	IV	IV	-	65802
16703	SAL_IA8800AA	IV	IV	-	96537
16263	SAL_IA8884AA	IV	IV	-	65682
16709	SAL_IA9155AA	IV	IV	-	66408
16710	SAL_IA9206AA	IV	IV	-	66440
16713	SAL_IA9909AA	IV	IV	-	66890
16716	SAL_JA0157AA	IV	IV	-	67071
16715	SAL_JA0174AA	IV	IV	-	67000
16718	SAL_JA0318AA	IV	IV	-	67165

16721	SAL_JA1462AA	IV	IV	-	67480
16734	SAL_JA3319AA	IV	IV	-	69574
16266	SAL_JA3618AA	IV	IV	-	69917
16737	SAL_JA3776AA	IV	IV	-	70329
16736	SAL_JA3834AA	IV	IV	-	70320
16738	SAL_JA4397AA	IV	IV	-	70497
16085	SAL_JA4421AA	IV	IV	-	70551
16747	SAL_JA4495AA	IV	IV	-	70588
16748	SAL_JA4918AA	IV	IV	-	70935
16761	SAL_JA5205AA	IV	IV	-	71080
16759	SAL_JA5209AA	IV	IV	-	71120
16800	SAL_JA6194AA	IV	IV	-	96649
16796	SAL_JA6356AA	IV	IV	-	71338
16795	SAL_JA6362AA	IV	IV	-	71326
16788	SAL_JA6525AA	IV	IV	-	71194
16785	SAL_JA6549AA	IV	IV	-	71185
16781	SAL_JA6560AA	IV	IV	-	71239
16775	SAL_JA6579AA	IV	IV	-	71202
16772	SAL_JA6583AA	IV	IV	-	71126
16816	SAL_JA7285AA	IV	IV	-	72983
16829	SAL_KA0577AA	IV	IV	-	96813
16833	SAL_KA0917AA	IV	IV	-	76444
16836	SAL_KA1868AA	IV	IV	-	76997
16842	SAL_KA2419AA	IV	IV	-	97722
16852	SAL_KA6765AA	IV	IV	-	80954
16855	SAL_KA7601AA	IV	IV	-	97794
16864	SAL_KA8527AA	IV	IV	-	92676
16863	SAL_KA8577AA	IV	IV	-	92688
16867	SAL_KA9357AA	IV	IV	-	83173
16865	SAL_KA9641AA	IV	IV	-	82899
16873	SAL_LA4316AA	IV	IV	-	97854
16872	SAL_LA4327AA	IV	IV	-	86555
16875	SAL_LA4390AA	IV	IV	-	86849
16881	SAL_LA6300AA	IV	IV	-	89129
16287	SAL_LA6303AA	IV	IV	-	88774
16879	SAL_LA6383AA	IV	IV	-	88733
16067	SAL_LA8171AA	IV	IV	-	90681
16888	SAL_LA9320AA	IV	IV	-	91653
16989	SAL_MA1636AA	IV	IV	-	93470
16290	SAL_MA1738AA	IV	IV	-	93290
16048	SAL_MA1741AA	IV	IV	-	93418
16982	SAL_MA1748AA	IV	IV	-	93294
16981	SAL_MA1749AA	IV	IV	-	93295
16094	SAL_MA1750AA	IV	IV	-	93288
16980	SAL_MA1753AA	IV	IV	-	93419
16052	SAL_MA1788AA	IV	IV	-	93309
16973	SAL_MA1793AA	IV	IV	-	93310
16970	SAL_MA1807AA	IV	IV	-	93492
16969	SAL_MA1811AA	IV	IV	-	93337
16962	SAL_MA1839AA	IV	IV	-	93400
16295	SAL_MA1875AA	IV	IV	-	93640
16047	SAL_MA1876AA	IV	IV	-	93641
16950	SAL_MA1895AA	IV	IV	-	93651
16949	SAL_MA1900AA	IV	IV	-	93253
16948	SAL_MA1907AA	IV	IV	-	93254
16049	SAL_MA1915AA	IV	IV	-	93262
16946	SAL_MA1919AA	IV	IV	-	93656
16055	SAL_MA1928AA	IV	IV	-	93663
16937	SAL_MA1970AA	IV	IV	-	93695
16932	SAL_MA1999AA	IV	IV	-	93708
16928	SAL_MA2028AA	IV	IV	-	93315
16920	SAL_MA2060AA	IV	IV	-	93745
16919	SAL_MA2069AA	IV	IV	-	93354
16918	SAL_MA2074AA	IV	IV	-	93363
16914	SAL_MA2085AA	IV	IV	-	93361
16308	SAL_MA2109AA	IV	IV	-	93750

16062	SAL_MA2113AA	IV	IV	-	93186	
16909	SAL_MA2124AA	IV	IV	-	93187	
16310	SAL_MA2142AA	IV	IV	-	93197	
16903	SAL_MA2155AA	IV	IV	-	93146	
16056	SAL_MA2161AA	IV	IV	-	93388	
16900	SAL_MA2168AA	IV	IV	-	93392	
16312	SAL_MA2175AA	IV	IV	-	93754	
16313	SAL_MA2183AA	IV	IV	-	93209	
16897	SAL_MA2196AA	IV	IV	-	93215	
16065	SAL_MA2204AA	IV	IV	-	93151	
16895	SAL_MA2205AA	IV	IV	-	93216	
16892	SAL_MA2211AA	IV	IV	-	93153	
16064	SAL_MA2218AA	IV	IV	-	93157	
16038	SAL_MA2335AA	IV	IV	-	93840	
16319	SAL_MA2337AA	IV	IV	-	93844	
17014	SAL_MA2362AA	IV	IV	-	93877	
17012	SAL_MA2371AA	IV	IV	-	93862	
17008	SAL_MA2379AA	IV	IV	-	93863	
17005	SAL_MA2388AA	IV	IV	-	93791	
17002	SAL_MA2396AA	IV	IV	-	93806	
17001	SAL_MA2399AA	IV	IV	-	93796	
16997	SAL_MA2414AA	IV	IV	-	93800	
16996	SAL_MA2416AA	IV	IV	-	93810	
16322	SAL_MA2418AA	IV	IV	-	93803	
16040	SAL_MA2424AA	IV	IV	-	93804	
16323	SAL_MA2431AA	IV	IV	-	93783	
16993	SAL_MA2439AA	IV	IV	-	93762	
16992	SAL_MA2440AA	IV	IV	-	93761	
16991	SAL_MA2450AA	IV	IV	-	31232	
16033	SAL_MA3952AA	IV	IV	-	95087	
17032	SAL_MA3984AA	IV	IV	-	95066	
17031	SAL_MA3988AA	IV	IV	-	95069	
16034	SAL_MA4023AA	IV	IV	-	95032	
16032	SAL_MA4419AA	IV	IV	-	95197	
17045	SAL_MA4436AA	IV	IV	-	95230	
17041	SAL_MA4447AA	IV	IV	-	95204	
17082	SAL_MA5892AA	IV	IV	-	97087	
17119	SAL_NA1644AA	IV	IV	-	102080	
15978	SAL_NA5005AA	IV	IV	-	104992	
16354	SAL_NA5007AA	IV	IV	-	104995	
15976	SAL_NA5010AA	IV	IV	-	104997	
16355	SAL_NA5015AA	IV	IV	-	105002	
17126	SAL_NA5016AA	IV	IV	-	104999	
17127	SAL_NA5017AA	IV	IV	-	105000	
17128	SAL_NA5019AA	IV	IV	-	105003	
16357	SAL_NA5020AA	IV	IV	-	105007	
15974	SAL_NA5021AA	IV	IV	-	105006	
15970	SAL_NA5029AA	IV	IV	-	105015	
17136	SAL_NA5037AA	IV	IV	-	105022	
16360	SAL_NA5041AA	IV	IV	-	105029	
15968	SAL_NA5042AA	IV	IV	-	105028	
15966	SAL_NA5049AA	IV	IV	-	105036	
17145	SAL_NA5060AA	IV	IV	-	105040	
17150	SAL_NA5069AA	IV	IV	-	105049	
17153	SAL_NA5072AA	IV	IV	-	105052	
15977	SAL_NA5077AA	IV	IV	-	105053	
16139	SAL_NA5079AA	IV	IV	-	105056	
16365	SAL_NA5083AA	IV	IV	-	105062	
15959	SAL_NA5085AA	IV	IV	-	105065	
15958	SAL_NA5088AA	IV	IV	-	105066	
17164	SAL_NA7697AA	IV	IV	-	107343	
17163	SAL_NA7698AA	IV	IV	-	107344	
17161	SAL_NA7700AA	IV	IV	-	107280	
17168	SAL_OA0443AA	IV	IV	-	109509	
16141	SAL_BA7509AA	novel_subspecies_A	novel_subspecies_A	-	31793	Yes
16142	SAL_DA5013AA	novel_subspecies_A	novel_subspecies_A	-	17692	Yes

16111	SAL_JA5200AA	novel_subspecies_A	novel_subspecies_A	-	96639	Yes
13568	SAL_BA2000AA		novel_subspecies_B	-	35955	Yes
16658	SAL_HA7736AA		novel_subspecies_B	-	57055	Yes
16664	SAL_HA8246AA		novel_subspecies_B	-	57487	Yes
16756	SAL_JA5214AA	novel_subspecies_B	novel_subspecies_B	-	71074	Yes
16156	SAL_JA6552AA	novel_subspecies_B	novel_subspecies_B	-	71154	Yes
15996	SAL_MA5929AA	II	novel_subspecies_B	-	97113	Yes
17115	SAL_MA8213AA		novel_subspecies_B	-	99848	Yes
15280	SAL_BA1880AA	II	novel_subspecies_B	-	36056	
16155	SAL_CA7278AA		novel_subspecies_B	-	24061	
13316	SAL_DA1882AA	novel_subspecies_B	novel_subspecies_B	-	20120	
16143	SAL_DA4558AA		novel_subspecies_B	-	18050	
16158	SAL_DA4993AA	novel_subspecies_B	novel_subspecies_B	-	96389	
16154	SAL_DA5014AA		novel_subspecies_B	-	17691	
16660	SAL_HA7731AA		novel_subspecies_B	-	57051	
16659	SAL_HA7735AA		novel_subspecies_B	-	57049	
16663	SAL_HA8266AA		novel_subspecies_B	-	57459	
16669	SAL_HA8764AA		novel_subspecies_B	-	58009	
16157	SAL_HA8765AA	novel_subspecies_B	novel_subspecies_B	-	58010	
16668	SAL_HA8766AA		novel_subspecies_B	-	58010	
16667	SAL_HA8768AA		novel_subspecies_B	-	57049	
16666	SAL_HA8769AA		novel_subspecies_B	-	58003	
16671	SAL_HA9179AA		novel_subspecies_B	-	58492	
16670	SAL_HA9190AA		novel_subspecies_B	-	58451	
16679	SAL_IA0400AA		novel_subspecies_B	-	59732	
16677	SAL_IA0406AA		novel_subspecies_B	-	59664	
16674	SAL_IA0448AA		novel_subspecies_B	-	59660	
16688	SAL_IA5780AA		novel_subspecies_B	-	59732	
16697	SAL_IA7579AA		novel_subspecies_B	-	65027	
16696	SAL_IA7593AA		novel_subspecies_B	-	65027	
16720	SAL_JA1594AA		novel_subspecies_B	-	67398	
16730	SAL_JA1636AA		novel_subspecies_B	-	68202	
16749	SAL_JA5110AA		novel_subspecies_B	-	57459	
16808	SAL_JA5334AA		novel_subspecies_B	-	72354	
16791	SAL_JA6519AA		novel_subspecies_B	-	71191	
16858	SAL_KA8153AA		novel_subspecies_B	-	82026	
16859	SAL_KA8181AA	novel_subspecies_B	novel_subspecies_B	-	82023	
17024	SAL_MA3275AA		novel_subspecies_B	-	96265	
17095	SAL_MA5932AA	II	novel_subspecies_B	-	97113	
16153	SAL_MA6203AA		novel_subspecies_B	-	97523	
15979	SAL_MA6204AA		novel_subspecies_B	-	97578	
17114	SAL_MA7539AA		novel_subspecies_B	-	98766	
17117	SAL_MA8202AA		novel_subspecies_B	-	99879	
17116	SAL_MA8212AA		novel_subspecies_B	-	99926	
17118	SAL_NA1705AA		novel_subspecies_B	-	102087	
17120	SAL_NA2426AA		novel_subspecies_B	-	102694	
17121	SAL_NA2993AA		novel_subspecies_B	-	103001	
17187	SAL_NA5898AA		novel_subspecies_B	-	105914	
17188	SAL_NA8822AA		novel_subspecies_B	-	108456	
16521	SAL_CA0801AA	novel_subspecies_C	novel_subspecies_C	-	96414	Yes
9629	SAL_DA1087AA	novel_subspecies_C	novel_subspecies_C	-	20816	Yes
16159	SAL_DA4564AA	novel_subspecies_C	novel_subspecies_C	-	96345	Yes
12478	SAL_DA4990AA	novel_subspecies_C	novel_subspecies_C	-	96325	Yes
12469	SAL_DA5012AA	novel_subspecies_C	novel_subspecies_C	-	17693	Yes
16686	SAL_IA5387AA		novel_subspecies_C	-	63150	Yes
16161	SAL_NA5014AA	II	novel_subspecies_C	-	105004	Yes
16145	SAL_DA4996AA	novel_subspecies_C	novel_subspecies_C	-	17708	
12856	SAL_GA3548AA	II	novel_subspecies_C	-	42515	
16080	SAL_JA5227AA	novel_subspecies_C	novel_subspecies_C	-	71056	
16160	SAL_MA5883AA	II	novel_subspecies_C	-	97081	
17108	SAL_MA5961AA	II	novel_subspecies_C	-	97173	
15989	SAL_MA5962AA	II	novel_subspecies_C	-	97176	
17112	SAL_MA6041AA	II	novel_subspecies_C	-	96982	
17129	SAL_NA5022AA	II	novel_subspecies_C	-	105010	
17133	SAL_NA5031AA	II	novel_subspecies_C	-	105020	
15967	SAL_NA5047AA	II	novel_subspecies_C	-	105033	

16146	SAL_OA0444AA	II	novel_subspecies_C	-	109502	
16162	SAL_OA0448AA	II	novel_subspecies_C	-	109519	
16415	SAL_CA0664AA	*S.bongori*	*S.bongori*	-	29458	Yes
13799	SAL_FA0906AA	*S.bongori*	*S.bongori*	-	4531	Yes
16570	SAL_GA3861AA	*S.bongori*	*S.bongori*	-	42270	Yes
16095	SAL_JA4400AA	*S.bongori*	*S.bongori*	-	70496	Yes
16090	SAL_JA4409AA	*S.bongori*	*S.bongori*	-	70540	Yes
16267	SAL_JA5213AA	*S.bongori*	*S.bongori*	-	96641	Yes
16362	SAL_NA5050AA	*S.bongori*	*S.bongori*	-	105034	Yes
12139	SAL_BA6694AA	*S.bongori*	*S.bongori*	-	32409	
12049	SAL_BA8870AA	*S.bongori*	*S.bongori*	-	30893	
15140	SAL_CA0623AA	*S.bongori*	*S.bongori*	-	29492	
16448	SAL_CA0639AA	*S.bongori*	*S.bongori*	-	29477	
12887	SAL_CA0643AA	*S.bongori*	*S.bongori*	-	29473	
16449	SAL_CA0655AA	*S.bongori*	*S.bongori*	-	29465	
16493	SAL_CA6389AA	*S.bongori*	*S.bongori*	-	24869	
13306	SAL_DA2028AA	*S.bongori*	*S.bongori*	-	20003	
16499	SAL_DA4461AA	*S.bongori*	*S.bongori*	-	18136	
16500	SAL_DA4462AA	*S.bongori*	*S.bongori*	-	96392	
16501	SAL_DA4467AA	*S.bongori*	*S.bongori*	-	96361	
16502	SAL_DA4470AA	*S.bongori*	*S.bongori*	-	18127	
16503	SAL_DA4473AA	*S.bongori*	*S.bongori*	-	18124	
13189	SAL_DA5347AA	*S.bongori*	*S.bongori*	-	17409	
13269	SAL_DA6976AA	*S.bongori*	*S.bongori*	-	16031	
16206	SAL_DA7766AA	*S.bongori*	*S.bongori*	-	15421	
16548	SAL_EA9719AA	*S.bongori*	*S.bongori*	-	96387	
16549	SAL_EA9763AA	*S.bongori*	*S.bongori*	-	5479	
14904	SAL_EA9772AA	*S.bongori*	*S.bongori*	-	5470	
16587	SAL_GA1781AA	*S.bongori*	*S.bongori*	-	43915	
16221	SAL_GA3147AA	*S.bongori*	*S.bongori*	-	42866	
16100	SAL_GA3148AA	*S.bongori*	*S.bongori*	-	96455	
16222	SAL_GA3149AA	*S.bongori*	*S.bongori*	-	42864	
16223	SAL_GA3278AA	*S.bongori*	*S.bongori*	-	42737	
16109	SAL_GA3655AA	*S.bongori*	*S.bongori*	-	42433	
16224	SAL_GA3656AA	*S.bongori*	*S.bongori*	-	42432	
16225	SAL_GA3727AA	*S.bongori*	*S.bongori*	-	42372	
16110	SAL_GA3728AA	*S.bongori*	*S.bongori*	-	42371	
16572	SAL_GA3729AA	*S.bongori*	*S.bongori*	-	42370	
16569	SAL_GA3862AA	*S.bongori*	*S.bongori*	-	42269	
16614	SAL_GA9240AA	*S.bongori*	*S.bongori*	-	50067	
16392	SAL_HA8836AA	*S.bongori*	*S.bongori*	-	58092	
16265	SAL_JA0617AA	*S.bongori*	*S.bongori*	-	96577	
16765	SAL_JA5197AA	*S.bongori*	*S.bongori*	-	96640	
16762	SAL_JA5204AA	*S.bongori*	*S.bongori*	-	96661	
16269	SAL_JA5230AA	*S.bongori*	*S.bongori*	-	96637	
16274	SAL_JA6559AA	*S.bongori*	*S.bongori*	-	96670	
16771	SAL_JA6585AA	*S.bongori*	*S.bongori*	-	96644	
16814	SAL_JA6972AA	*S.bongori*	*S.bongori*	-	96701	
16812	SAL_JA6978AA	*S.bongori*	*S.bongori*	-	96674	
16021	SAL_MA5830AA	*S.bongori*	*S.bongori*	-	96887	
17086	SAL_MA5908AA	*S.bongori*	*S.bongori*	-	97094	
16353	SAL_MA5991AA	*S.bongori*	*S.bongori*	-	97195	
15972	SAL_NA5025AA	*S.bongori*	*S.bongori*	-	105012	
16358	SAL_NA5027AA	*S.bongori*	*S.bongori*	-	105013	
15971	SAL_NA5028AA	*S.bongori*	*S.bongori*	-	105014	
17135	SAL_NA5034AA	*S.bongori*	*S.bongori*	-	105019	
17138	SAL_NA5039AA	*S.bongori*	*S.bongori*	-	105025	
17142	SAL_NA5048AA	*S.bongori*	*S.bongori*	-	105035	
13620	SAL_CA9757AA	VI	VI	-	21972	Yes
12496	SAL_DA4983AA	VI	VI	-	17720	Yes
12476	SAL_GA1719AA	VI	VI	-	43983	Yes
16220	SAL_GA2459AA	VI	VI	-	43215	Yes
12601	SAL_GA3654AA	VI	VI	-	42434	Yes
16393	SAL_HA8831AA	VI	VI	-	58096	Yes
16767	SAL_JA5195AA	VI	VI	-	71224	Yes
11463	SAL_CA1579AA	VI	VI	-	28884	

14197	SAL_CA7665AA	VI	VI	-	23725	
14055	SAL_DA3967AA	VI	VI	-	18535	
12479	SAL_DA4559AA	VI	VI	-	96391	
16152	SAL_EA2899AA	VI	VI	-	11201	
12645	SAL_GA1721AA	VI	VI	-	43971	
12633	SAL_GA1722AA	VI	VI	-	43969	
16151	SAL_GA1810AA	VI	VI	-	43888	
16579	SAL_GA2463AA	VI	VI	-	43213	
16578	SAL_GA2464AA	VI	VI	-	43212	
12470	SAL_GA3285AA	VI	VI	-	42732	
12603	SAL_GA3653AA	VI	VI	-	42435	
12605	SAL_GA3660AA	VI	VI	-	42428	
12750	SAL_GA3661AA	VI	VI	-	42427	
12848	SAL_GA3860AA	VI	VI	-	28884	
16092	SAL_JA4408AA	VI	VI	-	70538	
16766	SAL_JA5196AA	VI	VI	-	71218	
16268	SAL_JA5223AA	VI	VI	-	71058	
16827	SAL_KA0221AA	VI	VI	-	75048	
16964	SAL_MA1830AA	VI	VI	-	93461	
17137	SAL_NA5038AA	VI	VI	-	105026	
15147	SAL_DA5008AA	VII	VII	-	17697	Yes
13892	SAL_DA8458AA	VII	VII	-	96401	Yes
16552	SAL_FA1255AA	VII	VII	-	96504	Yes
12609	SAL_GA1787AA	VII	VII	-	43909	Yes
16163	SAL_JA5192AA	VII	VII	-	71284	Yes
16164	SAL_JA6574AA	VII	VII	-	71132	Yes
16108	SAL_MA2040AA	IV	VII	-	93222	Yes
13894	SAL_DA8447AA		VII	-	14800	
17178	SAL_DA8710AA		VII	-	14568	
17179	SAL_GA1770AA	I	VII	-	43909	
16577	SAL_GA2467AA	VII	VII	-	96474	
12664	SAL_GA3744AA	VII	VII	-	42359	
17180	SAL_JA0205AA		VII	-	66990	
17181	SAL_JA6699AA		VII	-	72527	
17182	SAL_KA0784AA		VII	-	75426	
17183	SAL_KA5849AA		VII	-	80299	
17184	SAL_KA6946AA		VII	-	80763	
16883	SAL_LA6072AA	VII	VII	-	89532	
16885	SAL_LA7583AA	VII	VII	-	89959	
17185	SAL_NA5372AA		VII	-	105228	
17186	SAL_NA5383AA		VII	-	105228	
17162	SAL_NA7699AA	IV	VII	-	107306	
