# Supplementary material for: A proposed core genome scheme for analyses of the Salmonella genus
Source: Genomics. 2020 Jan;112(1):371–8. doi: 10.1016/j.ygeno.2019.02.016 (PMC6978875; doi:10.1016/j.ygeno.2019.02.016)
Supplement: Supplementary Table S3 — Table showing the paralogous core genome loci. These loci were removed from the core genome schemes in order to perform the Structure analyses, due to the presence of paralogs in one or more isolates. The schemes from which they were removed was also stated. [file mmc3.pdf]

loci	analysis_removed_from
**STMMW_36391**	Enterobase_Scheme&Genus_Scheme
**STMMW_00801**	Enterobase_Scheme&Genus_Scheme
**STMMW_17531**	Enterobase_Scheme&Genus_Scheme
**STMMW_23761**	Enterobase_Scheme&Genus_Scheme
**STMMW_44771**	Enterobase_Scheme&Genus_Scheme
**STMMW_05331**	Enterobase_Scheme&Genus_Scheme
**STMMW_35531**	Enterobase_Scheme&Genus_Scheme
**STMMW_13231**	Enterobase_Scheme&Genus_Scheme
**STMMW_24481**	Enterobase_Scheme&Genus_Scheme
**STMMW_37551**	Enterobase_Scheme
**STMMW_18441**	Enterobase_Scheme
