# Supplementary material for: A proposed core genome scheme for analyses of the Salmonella genus
Source: Genomics. 2020 Jan;112(1):371–8. doi: 10.1016/j.ygeno.2019.02.016 (PMC6978875; doi:10.1016/j.ygeno.2019.02.016)
Supplement: Supplementary Table S4 — Table showing the loci removed for each subspecies. This table showed that many of these loci were missing in over 2% of multiple subspecies. The removal of some but not all of these loci would allow for the creation of further schemes; for example, the removal of all loci accept those specific to S. bongori would allow for the creation of a S. enterica species core scheme. [file mmc4.pdf]

Locus	Count_Missing	Percent_Missing	Subspecies
STMMW_06181	6	2.23880597	II
STMMW_11061	6	2.23880597	II
STMMW_11071	6	2.23880597	II
STMMW_14061	6	2.23880597	II
STMMW_14071	6	2.23880597	II
STMMW_14081	6	2.23880597	II
STMMW_14091	6	2.23880597	II
STMMW_14101	6	2.23880597	II
STMMW_14241	6	2.23880597	II
STMMW_14251	6	2.23880597	II
STMMW_14261	6	2.23880597	II
STMMW_44481	6	2.23880597	II
STMMW_12521	6	2.23880597	II
STMMW_12541	6	2.23880597	II
STMMW_12562	6	2.23880597	II
STMMW_15321	6	2.23880597	II
STMMW_15331	6	2.23880597	II
STMMW_15341	6	2.23880597	II
STMMW_15351	6	2.23880597	II
STMMW_15361	6	2.23880597	II
STMMW_15371	6	2.23880597	II
STMMW_18441	6	2.23880597	II
STMMW_28291	6	2.23880597	II
STMMW_28371	6	2.23880597	II
STMMW_34021	6	2.23880597	II
STMMW_14051	7	2.611940299	II
STMMW_14111	7	2.611940299	II
STMMW_14121	7	2.611940299	II
STM1092	7	2.611940299	II
STMMW_12561	7	2.611940299	II
STMMW_28381	7	2.611940299	II
STMMW_28391	7	2.611940299	II
STMMW_28411	7	2.611940299	II
STMMW_37041	7	2.611940299	II
STMMW_37051	7	2.611940299	II
STMMW_13981	8	2.985074627	II
STMMW_13991	8	2.985074627	II
STMMW_14001	8	2.985074627	II
STMMW_14021	8	2.985074627	II
STMMW_14031	8	2.985074627	II
STMMW_14041	8	2.985074627	II
STMMW_14131	8	2.985074627	II
STMMW_14141	8	2.985074627	II
STMMW_14151	8	2.985074627	II
STMMW_14161	8	2.985074627	II
STMMW_14171	8	2.985074627	II

STMMW_14181	8	2.985074627	II
STMMW_14191	8	2.985074627	II
STMMW_14201	8	2.985074627	II
STMMW_14211	8	2.985074627	II
STMMW_14221	8	2.985074627	II
STMMW_14231	8	2.985074627	II
STMMW_12551	8	2.985074627	II
STMMW_37061	8	2.985074627	II
STMMW_37071	8	2.985074627	II
STMMW_37081	8	2.985074627	II
STMMW_30841	9	3.358208955	II
STMMW_30851	9	3.358208955	II
STMMW_30861	9	3.358208955	II
STMMW_30871	9	3.358208955	II
STMMW_30881	9	3.358208955	II
STMMW_30891	9	3.358208955	II
STMMW_42651	11	4.104477612	II
STMMW_42641	12	4.47761194	II
STMMW_23611	29	10.94339623	II
STMMW_23621	29	10.94339623	II
STMMW_23641	29	10.94339623	II
STMMW_23651	29	10.94339623	II
STMMW_23661	29	10.94339623	II
STMMW_23671	29	10.94339623	II
STMMW_43331	56	21.13207547	II
STMMW_42081	66	24.90566038	II
STMMW_42091	66	24.90566038	II
STMMW_42101	66	24.90566038	II
STMMW_21691	118	44.52830189	II
STMMW_28741	128	48.30188679	II
STMMW_28751	128	48.30188679	II
STMMW_11031	176	66.41509434	II
STMMW_11021	179	67.54716981	II
STMMW_42501	221	83.39622642	II
STMMW_42101	8	2.640264026	IIIa
STMMW_42091	9	2.97029703	IIIa
STMMW_21301	9	2.97029703	IIIa
STMMW_21311	9	2.97029703	IIIa
STMMW_21331	9	2.97029703	IIIa
STMMW_21341	9	2.97029703	IIIa
STMMW_42081	10	3.300330033	IIIa
STMMW_22041	13	4.290429043	IIIa
STMMW_42651	15	4.95049505	IIIa
STMMW_42641	17	5.610561056	IIIa
STMMW_01621	21	6.930693069	IIIa
STMMW_07481	32	10.56105611	IIIa
STMMW_31491	32	10.56105611	IIIa

STMMW_06121	41	13.53135314	IIIa
STMMW_23211	76	25.08250825	IIIa
STMMW_37551	81	26.73267327	IIIa
STMMW_43841	89	29.37293729	IIIa
STMMW_13251	109	35.97359736	IIIa
STMMW_15801	111	36.63366337	IIIa
STMMW_06161	148	48.84488449	IIIa
STMMW_06171	148	48.84488449	IIIa
STMMW_06181	148	48.84488449	IIIa
STMMW_06201	148	48.84488449	IIIa
STMMW_01611	165	54.45544554	IIIa
STMMW_22081	175	57.75577558	IIIa
STMMW_22091	175	57.75577558	IIIa
STMMW_11491	184	60.72607261	IIIa
STMMW_11501	184	60.72607261	IIIa
STMMW_09341	217	71.61716172	IIIa
STMMW_09351	217	71.61716172	IIIa
STMMW_09361	217	71.61716172	IIIa
STMMW_00101	259	85.47854785	IIIa
STMMW_00091	260	85.80858086	IIIa
STMMW_44481	283	93.39933993	IIIa
STMMW_22191	284	93.72937294	IIIa
STMMW_22181	287	94.71947195	IIIa
STMMW_24261	293	96.69966997	IIIa
STM3012	299	98.67986799	IIIa
STMMW_42151	301	99.33993399	IIIa
STMMW_13741	302	99.669967	IIIa
STMMW_36521	302	99.669967	IIIa
STMMW_04911	303	100	IIIa
STMMW_05791	303	100	IIIa
STMMW_07491	303	100	IIIa
STMMW_07501	303	100	IIIa
STMMW_09971	303	100	IIIa
STMMW_12701	303	100	IIIa
STMMW_12931	303	100	IIIa
STMMW_14811	303	100	IIIa
STMMW_15771	303	100	IIIa
STMMW_15791	303	100	IIIa
STMMW_16131	303	100	IIIa
STMMW_16161	303	100	IIIa
STMMW_16991	303	100	IIIa
STMMW_19431	303	100	IIIa
STMMW_21731	303	100	IIIa
STMMW_21741	303	100	IIIa
STMMW_21751	303	100	IIIa
STMMW_23221	303	100	IIIa
STMMW_23231	303	100	IIIa

STMMW_25861	303	100	IIIa
STMMW_28761	303	100	IIIa
STMMW_28771	303	100	IIIa
STMMW_28791	303	100	IIIa
STMMW_31021	303	100	IIIa
STMMW_32331	303	100	IIIa
STMMW_34703	303	100	IIIa
STMMW_35301	303	100	IIIa
STMMW_35741	303	100	IIIa
STMMW_35751	303	100	IIIa
STMMW_35761	303	100	IIIa
STMMW_37561	303	100	IIIa
STMMW_37631	303	100	IIIa
STMMW_37641	303	100	IIIa
STMMW_37651	303	100	IIIa
STMMW_43861	303	100	IIIa
STMMW_43881	303	100	IIIa
STMMW_43891	303	100	IIIa
STMMW_43901	303	100	IIIa
STMMW_43921	303	100	IIIa
STMMW_44141	303	100	IIIa
STMMW_44541	303	100	IIIa
STMMW_44881	303	100	IIIa
STMMW_42101	9	2.122641509	IIIb
STMMW_21301	10	2.358490566	IIIb
STMMW_21311	10	2.358490566	IIIb
STMMW_21331	10	2.358490566	IIIb
STMMW_21341	10	2.358490566	IIIb
STMMW_27421	16	3.773584906	IIIb
STMMW_27431	16	3.773584906	IIIb
STMMW_27441	16	3.773584906	IIIb
STMMW_27411	17	4.009433962	IIIb
STMMW_42641	27	6.367924528	IIIb
STMMW_42651	27	6.367924528	IIIb
STMMW_01221	7	2.194357367	IV
STMMW_21301	12	3.761755486	IV
STMMW_21311	12	3.761755486	IV
STMMW_21331	12	3.761755486	IV
STMMW_18431	13	4.07523511	IV
STMMW_21341	14	4.388714734	IV
STMMW_44481	16	5.015673981	IV
STMMW_01491	20	6.269592476	IV
STMMW_20411	225	70.53291536	IV
STMMW_20501	267	83.69905956	IV
STMMW_04631	42	100	Novel_B
STMMW_06111	42	100	Novel_B
STMMW_06121	42	100	Novel_B

STMMW_06131	42	100	Novel_B
STMMW_06141	42	100	Novel_B
STMMW_06151	42	100	Novel_B
STMMW_06161	42	100	Novel_B
STMMW_06171	42	100	Novel_B
STMMW_06181	42	100	Novel_B
STMMW_08391	42	100	Novel_B
STMMW_08401	42	100	Novel_B
STMMW_08411	42	100	Novel_B
STMMW_08421	42	100	Novel_B
STMMW_08431	42	100	Novel_B
STMMW_11021	42	100	Novel_B
STMMW_16161	42	100	Novel_B
STMMW_16601	42	100	Novel_B
STMMW_16611	42	100	Novel_B
STMMW_21691	42	100	Novel_B
STMMW_24971	42	100	Novel_B
STMMW_33241	42	100	Novel_B
STMMW_23791	10	52.63157895	Novel_C
STMMW_23801	10	52.63157895	Novel_C
STMMW_23811	10	52.63157895	Novel_C
STMMW_23821	10	52.63157895	Novel_C
STMMW_23831	10	52.63157895	Novel_C
STMMW_16601	18	94.73684211	Novel_C
STMMW_16611	18	94.73684211	Novel_C
STMMW_28741	18	94.73684211	Novel_C
STMMW_28751	18	94.73684211	Novel_C
STMMW_30841	19	100	Novel_C
STMMW_30851	19	100	Novel_C
STMMW_30861	19	100	Novel_C
STMMW_30871	19	100	Novel_C
STMMW_30881	19	100	Novel_C
STMMW_30891	19	100	Novel_C
STMMW_32761	19	100	Novel_C
STMMW_44481	12	21.81818182	*S.bongori*
STMMW_11091	17	30.90909091	*S.bongori*
STMMW_11101	17	30.90909091	*S.bongori*
STMMW_11111	17	30.90909091	*S.bongori*
STMMW_11121	17	30.90909091	*S.bongori*
STMMW_11131	17	30.90909091	*S.bongori*
STMMW_11141	17	30.90909091	*S.bongori*
STMMW_11151	17	30.90909091	*S.bongori*
STMMW_11161	17	30.90909091	*S.bongori*
STMMW_11171	17	30.90909091	*S.bongori*
STMMW_11181	17	30.90909091	*S.bongori*
STMMW_24731	39	70.90909091	*S.bongori*
STMMW_24741	39	70.90909091	*S.bongori*

STMMW_24751	39	70.90909091	*S.bongori*
STMMW_24761	39	70.90909091	*S.bongori*
STMMW_24771	39	70.90909091	*S.bongori*
STMMW_24781	39	70.90909091	*S.bongori*
STMMW_24791	39	70.90909091	*S.bongori*
STMMW_24801	39	70.90909091	*S.bongori*
STMMW_24821	39	70.90909091	*S.bongori*
STMMW_24831	39	70.90909091	*S.bongori*
STMMW_24841	39	70.90909091	*S.bongori*
STMMW_24851	39	70.90909091	*S.bongori*
STMMW_24861	39	70.90909091	*S.bongori*
STMMW_24871	39	70.90909091	*S.bongori*
STMMW_24881	39	70.90909091	*S.bongori*
STMMW_24891	39	70.90909091	*S.bongori*
STMMW_31021	51	92.72727273	*S.bongori*
STMMW_03311	53	96.36363636	*S.bongori*
SPAB_04503	55	100	*S.bongori*
STM4351	55	100	*S.bongori*
STMMW_05791	55	100	*S.bongori*
STMMW_06111	55	100	*S.bongori*
STMMW_06121	55	100	*S.bongori*
STMMW_06131	55	100	*S.bongori*
STMMW_06141	55	100	*S.bongori*
STMMW_06151	55	100	*S.bongori*
STMMW_06161	55	100	*S.bongori*
STMMW_06171	55	100	*S.bongori*
STMMW_06181	55	100	*S.bongori*
STMMW_06201	55	100	*S.bongori*
STMMW_12591	55	100	*S.bongori*
STMMW_13961	55	100	*S.bongori*
STMMW_13971	55	100	*S.bongori*
STMMW_13981	55	100	*S.bongori*
STMMW_13991	55	100	*S.bongori*
STMMW_14001	55	100	*S.bongori*
STMMW_14021	55	100	*S.bongori*
STMMW_14031	55	100	*S.bongori*
STMMW_14041	55	100	*S.bongori*
STMMW_14051	55	100	*S.bongori*
STMMW_14061	55	100	*S.bongori*
STMMW_14071	55	100	*S.bongori*
STMMW_14081	55	100	*S.bongori*
STMMW_14091	55	100	*S.bongori*
STMMW_14101	55	100	*S.bongori*
STMMW_14111	55	100	*S.bongori*
STMMW_14121	55	100	*S.bongori*
STMMW_14131	55	100	*S.bongori*
STMMW_14141	55	100	*S.bongori*

STMMW_14151	55	100	*S.bongori*
STMMW_14161	55	100	*S.bongori*
STMMW_14171	55	100	*S.bongori*
STMMW_14181	55	100	*S.bongori*
STMMW_14191	55	100	*S.bongori*
STMMW_14201	55	100	*S.bongori*
STMMW_14211	55	100	*S.bongori*
STMMW_14221	55	100	*S.bongori*
STMMW_14231	55	100	*S.bongori*
STMMW_14241	55	100	*S.bongori*
STMMW_14251	55	100	*S.bongori*
STMMW_14261	55	100	*S.bongori*
STMMW_14271	55	100	*S.bongori*
STMMW_15861	55	100	*S.bongori*
STMMW_19431	55	100	*S.bongori*
STMMW_20501	55	100	*S.bongori*
STMMW_21691	55	100	*S.bongori*
STMMW_23791	55	100	*S.bongori*
STMMW_23801	55	100	*S.bongori*
STMMW_23811	55	100	*S.bongori*
STMMW_23821	55	100	*S.bongori*
STMMW_23831	55	100	*S.bongori*
STMMW_24261	55	100	*S.bongori*
STMMW_27411	55	100	*S.bongori*
STMMW_27421	55	100	*S.bongori*
STMMW_27431	55	100	*S.bongori*
STMMW_27441	55	100	*S.bongori*
STMMW_29361	55	100	*S.bongori*
STMMW_29371	55	100	*S.bongori*
STMMW_29381	55	100	*S.bongori*
STMMW_29391	55	100	*S.bongori*
STMMW_29401	55	100	*S.bongori*
STMMW_29411	55	100	*S.bongori*
STMMW_29421	55	100	*S.bongori*
STMMW_30841	55	100	*S.bongori*
STMMW_30851	55	100	*S.bongori*
STMMW_30861	55	100	*S.bongori*
STMMW_30871	55	100	*S.bongori*
STMMW_30881	55	100	*S.bongori*
STMMW_30891	55	100	*S.bongori*
STMMW_33241	55	100	*S.bongori*
STMMW_37801	55	100	*S.bongori*
STMMW_37811	55	100	*S.bongori*
STMMW_37821	55	100	*S.bongori*
STMMW_37831	55	100	*S.bongori*
STMMW_42281	55	100	*S.bongori*
STMMW_42301	55	100	*S.bongori*

STMMW_42311	55	100	*S.bongori*
STMMW_24431	6	22.22222222	VI
STMMW_36341	6	22.22222222	VI
STMMW_16601	26	96.2962963	VI
STMMW_16611	26	96.2962963	VI
STMMW_27951	26	96.2962963	VI
STMMW_27961	26	96.2962963	VI
STMMW_27971	26	96.2962963	VI
STMMW_11061	27	100	VI
STMMW_11071	27	100	VI
STMMW_12581	27	100	VI
STMMW_18001	27	100	VI
STMMW_27481	27	100	VI
STMMW_30841	27	100	VI
STMMW_30851	27	100	VI
STMMW_30861	27	100	VI
STMMW_30871	27	100	VI
STMMW_30881	27	100	VI
STMMW_30891	27	100	VI
STMMW_31021	27	100	VI
STMMW_31261	27	100	VI
STMMW_33241	27	100	VI
STMMW_37631	27	100	VI
STMMW_42771	27	100	VI
STM3012	22	100	VII
STMMW_15051	22	100	VII
STMMW_16181	22	100	VII
STMMW_24731	22	100	VII
STMMW_24741	22	100	VII
STMMW_24751	22	100	VII
STMMW_24761	22	100	VII
STMMW_24771	22	100	VII
STMMW_24781	22	100	VII
STMMW_24791	22	100	VII
STMMW_24801	22	100	VII
STMMW_24821	22	100	VII
STMMW_24831	22	100	VII
STMMW_24841	22	100	VII
STMMW_24851	22	100	VII
STMMW_24861	22	100	VII
STMMW_24871	22	100	VII
STMMW_24881	22	100	VII
STMMW_24891	22	100	VII
STMMW_26851	22	100	VII
STMMW_26861	22	100	VII
STMMW_27041	22	100	VII
STMMW_27051	22	100	VII

STMMW_27061	22	100	VII
STMMW_42281	22	100	VII
STMMW_42301	22	100	VII
STMMW_42311	22	100	VII
STMMW_42331	22	100	VII
STMMW_42391	22	100	VII
