# Supplementary material for: A proposed core genome scheme for analyses of the Salmonella genus
Source: Genomics. 2020 Jan;112(1):371–8. doi: 10.1016/j.ygeno.2019.02.016 (PMC6978875; doi:10.1016/j.ygeno.2019.02.016)
Supplement: Supplementary Table S5 — Table showing the 252 loci to be removed from the EBcgMLSTv2.0. The loci described here were absent in over 2% of at least one of the subspecies. All of the loci were present in over 98% of subspecies I, while the non-subspecies I analyses found between 10 and 108 loci needed to be removed per subspecies. 98 of these loci met the required threshold for removal (2%) in multiple subspecies. These loci were removed from EBcgMLSTv2.0 to create the genus core genome scheme proposed here. [file mmc5.pdf]

locus	subspecies
STMMW_11031	II
STMMW_23611	II
STMMW_23621	II
STMMW_23641	II
STMMW_23651	II
STMMW_23661	II
STMMW_23671	II
STMMW_42501	II
STMMW_43331	II
STMMW_12521	II
STMMW_12541	II
STMMW_12562	II
STMMW_15321	II
STMMW_15331	II
STMMW_15341	II
STMMW_15351	II
STMMW_15361	II
STMMW_15371	II
STMMW_18441	II
STMMW_28291	II
STMMW_28371	II
STMMW_34021	II
STM1092	II
STMMW_12561	II
STMMW_28381	II
STMMW_28391	II
STMMW_28411	II
STMMW_37041	II
STMMW_37051	II
STMMW_12551	II
STMMW_37061	II
STMMW_37071	II
STMMW_37081	II
STMMW_42081	II/IIIa
STMMW_42091	II/IIIa
STMMW_42101	II/IIIa/IIIb
STMMW_42641	II/IIIa/IIIb
STMMW_42651	II/IIIa/IIIb
STMMW_44481	II/IIIa/IV/*S.bongori*
STMMW_11021	II/*Novel_B*
STMMW_28741	II/Novel_C
STMMW_28751	II/Novel_C
STMMW_13981	II/*S.bongori*
STMMW_13991	II/*S.bongori*
STMMW_14001	II/*S.bongori*
STMMW_14021	II/*S.bongori*

STMMW_14031	II/*S.bongori*
STMMW_14041	II/*S.bongori*
STMMW_14051	II/*S.bongori*
STMMW_14061	II/*S.bongori*
STMMW_14071	II/*S.bongori*
STMMW_14081	II/*S.bongori*
STMMW_14091	II/*S.bongori*
STMMW_14101	II/*S.bongori*
STMMW_14111	II/*S.bongori*
STMMW_14121	II/*S.bongori*
STMMW_14131	II/*S.bongori*
STMMW_14141	II/*S.bongori*
STMMW_14151	II/*S.bongori*
STMMW_14161	II/*S.bongori*
STMMW_14171	II/*S.bongori*
STMMW_14181	II/*S.bongori*
STMMW_14191	II/*S.bongori*
STMMW_14201	II/*S.bongori*
STMMW_14211	II/*S.bongori*
STMMW_14221	II/*S.bongori*
STMMW_14231	II/*S.bongori*
STMMW_14241	II/*S.bongori*
STMMW_14251	II/*S.bongori*
STMMW_14261	II/*S.bongori*
STMMW_21691	II/*S.bongori* /Novel_B
STMMW_11061	II/VI
STMMW_11071	II/VI
STMMW_30841	II/VI/*S.bongori* /Novel_C
STMMW_30851	II/VI/*S.bongori* /Novel_C
STMMW_30861	II/VI/*S.bongori* /Novel_C
STMMW_30871	II/VI/*S.bongori* /Novel_C
STMMW_30881	II/VI/*S.bongori* /Novel_C
STMMW_30891	II/VI/*S.bongori* /Novel_C
STMMW_00091	IIIa
STMMW_00101	IIIa
STMMW_01611	IIIa
STMMW_01621	IIIa
STMMW_04911	IIIa
STMMW_07481	IIIa
STMMW_07491	IIIa
STMMW_07501	IIIa
STMMW_09341	IIIa
STMMW_09351	IIIa
STMMW_09361	IIIa
STMMW_09971	IIIa
STMMW_11491	IIIa
STMMW_11501	IIIa

STMMW_12701	IIIa
STMMW_12931	IIIa
STMMW_13251	IIIa
STMMW_13741	IIIa
STMMW_14811	IIIa
STMMW_15771	IIIa
STMMW_15791	IIIa
STMMW_15801	IIIa
STMMW_16131	IIIa
STMMW_16991	IIIa
STMMW_21731	IIIa
STMMW_21741	IIIa
STMMW_21751	IIIa
STMMW_22081	IIIa
STMMW_22091	IIIa
STMMW_22181	IIIa
STMMW_22191	IIIa
STMMW_23211	IIIa
STMMW_23221	IIIa
STMMW_23231	IIIa
STMMW_25861	IIIa
STMMW_28761	IIIa
STMMW_28771	IIIa
STMMW_28791	IIIa
STMMW_31491	IIIa
STMMW_32331	IIIa
STMMW_34703	IIIa
STMMW_35301	IIIa
STMMW_35741	IIIa
STMMW_35751	IIIa
STMMW_35761	IIIa
STMMW_36521	IIIa
STMMW_37551	IIIa
STMMW_37561	IIIa
STMMW_37641	IIIa
STMMW_37651	IIIa
STMMW_42151	IIIa
STMMW_43841	IIIa
STMMW_43861	IIIa
STMMW_43881	IIIa
STMMW_43891	IIIa
STMMW_43901	IIIa
STMMW_43921	IIIa
STMMW_44141	IIIa
STMMW_44541	IIIa
STMMW_44881	IIIa
STMMW_22041	IIIa

STMMW_21301	IIIa/IIIb/IV
STMMW_21311	IIIa/IIIb/IV
STMMW_21331	IIIa/IIIb/IV
STMMW_21341	IIIa/IIIb/IV
STMMW_16161	IIIa/Novel_B
STMMW_05791	IIIa/*S.bongori*
STMMW_06201	IIIa/*S.bongori*
STMMW_19431	IIIa/*S.bongori*
STMMW_24261	IIIa/*S.bongori*
STMMW_06121	IIIa/*S.bongori* /Novel_B
STMMW_06161	IIIa/*S.bongori* /Novel_B
STMMW_06171	IIIa/*S.bongori* /Novel_B
STMMW_06181	IIIa/*S.bongori* /Novel_B
STMMW_37631	IIIa/VI
STMMW_31021	IIIa/VI/*S.bongori*
STM3012	IIIa/VII
STMMW_27411	IIIb/*S.bongori*
STMMW_27421	IIIb/*S.bongori*
STMMW_27431	IIIb/*S.bongori*
STMMW_27441	IIIb/*S.bongori*
STMMW_01491	IV
STMMW_20411	IV
STMMW_01221	IV
STMMW_18431	IV
STMMW_20501	IV/*S.bongori*
STMMW_04631	Novel_B
STMMW_08391	Novel_B
STMMW_08401	Novel_B
STMMW_08411	Novel_B
STMMW_08421	Novel_B
STMMW_08431	Novel_B
STMMW_24971	Novel_B
STMMW_32761	Novel_C
SPAB_04503	*S.bongori*
STM4351	*S.bongori*
STMMW_03311	*S.bongori*
STMMW_11091	*S.bongori*
STMMW_11101	*S.bongori*
STMMW_11111	*S.bongori*
STMMW_11121	*S.bongori*
STMMW_11131	*S.bongori*
STMMW_11141	*S.bongori*
STMMW_11151	*S.bongori*
STMMW_11161	*S.bongori*
STMMW_11171	*S.bongori*
STMMW_11181	*S.bongori*
STMMW_12591	*S.bongori*

STMMW_13961	*S.bongori*
STMMW_13971	*S.bongori*
STMMW_14271	*S.bongori*
STMMW_15861	*S.bongori*
STMMW_29361	*S.bongori*
STMMW_29371	*S.bongori*
STMMW_29381	*S.bongori*
STMMW_29391	*S.bongori*
STMMW_29401	*S.bongori*
STMMW_29411	*S.bongori*
STMMW_29421	*S.bongori*
STMMW_37801	*S.bongori*
STMMW_37811	*S.bongori*
STMMW_37821	*S.bongori*
STMMW_37831	*S.bongori*
STMMW_06111	*S.bongori* /Novel_B
STMMW_06131	*S.bongori* /Novel_B
STMMW_06141	*S.bongori* /Novel_B
STMMW_06151	*S.bongori* /Novel_B
STMMW_23791	*S.bongori* /Novel_C
STMMW_23801	*S.bongori* /Novel_C
STMMW_23811	*S.bongori* /Novel_C
STMMW_23821	*S.bongori* /Novel_C
STMMW_23831	*S.bongori* /Novel_C
STMMW_24731	*S.bongori* /VII
STMMW_24741	*S.bongori* /VII
STMMW_24751	*S.bongori* /VII
STMMW_24761	*S.bongori* /VII
STMMW_24771	*S.bongori* /VII
STMMW_24781	*S.bongori* /VII
STMMW_24791	*S.bongori* /VII
STMMW_24801	*S.bongori* /VII
STMMW_24821	*S.bongori* /VII
STMMW_24831	*S.bongori* /VII
STMMW_24841	*S.bongori* /VII
STMMW_24851	*S.bongori* /VII
STMMW_24861	*S.bongori* /VII
STMMW_24871	*S.bongori* /VII
STMMW_24881	*S.bongori* /VII
STMMW_24891	*S.bongori* /VII
STMMW_42281	*S.bongori* /VII
STMMW_42301	*S.bongori* /VII
STMMW_42311	*S.bongori* /VII
STMMW_12581	VI
STMMW_18001	VI
STMMW_24431	VI
STMMW_27481	VI

STMMW_27951	VI
STMMW_27961	VI
STMMW_27971	VI
STMMW_31261	VI
STMMW_36341	VI
STMMW_42771	VI
STMMW_16601	VI/Novel_B/Novel_C
STMMW_16611	VI/Novel_B/Novel_C
STMMW_33241	VI/*S.bongori* /Novel_B
STMMW_15051	VII
STMMW_16181	VII
STMMW_26851	VII
STMMW_26861	VII
STMMW_27041	VII
STMMW_27051	VII
STMMW_27061	VII
STMMW_42331	VII
STMMW_42391	VII
